# Genome-wide screening for deubiquitinase subfamily identifies ubiquitin-specific protease 49 as a novel regulator of odontogenesis

**DOI:** 10.1038/s41418-022-00956-7

**Published:** 2022-03-10

**Authors:** Kamini Kaushal, Eun-Jung Kim, Apoorvi Tyagi, Janardhan Keshav Karapurkar, Saba Haq, Han-Sung Jung, Kye-Seong Kim, Suresh Ramakrishna

**Affiliations:** 1 Graduate School of Biomedical Science and Engineering, Hanyang University, Seoul, 04763 South Korea; 2 Division in Anatomy and Developmental Biology, Department of Oral Biology, Taste Research Center, Oral Science Research Center, BK21 FOUR Project, Yonsei University College of Dentistry, Seoul, 03722 South Korea; 3 Department of Life Science, College of Natural Sciences, Hanyang University, Seoul, 04763 South Korea; 4 College of Medicine, Hanyang University, Seoul, 04763 South Korea

**Keywords:** Proteases, Deubiquitylating enzymes

## Abstract

Proteins expressed by the paired box gene 9 (PAX9) and Msh Homeobox 1 (MSX1) are intimately involved in tooth development (odontogenesis). The regulation of PAX9 and MSX1 protein turnover by deubiquitinating enzymes (DUBs) plausibly maintain the required levels of PAX9 and MSX1 during odontogenesis. Herein, we used a loss-of-function CRISPR-Cas9-mediated DUB KO library kit to screen for DUBs that regulate PAX9 and MSX1 protein levels. We identify and demonstrate that USP49 interacts with and deubiquitinates PAX9 and MSX1, thereby extending their protein half-lives. On the other hand, the loss of USP49 reduces the levels of PAX9 and MSX1 proteins, which causes transient retardation of odontogenic differentiation in human dental pulp stem cells and delays the differentiation of human pluripotent stem cells into the neural crest cell lineage. USP49 depletion produced several morphological defects during tooth development, such as reduced dentin growth with shrunken enamel space, and abnormal enamel formation including irregular mineralization. In sum, our results suggest that deubiquitination of PAX9 and MSX1 by USP49 stabilizes their protein levels to facilitate successful odontogenesis.

## Introduction

Tooth development, or odontogenesis, involves reciprocal interactions between the dental epithelium and the underlying neural crest-derived mesenchyme. Tooth agenesis is primarily caused by mutations in genes encoding the transcription factors involved in tooth signaling as well as environmental factors, such as irradiation, chemotherapy, and drugs [1–6]. To date, reports on the genetic progress of tooth development have revealed more than 200 candidate genes associated with hypodontia [7, 8]. Among them, the transcription factors, paired box gene 9 (*PAX9*) and Msh Homeobox 1 (*MSX1*), have emerged as genes likely to be responsible for isolated/non-syndromic hypodontia and oligodontia [1, 2, 9, 10].

*PAX9* regulates cellular pluripotency and differentiation during embryonic patterning and organogenesis, and *MSX1* is essential for epithelial-to-mesenchymal transition during organogenesis [11, 12]. The early stages of tooth development show co-expression of the *Msx1* and *Pax9* genes in the dental mesenchyme. This finding was supported by tooth development studies in mice, which reported that the homozygous deletion of *Msx1* and *Pax9* resulted in tooth organ arrest at the bud stage [13, 14]. Pax9 interacts with Msx1 at both the gene and protein levels and transactivates Msx1 and bone morphogenetic protein 4 expression, indicating that Pax9 is a critical upstream regulator of Msx1 during tooth development [15]. Along with genetic regulation, various epigenetic factors are associated with variations in tooth number, size, and shape [16].

Given that both PAX9 and MSX1 proteins are required for the progression of tooth development [15], regulating their protein turnover is a critical factor. The reversal of PAX9 and MSX1 protein degradation by deubiquitinating enzymes (DUBs) might therefore play a key role in odontogenesis.

DUBs are proteases that reverse the ubiquitination process by detaching the ubiquitin moiety from the target protein to maintain steady-state levels. Ubiquitin-specific peptidases (USPs) constitute the largest subfamily of DUBs and are involved in cellular functions, such as cell signaling, DNA repair, cell-cycle regulation, and stem cell differentiation [17–22]. Extensive research has shown the importance of DUBs in bone remodeling [23]. USP4 interacts with and deubiquitinates several substrates, such as Disheveled, Nemo-like kinase, T-cell factor 4, and β-catenin, which are key components of the Wnt/β-catenin signaling pathway, an important pathway for osteoblast differentiation [24–27]. Likewise, USP15 interacts with β-catenin and regulates osteoblasts via the Wnt/β-catenin signaling pathway [28]. Similarly, USP9x and USP11 interact and deubiquitinate Smad4 and ALK5, respectively, [29, 30] to regulate the transforming growth factor/BMP cell signaling pathways necessary for osteogenic differentiation and bone formation. However, the importance of DUBs in tooth formation has not yet been reported.

Therefore, we initiated this study to identify DUBs for human PAX9 and MSX1 proteins using a clustered regularly interspaced palindromic repeat/caspase 9 (CRISPR/Cas9)-based DUB-knockout screening kit by performing genome-scale knockout of all the genes that encode USPs [31, 32]. This loss-of-function-based screening led to the identification of USP49 as a regulator of PAX9 and MSX1 protein levels during tooth development.

## Materials and methods

### Plasmids, sgRNAs, and shRNAs

A mammalian expression vector encoding Myc-Flag-tagged PAX9 was purchased from OriGene (Cat. no. RC200380, Rockville, USA). PAX9 was further subcloned into a Myc-tagged vector. Flag-tagged MSX1 was kindly provided by Prof. Kyoungsook Park (Sungkyunkwan University, South Korea) [33] and further subcloned into the pcDNA 3.1 6XMyc-vector. Flag-HA tagged USP49 (Cat. no. 22586), HA-tagged ubiquitin (Cat. no. 18712), and Cas9-2A-GFP (Cat. no. 44719) were purchased from Addgene. pcDNA 3.1 USP49 and a catalytic mutant of Flag-USP49 (C262A point mutation) were kindly provided by Prof. Hengbin Wang (University of Alabama, Birmingham, USA). Flag-tagged USP49 was subcloned into an eGFP_N1 vector (Genbank Accession No. U55762, CLONTECH). The N-terminus USP49 (1-250 aa) encoding Zinc-finger ubiquitin binding (ZnF-UBP) domain and C-terminus USP49 (251-640 aa) encoding ubiquitin C-terminal hydrolase (UCH) domain were cloned into the pCS4 3XFlag-vector.

For DUB screening, we used a plasmid encoding Cas9-2a-mRFP-2a-PAC (puromycin N-acetyl-transferase, puromycin resistance gene) and a plasmid encoding single guide RNA (sgRNA); both constructs were purchased from Toolgen (Seoul, South Korea). The sgRNA target sequences were designed using bioinformatics tools (www.broadinstitute.org) and cloned into the vectors as previously described [34]. Briefly, oligonucleotides containing each target sequence were synthesized (Bioneer, Seoul, South Korea), and T4 polynucleotide kinase was used to add terminal phosphates to the annealed oligonucleotides (Bio-Rad, CA, USA). The vector was digested with *BsaI* restriction enzyme and ligated with the annealed oligonucleotides. Retroviral vectors along with packaging plasmids were kindly provided by Prof. Chang-Hwan Park and short hairpin RNA (shRNA) lentiviral vector constructs along with the packaging plasmids were kindly provided by Prof. Chung Hee Yong (both from Hanyang University, Seoul, South Korea). Target sequences for the sgRNA and shRNA are listed in Supplementary Tables S1, S2, respectively.

### Cell culture

Human embryonic kidney (HEK293) cell line purchased from Korean Cell Line Bank (KCLB: 21573) were cultured in DMEM (GIBCO BRL, Rockville, MD, USA) supplemented with 10% fetal bovine serum (FBS; GIBCO) and 1% penicillin and streptomycin (GIBCO) at 37 °C in a humidified atmosphere with 5% CO_2_. The human dental pulp stem cells (hDPSCs) cell line purchased from Lonza (Cat. no. PT-3005) were cultured in DPSCBM and 1% penicillin and streptomycin (GIBCO) at 37 °C in a humidified atmosphere with 5% CO_2_. The cells were passaged every 2–3 days depending on cell confluence. Human embryonic stem cells (hESCs-CHA15; established by CHA University, Seoul, South Korea) were cultured on BD Matrigel (Cat. no. 356230, BD Biosciences) in mTeSR™1 complete medium (Cat. no. 85850, Stem Cell Technologies) supplemented with 10 µM Y-27632 dihydrochloride (Cat. No. 1254, TOCRIS, Bristol, UK). Human-induced pluripotent stem cells (hiPSCs) cell line purchased from GIBCO, USA (Cat. No. A18943PIS) were maintained on Geltrex^TM^ (Cat. no. 12760-013, GIBCO)-coated dishes in StemFlex^TM^ medium (Cat. no. A3349401, GIBCO) supplemented with 10 µM Y-27632 dihydrochloride. We used hDPSCs for detecting endogenous USP49, PAX9, and MSX1 proteins, while HEK293 cells for detecting exogenous USP49, PAX9, and MSX1 proteins. The cell lines purchased from authorized repositories authentication was validated by STR profiling in the respective cell banks. Cell lines were treated with Plasmocin-mycoplasma eliminating reagent (Cat. No. ant-mpt1, InvivoGen) before the experiment according to the manufacturer’s instructions. Mycoplasma contamination was checked by MycoAlert™ Mycoplasma Detection Kit (Cat. No. LT07-118, Lonza Bioscience).

### Transient transfection and stable cell line production

For transient transfection, HEK293 cells were transfected with plasmids using polyethyleneimine (Polysciences, Warrington, PA, USA) according to the manufacturer’s protocol. The hDPSCs were transfected with Lipofectamine 3000 (Cat. no. L3000001, Thermo Fisher Scientific).

Retroviruses were produced by co-transfecting Flag-USP49, a pMD packaging plasmid containing Gag-Pol and a VSV-G envelope plasmid into HEK293 cells in a 3:2:1 ratio. shRNA lentiviruses were produced by co-transfecting the shRNA construct with packaging plasmids (pLP1, pLP2, and pLP-VSVG) into HEK293 cells in a 4:1:1:1 ratio. Cell supernatants were harvested 48 h after transfection and were either used to infect cells or stored at −80 °C. Cells were infected at a low confluence (20%) for 6 h with lentiviral and retroviral supernatants diluted 1:1 with normal culture medium in the presence of 10 ng/mL of polybrene (Sigma-Aldrich) to obtain stable cell lines of hDPSCs expressing Flag-USP49 or shRNA targeting *USP49*. After 48 h of infection, the cells were placed under puromycin selection for 2 days and then passaged before use.

### Electroporation

Electroporation was used for tooth germ transfection under a microscope. A microcapillary needle was used to inject about 1 µg/µL of DNA into the dental mesenchyme, after which 50 ms current pulses of 12 V were applied with an electroporator. The control group was electroporated with an empty sgRNA vector and pCAG-Cas9-GFP (Cat. no. 44719, Addgene). The experimental group was electroporated with sgRNA targeting *USP49* and pCAG-Cas9-GFP. Under the microscope, GFP-expressing samples were successfully transfected, therefore, only GFP-expressing samples were selected and used for analysis (*n* = 5).

### Antibodies and reagents

Mouse monoclonal antibodies against Flag (Anti-DDDDK-tag, M185-3L, 1:1,000) were purchased from MBL Life Science, and c-Myc (SC-40, 1:1,000), ubiquitin (SC-8017, 1:1,000), HA (SC-7392, 1:1000), β-actin (SC-47778, 1:1,000), GAPDH (SC-32233, 1:1,000), GFP (SC-996, 1:1,000), anti-Amelx (SC-365284, 1:100), anti-DSPP (SC-73632, 1:100), PAX9 (SC-56823, 1:500), anti-Sox2 (SC-365823, 1:250), NGFR p75 (B-1) (SC-271708, 1:150) and normal mouse IgG (SC-2025, 1:1,000) were purchased from Santa Cruz Biotech. MSX1 (H00004487-M11, 1: 1,000; Novus Biologicals) and rabbit polyclonal antibodies against MSX1 (SC-15395, 1:500), PAX9 (CST, D9F1N, 1: 1,000; Cell Signaling Technology), USP49 (180661-AP 1: 1,000; Protein Tech), anti-nestin (MAB1259; 1:500; R&D systems), anti-Oct4 (ab18976; 1:500; Abcam Inc.), anti-SSEA4 (90231, 1:250; Millipore), anti-Nanog (ab80892; 1:400; Abcam Inc.), anti-LHX6 (ARP32553_T100; 1:250; Aviva Systems Biology) and 488/594-conjugated secondary antibodies (Cat. no. A21207; Cat. no. A21203, 1:200; Life Technologies) were used. Protein A/G Plus Agarose beads (SC-2003; Santa Cruz Biotech); protease inhibitor cocktail (Cat. no. 11836153001; Roche), IP lysis buffer (Cat. no. 87787; Thermo Fisher), cell lysis buffer (Cat. no. R2002; Biosesang), Protein 5X sample buffer (Cat no. EBA-1052; Elpis biotech); the proteasomal inhibitor MG132 (Cat. no. S2619; Selleckchem), protein translation inhibitor cycloheximide (CHX; Cat. no. 239765; Merck), puromycin (Cat. no. 12122530, GIBCO), dexamethasone (Cat. no. D4902, Sigma-Aldrich), β-glycerophosphate (Cat. no. G9891, Sigma-Aldrich), L-ascorbic acid (Cat. no. A8960, Sigma-Aldrich), Hoechst dye (Cat. No. 33258; Sigma), DAPI (Cat. no. H-1200, Vector Laboratories) and To-Pro3 (Cat. no. T3605, 1:1000; Thermo Fisher) were purchased from the noted manufacturers.

### T7 endonuclease 1 assay

The T7 endonuclease 1 (T7E1) assay was performed as previously described [35, 36]. Genomic DNA was isolated using DNeasy Blood & Tissue kits (Qiagen, Hilden, Germany) according to the manufacturer’s instructions. The region of DNA containing the nuclease target site was PCR-amplified using hemi-nested or nested primers. Amplicons were denatured by heating and annealed to form heteroduplex DNA, which was then treated with 5 units of T7E1 (New England Biolabs, MA, USA) for 15–20 min at 37 °C, followed by 2% agarose gel electrophoresis. Mutation frequencies were calculated based on band intensity using ImageJ software and the following equation: mutation frequency (%) = 100 × (1 − [1 − fraction cleaved]^1/2^), where the fraction cleaved was the total relative density of the cleavage bands divided by the sum of the relative density of the cleavage and uncut bands. The oligonucleotide sequences used to get the PCR amplicon for the T7E1 assay are listed in Supplementary Table S3. The amplicon size of the *USP49* gene and expected cleavage sizes after the T7E1 assay are summarized in Supplementary Table S4.

### Immunoprecipitation

For co-immunoprecipitation, the cells were harvested and lysed for 20 min in co-immunoprecipitation lysis buffer containing 25 mM Tris-HCl (pH 7.4), 150 mM sodium chloride, 1 mM EDTA, 1% NP-40, 5% glycerol, 1 mM PMSF along with protease inhibitor cocktail. Then, 2–3 mg of cell lysates were immunoprecipitated with the respective antibodies at 4 °C overnight and combined with 25 μL of protein agarose beads at 4 °C for 2–3 h. The beads were washed with lysis buffer and eluted in 2× SDS sample loading buffer (5X SDS sample loading buffer contains 4% SDS, 20% glycerol, 10% 2-mercaptoethanol, 0.004% bromophenol blue, and 0.125 M Tris-HCl (pH 6.8) and boiled at 95–100 °C for 5 min. The samples were then loaded onto SDS-PAGE gels and analyzed by Western blotting using a ChemiDoc Imaging System. Mouse IgG (ab-99697) and rabbit IgG (CST- 58802 S) light chain-specific secondary antibodies were used to prevent interference from heavy and light immunoglobulin chains in the binding assay.

### Tandem ubiquitin-binding entities assay

The ubiquitination status of PAX9 and MSX1 was determined using a tandem ubiquitin-binding entities (TUBEs) assay (Cat. no. UM402, LifeSensors, PA, USA). hDPSCs were treated with 5 ng/mL of interleukin-1β for 6 h before harvesting the cells for protein extraction. The cells were lysed for 20 min in cell lysis buffer containing 50 mM Tris-HCl, pH 7.4, 0.15 M NaCl, 1 mM EDTA, 1% NP-40, 10% glycerol along with a protease inhibitor cocktail. The beads were washed with 20 mM Tris, 150 mM NaCl, 0.1% (w/v) Tween^®^ 20 detergent and eluted in 2× SDS sample loading buffer (5× SDS sample loading buffer contains 4% SDS, 20% glycerol, 10% 2-mercaptoethanol, 0.004% bromophenol blue, 0.125 M Tris-HCl (pH 6.8) and boiled at 95–100 °C for 5 min. Samples were immunoblotted with PAX9 and MSX1 antibodies and analyzed by Western blotting using a ChemiDoc Imaging System.

### Deubiquitination assay

The DUB activity of USP49 on endogenous and exogenous PAX9 or MSX1 proteins were determined in hDPSCs and HEK293 cells, respectively. After 48 h, cells were treated with MG132 (5 µM/mL for 6 h) and harvested. The cells were lysed for 20 min in denaturing lysis buffer containing 150 mM sodium chloride, 1% Triton X-100, 1% sodium deoxycholate, 1% SDS, 50 mM Tris-HCl (pH 7.4), 2 mM EDTA, 1 mM PMSF along with protease inhibitor cocktail. Then, 2–3 mg of cell lysates were immunoprecipitated with the respective antibodies at 4 °C overnight and combined with 25 μL of protein agarose beads at 4 °C for 2–3 h. The beads were washed with lysis buffer and eluted in 2× SDS sample loading buffer (5× SDS sample loading buffer contains 4% SDS, 20% glycerol, 10% 2-mercaptoethanol, 0.004% bromophenol blue and 0.125 M Tris-HCl (pH 6.8) and boiled at 95–100 °C for 5 min. The samples were then loaded onto SDS-PAGE gels and analyzed by Western blotting using ubiquitin antibody.

### Immunohistochemistry

Cultured tissues were fixed with 4% paraformaldehyde (PFA) (Wako, Osaka, Japan) in 0.01 M phosphate-buffered saline (PBS, pH 7.4) overnight at 4 °C. After embedding them in paraffin (Leica Biosystems, Maryland Heights, MO), we sectioned the samples at a thickness of 4 μm. Some sections were picked randomly and stained with hematoxylin and eosin (H&E) for histological observation.

### Duolink proximity ligation assay

The interaction between USP49 and PAX9 or USP49 and MSX1 was observed using a Duolink in situ proximity ligation assay (PLA) kit (DUO92101, Sigma-Aldrich). hDPSCs were fixed in 4% PFA for 10 min at room temperature before being blocked with a blocking solution. The cells were treated with primary antibodies targeting USP49, PAX9, and MSX1 for 1 h at 37 °C, followed by incubation with PLA probes for 1 h at 37 °C in a humidified chamber. After three washes, a ligation-ligase solution was added and incubated for 30 min at 37 °C. The slides were incubated for 100 min in an amplified polymerase solution at 37 °C in the dark. Finally, the cells were stained with a mounting medium containing DAPI. A Leica fluorescence microscope was used to capture the fluorescence images (Leica, DM 5000B; Leica CTR 5000; Wetzlar, Germany).

### Immunofluorescence

For the co-localization studies, hDPSCs stably expressing Flag-USP49 were seeded in a 24-well plate and incubated at 37 °C in a humidified atmosphere with 5% CO_2_ for 36 h. For the pluripotency and differentiation studies, hPSCs and neural crest-like cells (NCLCs) were seeded in four-well plates and incubated at 37 °C in a humidified atmosphere with 5% CO_2_ for 48 h. After a PBS wash, the cells were fixed for 15 min in 4% PFA and permeabilized with 0.1% Triton X-100 in PBS for 10 min. For immunofluorescence staining of the different stages of tooth development, sections of wax-embedded tissue specimens were boiled in 10 mM citrate buffer (pH 6.0) for 20 min and then cooled at room temperature for 20 min. The slides were incubated with the respective primary antibodies at 4 °C overnight. After washing them with PBS, we incubated the slides with 1 μg/mL of Alexa Fluor 488/594-conjugated secondary antibodies and counterstained them using VECTASHIELD anti-fade mounting medium with DAPI and To-Pro for the cells and tissue sections, respectively. The stained immunofluorescence images were captured using a confocal laser microscope (LSM510META Ver. 3.2; Carl Zeiss, Oberkochen, Germany or DMi8; Leica, Wetzlar, Germany).

### Generation of USP49-knockout in human pluripotent stem cells and single cell-derived USP49-knockout clonal analysis

hESCs and hiPSCs were co-transfected with a plasmid encoding pCAG-Cas9-GFP and sgRNA targeting the *USP49* gene or non-targeting sgRNA (mock-control) at a 1:2 ratio using Lipofectamine Stem Transfection Reagent (Thermo Fisher Scientific, MA, USA), according to the manufacturer’s instructions. After 48 h, both the mock-control and USP49-sgRNA transfected cells were sorted using a FACSAria III (BD Bioscience) and seeded into a 96-well plate for recovery. After three days, the cells were dissociated with a gentle cell dissociation reagent (Cat. no. 07174, STEMCELL Technologies Inc.), resuspended in StemFlex^TM^ medium, and seeded into 96-well plates at an average density of 25 cells/plate. Twelve days after the cell seeding, each well was microscopically evaluated, and single cell-derived, round, undifferentiated colonies were selected. Each selected colony was individually dissociated with a gentle dissociation buffer and reseeded into a 24-well plate. A small portion of the cells was screened for *USP49* gene disruption by the T7E1 assay. T7E1-positive clones were expanded and stored in a liquid nitrogen tank. Gene disruption was confirmed by sequencing the region that included the target sequence, as previously described [34]. Briefly, PCR amplicons with the included CRISPR-Cas9-target sites were cloned into the pGEM-T vector (Promega, Cat. no. A3600), and the cloned plasmids were sequenced using the same primers used for PCR amplification (Supplementary Table S3).

### Quantitative reverse transcription-PCR analysis

Total RNA was isolated using Trizol reagent (Favorgen, Kaohsiung, Taiwan). RNA pellets were resuspended in 30 μl of nuclease-free water, and the concentration was determined. 500 ng of total RNA was reverse transcribed using a SuperScript III First-Strand Synthesis System (Life Technologies, USA) with an oligo-dT primer, according to the manufacturer’s protocol. Quantitative PCR was performed in triplicate using Fast SYBR Green I Master Mix (Life Technologies) and a Step One Plus Real-Time PCR system (Life Technologies). Pluripotent and NCLC-specific gene primers were designed using the specifications listed in Supplementary Table S5. The relative expression of each gene was normalized to GAPDH as a control for all experiments. After DNA amplification, the expression levels were determined using GraphPad Prism 9 (GraphPad Software, San Diego, CA, USA).

### Odontogenic differentiation of hDPSCs

Mock-control hDPSCs; hDPSCs stably expressing Flag-USP49; sgRNA or shRNA targeting *USP49*; and N- or C-terminus USP49 transfected hDPSCs were seeded into six-well plates and allowed to reach 70% confluence. The cells were then incubated with osteogenic-induction medium (basal medium with 0.1 μM dexamethasone, 10 mM β-glycerophosphate, and 60 μM L-ascorbic acid) and 1% penicillin/streptomycin. The cells were cultured for 2 weeks for odontogenesis, and the induction medium was changed every other day.

### Alizarin red staining

hDPSC mineralization in the control and experimental groups was examined on days 3, 7, and 14 using alizarin red staining (ALZ). Odontogenic cultures were washed twice with PBS and fixed in 4% PFA for 15 min, and then alizarin red (Cat. no. A5533, Sigma Aldrich) staining was performed according to the manufacturer’s protocol. Red stains indicating calcium deposition were observed by phase-contrast microscopy. To quantify the calcium content, 1 mL of 10% cetylpyridinium chloride (CPC) (Sigma) was added to both the control and experimental samples and incubated for 20–30 min to elute the stain. 100 μL of the stain eluted in CPC was added to a 96-well plate, and a reading was taken at 550 nm using a spectrophotometer. A standard curve was prepared using ALZ and CPC. The calcium deposition is expressed as the molar equivalent of calcium. One mole of ALZ binds to two moles of calcium in an alizarin red S-calcium complex.

### Alkaline phosphatase staining

For odontogenic differentiation of hDPSCs, alkaline phosphatase (ALP) staining was performed on days 3, 7, and 14. Odontogenic cultures were washed twice with PBS and fixed in 4% PFA for 15 min. ALP (Cat. no. 86R-1KT, Sigma-Aldrich) staining was performed according to the manufacturer’s protocol. The ALP activity in the cells was quantified at different time points with an ALP Colorimetric Assay Kit (Abcam plc, Cambridge, UK) that used p-nitrophenyl phosphate (pNPP) as the phosphatase substrate. Briefly, cells from the control and experimental groups were harvested after 3, 7, and 14 days of culture in an osteogenic induction medium, and the cell lysate was prepared in ALP assay buffer. 80 µL of the cell lysate was mixed with 50 μl of pNPP in a 96-well plate and incubated for 1 h at room temperature in the dark. The reaction was then stopped by adding 20 µL of stop solution (3 N NaOH), and the plate was read at 415 nm using a spectrophotometer.

### DNA content for ALZ and ALP assays

The DNA content in the cell lysates prepared for the ALZ and ALP assays was measured using bisBenzimide fluorescent dye (Hoechst). Briefly, 50 μL of each cell lysate was added to separate 1.5 mL tubes containing 50 μL of assay buffer and 100 μL of Hoechst dye. A 0.1 μg/mL solution was used for the 3-day time point, and a 1 μg/mL solution was used for the later time points. The plate was incubated away from light for 10 min and then subjected to excitation at 350 nm; emissions were monitored at 450 nm with an EnVision system (PerkinElmer).

### Induction of neural crest stem cells from hESCs and hiPSCs

NCLCs were derived from hESCs and hiPSCs as described previously [37] with a slight modification (the scheme of differentiation for hESCs and hiPSCs is represented in Supplementary Fig. S13A). In brief, undifferentiated hESCs and hiPSCs were harvested using a gentle cell dissociation reagent and grown in suspension culture as embryoid bodies (EBs) for 5 days in Essential 6 medium (Cat. no. A116401, Thermo Fisher Scientific). The EBs were then transferred to BD Matrigel-coated dishes in a serum-free neural induction medium (N2B27) containing a 1:1 ratio of DMEM/F12 (Cat. no. 11320-033, GIBCO) and neurobasal medium (Cat. no. 21103049 GIBCO) supplemented with 0.5× B27 supplement (Cat. no. 17504-044, GIBCO), N2 supplement (Cat. no. 17502-048, GIBCO), 1× non-essential amino acids (Cat. no. 11140-050, GIBCO), 1× Glutamax (Cat. no. 35050061, GIBCO), 2-mercaptoethanol (Cat. no. M7522, Sigma-Aldrich), 20 ng/mL bFGF (Cat. no. AF-100-18B, PeproTech), 20 ng/mL EGF (Cat. no. 01-107, Sigma-Aldrich), 4 mg/mL insulin (Cat. no. 91077 C, Sigma-Aldrich) and penicillin-streptomycin (Cat. no. SV30010, HyClone). After 8–10 days, colonies with rosette structures were mechanically harvested, and the resulting NCLCs were passaged with a gentle cell dissociation reagent and plated onto fibronectin/poly-L-ornithine (FN/PLO)-coated plates. The NCLCs were maintained in N2B27 on FN/PLO-coated culture plates and passaged every 5 days. The expression of neural crest-related markers was evaluated using immunostaining, qRT-PCR, and Western blot analyses. Immunofluorescence intensity was calculated using Fiji ImageJ software and the following equation: Corrected total cell fluorescence = Integrated density – (area of selected cells X mean fluorescence of background reading), where the integrated density was the total relative fluorescence of the cells subtracted from the total background fluorescence. NCLCs that were homogeneously positive for p75 and NESTIN were selected for further studies.

### Animal studies

Adult Institute of Cancer Research (ICR) mice purchased from Koatech Co. (Pyeongtaek, Korea) were housed in a temperature-controlled room (22 °C) under artificial lighting (lights on from 05:00 to 17:00) and 55% relative humidity with access to food and water ad libitum. Embryos were obtained from time-mated pregnant mice. E0 was designated as the day on which the presence of a vaginal plug was confirmed. Embryos from each developmental stage (E12.5, E14.5, and E16.5) were used in this study. Embryos were decalcified in 10% EDTA, fixed in 4% PFA overnight, embedded in paraffin, and sectioned. All animal experiments were approved by Yonsei University Health System Institutional Animal Care and Use Committee in accordance with the Guide for the care and use of laboratory animals (National Research Council, USA). The animal study plan for these experiments (2018-0183) was reviewed and approved by this committee. All experiments were performed in accordance with the guidelines of this committee. No randomization of samples was performed in this study.

### In vitro culture and kidney transplantation

Tooth germ from the bud stage was isolated from ICR mouse mandibles and cultured in DMEM (GIBCO) containing 10% FBS at 37 °C and 5% CO_2_ for 2 days after electroporation. Six biological replicates per group (*n* = 6) were fixed with 4% PFA to determine the regulation of Pax9 and Msx1 expression upon Usp49 knockdown. Fifteen biological replicates per group (*n* = 15) were transplanted under the renal capsule of mice. Five mice were included in each group for renal transplantation. Healthy 6-week-old male nude mice were purchased (BALB/c Nu/Nu, Nara Biotech, Korea) and were anesthetized by carbonic acid gas, otherwise, no mice were excluded in this study. The recombinants were transplanted beneath the renal capsules for 4 weeks. After 4 weeks, the host mice were sacrificed, and their kidneys were dissected to obtain the calcified tissues. No randomization of samples was performed in this study.

### Statistical analysis

All results are presented as the means and standard deviations of at least three independent experiments for in vitro assays (*n* = 3) and at least five independent experiments for in vivo analysis (*n* = 5). Comparisons between two groups were analyzed using Student’s *t* test. Experiments involving three or more groups were analyzed by one-way or two-way analysis of variance (ANOVA) followed by Tukey’s post hoc test. A *P* value <0.05 was considered statistically significant. All statistical analyses were performed in GraphPad Prism 9 software.

## Results

### Genome-scale screening of the USP subfamily for PAX9 and MSX1 proteins using the DUB-knockout (DUB KO) library kit

To better understand the regulation of protein turnover of the PAX9 and MSX1 proteins during tooth development, we used our recently developed CRISPR-Cas9-mediated DUB KO kit [31, 32] to perform genome-scale screening for DUBs that affect PAX9 and MSX1 protein levels. The kit contains an entire set of individually lysed and aliquoted cell lysates from 50 USP-subfamily gene-knockout cell lines.

**Fig. 1:**
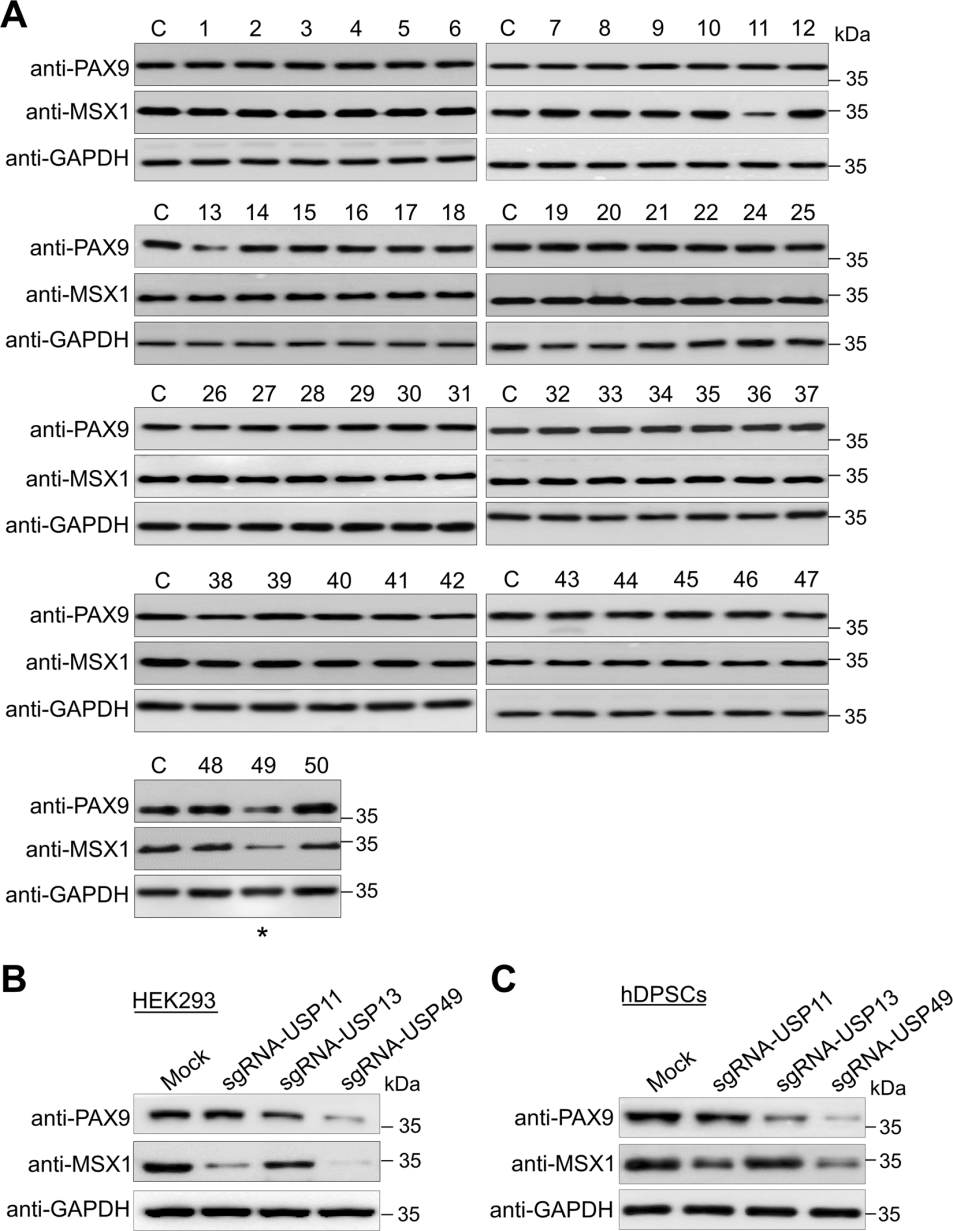
Genome-scale screening of the ubiquitin-specific protease subfamily for PAX9 and MSX1 proteins using CRISPR-Cas9-based DUB-knockout library kit. **A** Screening for USPs regulating PAX9 and MSX1 proteins using the CRISPR-Cas9-based DUB KO library kit. Equal protein concentrations from DUB KO HEK293 cell lysates were subjected to Western blotting to determine endogenous PAX9 and MSX1 protein levels. GAPDH was used as a loading control for each blot. Asterisk represents the selected DUB, which regulates both PAX9 and MSX1 proteins. The presented immunoblots are representative of three independent experiments (*n* = 3). **B** Effects of putative DUB candidates (USP11, USP13, and USP49) on PAX9 and MSX1 protein levels in HEK293 cells were estimated by Western blotting. The presented immunoblots are representative of three independent experiments (*n* = 3). **C** Effects of putative DUB candidates (USP11, USP13, and USP49) on PAX9 and MSX1 protein levels in hDPSCs were estimated by Western blotting. The presented immunoblots are representative of three independent experiments (*n* = 3).

Equal concentrations of all DUB KO cell lysates were analyzed by Western blotting to screen for DUB candidates that might regulate the endogenous levels of the PAX9 and MSX1 proteins. The loss-of-function of only a few putative DUB genes reduced PAX9 and MSX1 protein levels. Disruption of *USP13* and *USP49* genes reduced PAX9 protein, and *USP11* and *USP49* disruption reduced MSX1 protein levels (Fig. 1A). We cross-checked the efficiency of the sgRNA targeting these putative DUBs (*USP11*, *USP13*, and *USP49*) on PAX9 and MSX1 protein levels in HEK293 cells and hDPSCs. USP49 was the strongest candidate, producing a significant reduction in both PAX9 and MSX1 proteins (Fig. 1B, C, lane 4). Given that the interaction between Pax9 and Msx1 is critical during early tooth development, we studied a common candidate USP49 that regulates the levels of both PAX9 and MSX1 proteins.

### USP49 increases PAX9 and MSX1 protein levels

**Fig. 2:**
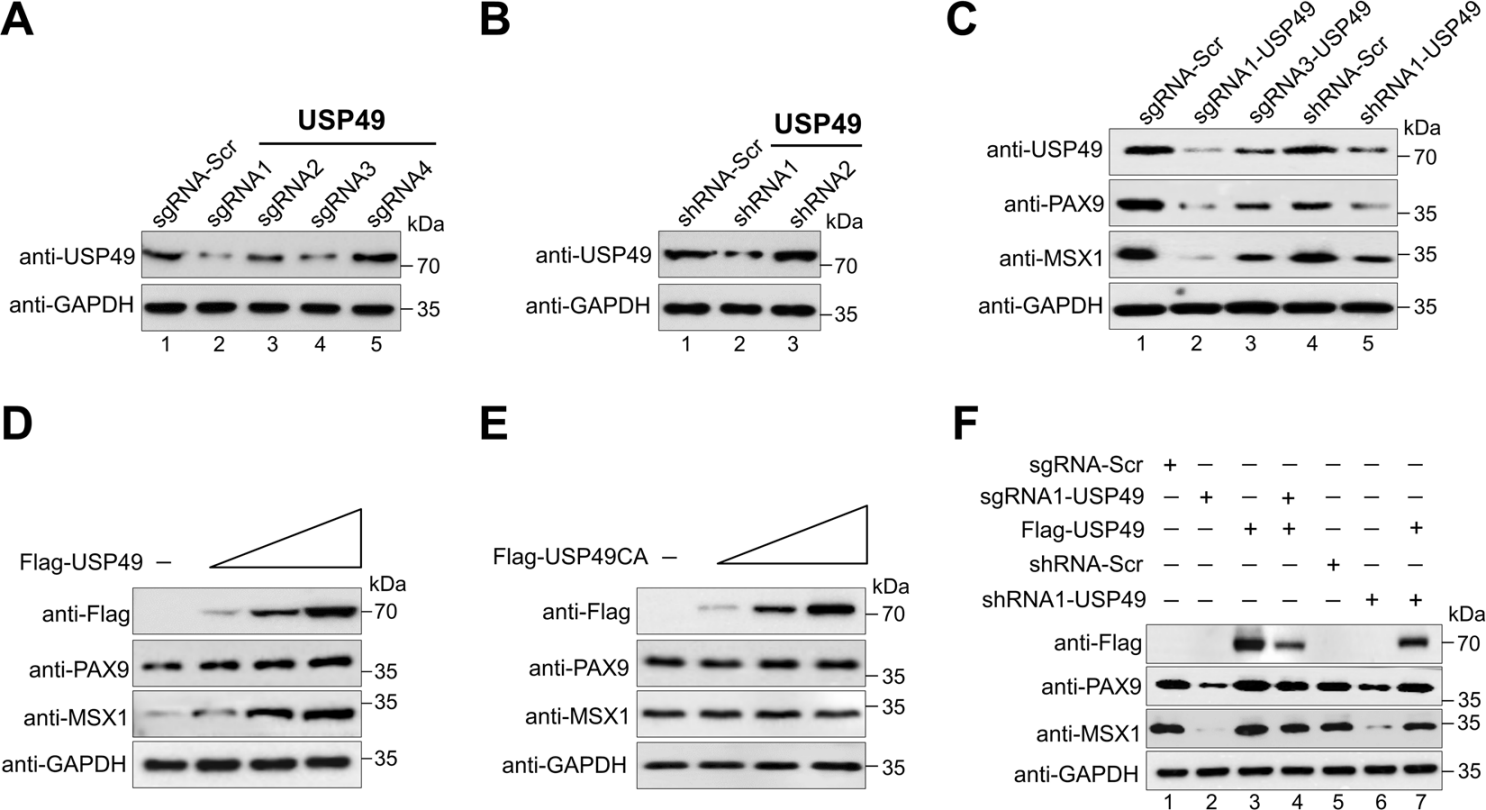
USP49 upregulates endogenous PAX9 and MSX1 proteins. **A** Validation of the efficiency of sgRNAs were carried out in hDPSCs by transiently transfecting sgRNA1 to sgRNA4 targeting *USP49*, and immunoblotting with the USP49 antibody. The presented immunoblots are representative of three independent experiments (*n* = 3). **B** Validation of the efficiency of shRNAs were carried out in hDPSCs by transiently transducing shRNA1 and shRNA2 targeting *USP49*, and immunoblotting with the USP49 antibody. The presented immunoblots are representative of three independent experiments (*n* = 3). **C** hDPSCs were transfected with two best sgRNAs (sgRNA1 and sgRNA3) and one shRNA (shRNA1) targeting *USP49* to check the endogenous protein levels of USP49, PAX9, and MSX1 using their respective endogenous antibodies. The presented immunoblots are representative of three independent experiments (*n* = 3). **D** hDPSCs were transfected with increasing concentrations of Flag-USP49 to check the endogenous USP49, PAX9, and MSX1 proteins. The presented immunoblots are representative of three independent experiments (*n* = 3). **E** hDPSCs were transfected with increasing concentrations of Flag-USP49CA to check the endogenous USP49, PAX9, and MSX1 proteins. The presented immunoblots are representative of three independent experiments (*n* = 3). **F** Reconstitution effect of USP49 on endogenous PAX9 or MSX1 in USP49-depleted cells. Protein expression was analyzed by Western blotting using the indicated antibodies. GAPDH was used as the loading control. The presented immunoblots are representative of three independent experiments (*n* = 3).

To understand the post-translational regulation of PAX9 and MSX1 by USP49, we applied both sgRNA and shRNA to silence *USP49* gene expression. The protein expression of USP49 was reduced by sgRNA1 more significantly than by the other sgRNAs (Fig. 2A, lane 2), which is in line with the high indel percentage observed in sgRNA1 (Supplementary Fig. S1A, B). Similarly, shRNA1 showed higher efficiency than shRNA2 (Fig. 2B, lane 2). Therefore, we used sgRNA1 (hereafter sgRNA) and shRNA1 (hereafter shRNA) targeting *USP49* for further functional studies.

Our analysis of endogenous USP49, PAX9, and MSX1 protein expression in different cell lines showed that all three proteins were highly expressed in hDPSCs (Supplementary Fig. S2). Therefore, we carried out further experiments in hDPSCs, which are relevant for odontogenic-related functional studies. Transient transfection of both sgRNA and shRNA targeting *USP49* significantly decreased the expression of endogenous PAX9 and MSX1 compared to the mock control (Fig. 2C, lanes 2 and 5). Similar results were observed when both sgRNA and shRNA targeting *USP49* were transfected into HEK293 cells with ectopic expression of Myc-PAX9 or Flag-MSX1 (Supplementary Fig. S3A, B). Furthermore, overexpression of USP49 in hDPSCs dose-dependently increased the expression of endogenous PAX9 and MSX1 proteins (Fig. 2D). However, overexpression of the catalytic mutant USP49 (USP49CA) did not have any effect on PAX9 and MSX1 proteins (Fig. 2E). Similarly, in HEK293 cells, we observed a USP49-mediated increase in the expression of Myc-PAX9 and Flag-MSX1 (Supplementary Fig. S3C, D), whereas USP49CA had no such effect (Supplementary Fig. S3E, F), suggesting that USP49 increases PAX9 and MSX1 protein levels.

Additionally, the reduced expression of endogenous PAX9 and MSX1 proteins caused by sgRNA or shRNA targeting *USP49* was rescued when hDPSCs were reconstituted with USP49 (Fig. 2F, lanes 4 and 7). Adding USP49 to HEK293 cells ectopically expressing PAX9 and MSX1 had a similar rescue effect (Supplementary Fig. S4A, B, lanes 7, 8). These results indicate that USP49 regulates PAX9 and MSX1 protein levels.

### USP49 interacts with PAX9 and MSX1

**Fig. 3:**
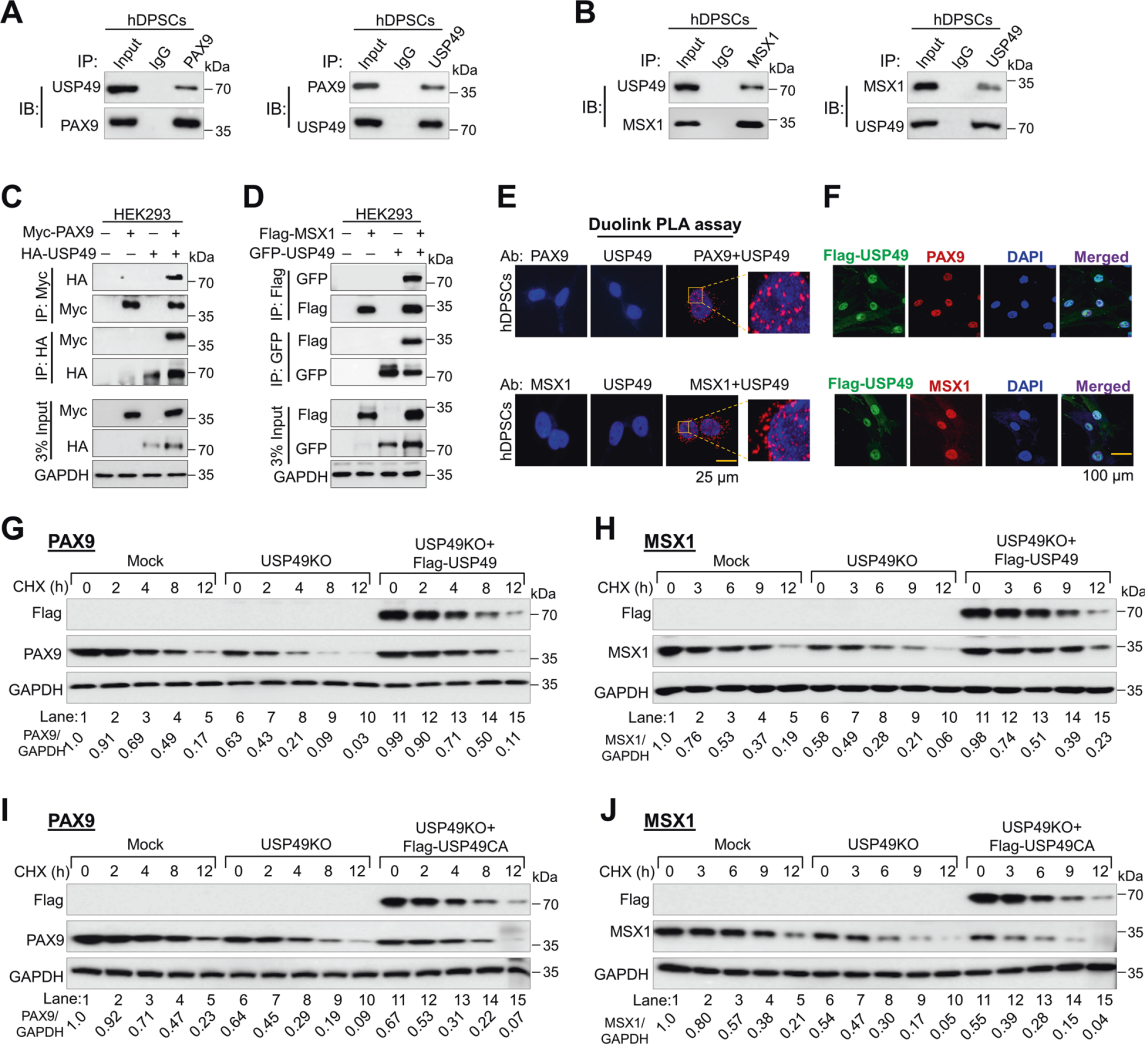
USP49 interacts with PAX9 and MSX1 to regulate their protein turnover. **A** The interactions between endogenous USP49 and PAX9 proteins were examined in hDPSCs. Cell lysates from hDPSCs were immunoprecipitated and immunoblotted with specific USP49 or PAX9 antibodies. The presented immunoblots are representative of three independent experiments (*n* = 3). **B** The interactions between endogenous USP49 and MSX1 proteins were examined in hDPSCs. Cell lysates from hDPSCs were immunoprecipitated and immunoblotted with specific USP49 or MSX1 antibodies. The presented immunoblots are representative of three independent experiments (*n* = 3). **C** Myc-PAX9 and HA-USP49 were co-transfected into HEK293 cells for immunoprecipitation. The presented immunoblots are representative of two independent experiments (*n* = 2). **D** Flag-MSX1 and GFP-USP49 were co-transfected into HEK293 cells. Samples were immunoprecipitated using their respective antibodies and immunoblotted using the indicated antibodies. GAPDH was used as the loading control. The presented immunoblots are representative of two independent experiments (*n* = 2). **E** hDPSCs were subjected to the Duo-link PLA assay to check the interaction between USP49 and PAX9; USP49 and MSX1 using their specific antibodies. Scale bar: 25 μm. The in situ USP49-PAX9 or USP49-MSX1 interaction (PLA dots) was observed when USP49 and PAX9 or MSX1 were immunostained together, but not when they were stained with individual antibodies. The presented microscopic images are representative of three independent experiments (*n* = 3). **F** Immunostaining with endogenous USP49 and PAX9 or MSX1 antibodies was performed in hDPSCs. As the specific antibodies against USP49, PAX9 or MSX1 were derived from the same species, we transduced hDPSCs with Flag-USP49 and performed staining with Flag- and PAX9- or MSX1-specific antibodies. DAPI was used for nuclear staining. Scale bar: 100 µm. The presented microscopic images are representative of three independent experiments (*n* = 3). **G** The half-life of PAX9 was determined in the Mock, USP49 KO, and USP49 KO cells reconstituted with USP49 after treatment with CHX for the indicated time points. Densitometric analysis of PAX9 expression (normalized to GAPDH) is reported under the blot and represents the mean of three independent experiments (*n* = 3). **H** The half-life of MSX1 was determined in the Mock, USP49 KO, and USP49 KO cells reconstituted with USP49 after treatment with CHX for the indicated time points. Densitometric analysis of MSX1 expression (normalized to GAPDH) is reported under the blot and represents the mean of three independent experiments (*n* = 3). **I** The half-life of PAX9 was determined in the Mock, USP49 KO, and USP49 KO cells reconstituted with USP49CA after treatment with CHX for the indicated time points. Densitometric analysis of PAX9 expression (normalized to GAPDH) is reported under the blot and represents the mean of three independent experiments (n = 3). **J** The half-life of MSX1 was determined in the Mock, USP49 KO, and USP49 KO cells reconstituted with USP49CA after treatment with CHX for the indicated time points. Protein band intensities were estimated using the ImageJ software with reference to the GAPDH control. The band intensity for PAX9/GAPDH or MSX1/GAPDH was represented below the blots. Densitometric analysis of MSX1 expression (normalized to GAPDH) is reported under the blot and represents the mean of three independent experiments (*n* = 3).

To investigate the molecular mechanisms underlying the critical functions and interactions between USP49 and PAX9 or MSX1, we analyzed whether they physically associate in vivo. Endogenous USP49 stably interacted with both endogenous PAX9 (Fig. 3A) and MSX1 (Fig. 3B) proteins. Furthermore, exogenous HA-USP49 co-precipitated Myc-PAX9 and vice versa (Fig. 3C). Similarly, GFP-USP49 co-precipitated Flag-MSX1 and vice versa (Fig. 3D), indicating that USP49 interacts with PAX9 and MSX1 proteins both endogenously and exogenously. Additionally, we demonstrated USP49-PAX9 and USP49-MSX1 interactions using the Duolink PLA assay (Fig. 3E). Immunostaining with specific antibodies showed USP49, PAX9, and MSX1 expression in the nuclei of hDPSCs (Fig. 3F).

### USP49 extends the half-lives of PAX9 and MSX1

We analyzed the half-lives of PAX9 and MSX1 proteins in the presence or absence of USP49. The half-life of PAX9 was ~8 h (Supplementary Fig. S5A), and the half-life of MSX1 was ~6 h (Supplementary Fig. S5B). Overexpression of USP49 in hDPSCs extended the half-lives of PAX9 (Supplementary Fig. S5C, lanes 6–10) and MSX1 (Supplementary Fig. S5D, lanes 6–10) compared with the mock control (Supplementary Fig. S5C, D, lanes 1–5). In contrast, USP49-depletion in hDPSCs reduced the half-lives of PAX9 (Fig. 3G, lanes 6–10) and MSX1 (Fig. 3H, lanes 6–10), which was rescued when USP49 was reconstituted in USP49-depleted hDPSCs (Fig. 3G, H, lanes 11–15). Reconstitution of the USP49 catalytic mutant did not rescue the reduced half-lives of PAX9 (Fig. 3I, lanes 11–15) and MSX1 (Fig. 3J, lanes 11–15).

Additionally, we observed that exogenous PAX9 (Supplementary Fig. S6A) and MSX1 (Supplementary Fig. S6B) had extended half-lives in the presence of USP49, whereas the USP49 catalytic mutant showed no effect on PAX9 (Supplementary Fig. S6C) or MSX1 protein levels (Supplementary Fig. S6D). Thus, the deubiquitinating activity of USP49 regulates the protein turnover of PAX9 and MSX1.

### Polyubiquitination of PAX9 and MSX1 proteins

First, we examined the effect of *USP49* on *PAX9* and *MSX1* mRNA levels in hDPSCs. RT-PCR showed no significant changes in *PAX9* (Supplementary Fig. S7A) and *MSX1* (Supplementary Fig. S7B) at the mRNA level, indicating that *USP49* does not have any role in the transcriptional regulation of *PAX9* or *MSX1*.

**Fig. 4:**
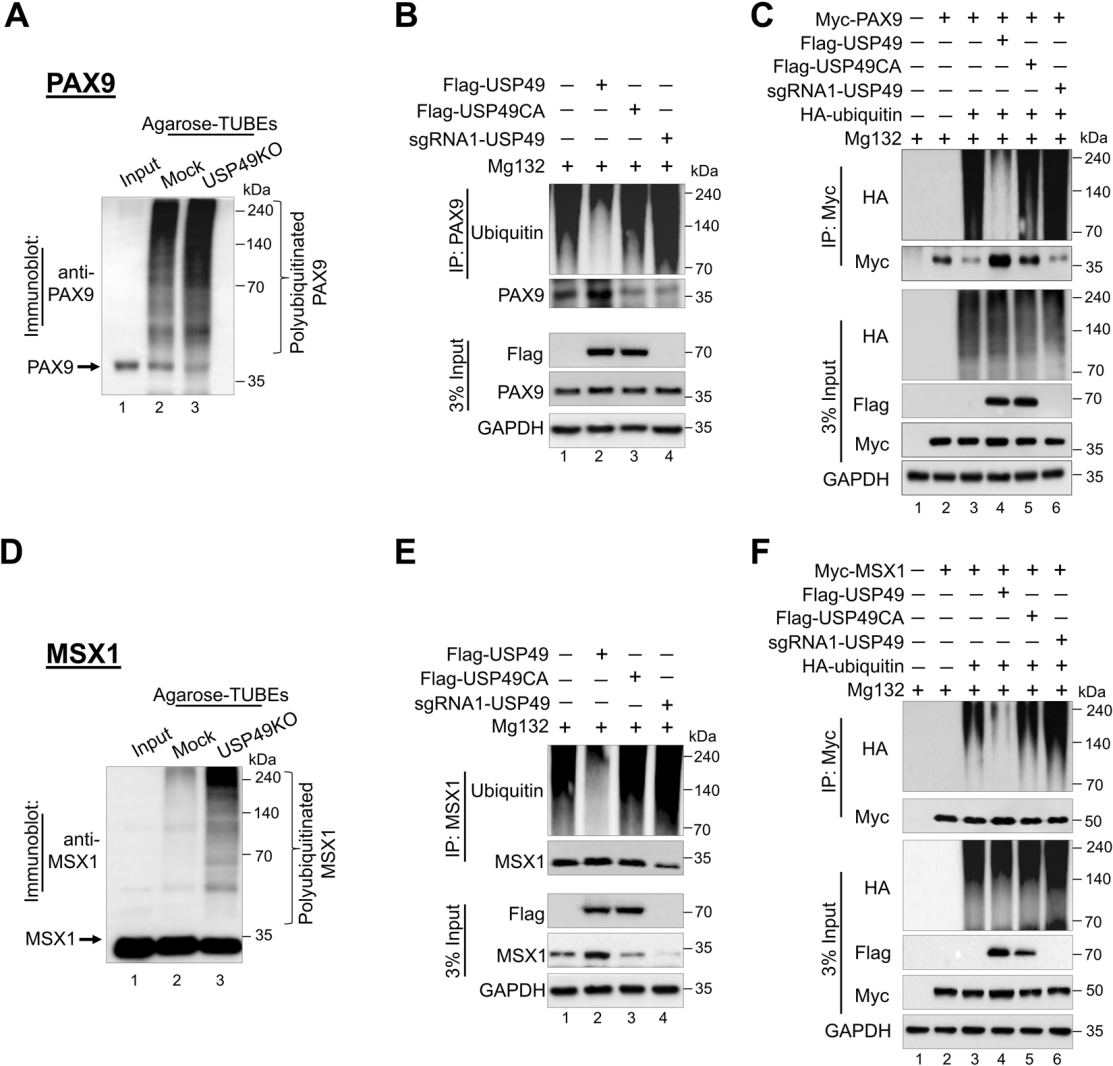
USP49 deubiquitinates PAX9 and MSX1. **A** TUBEs assay for the ubiquitination of PAX9 was conducted in mock-hDPSCs and USP49 KO-hDPSCs using TUBEs-agarose beads. Cell lysates were immunoprecipitated with TUBEs antibodies, followed by immunoblotting with the specific anti-PAX9 antibody. Lane 1, input; Lane 2, mock-hDPSCs; and Lane 3, USP49 KO-hDPSCs. The presented immunoblots are representative of two independent experiments (*n* = 2). **B** The ubiquitination and deubiquitination of endogenous PAX9 were analyzed by transfecting hDPSCs with Flag-USP49, Flag-USP49CA, and sgRNA targeting *USP49* followed by immunoprecipitation with an anti-PAX9 antibody and immunoblotting with an anti-ubiquitin antibody. The cells were then treated with MG132 for 6 h prior to harvest for all these experiments. The presented immunoblots are representative of two independent experiments (*n* = 2). **C** HEK293 cells were transfected with Myc-PAX9, HA-ubiquitin, Flag-USP49, Flag-USP49CA, and sgRNA targeting *USP49*. The deubiquitination of PAX9 was confirmed by co-immunoprecipitation with the anti-Myc antibody and immunoblotting with the anti-HA antibody. The presented immunoblots are representative of two independent experiments (*n* = 2). **D** The TUBEs assay for the ubiquitination of MSX1 proteins was conducted in mock-hDPSCs and USP49 KO-hDPSCs using TUBEs-agarose beads. Cell lysates were immunoprecipitated with the TUBEs antibody followed by immunoblotting with the specific anti-MSX1 antibody. Lane 1, input; Lane 2, mock-hDPSCs; Lane 3, USP49 KO-hDPSCs. The presented immunoblots are representative of two independent experiments (*n* = 2). **E** The ubiquitination and deubiquitination of endogenous MSX1 were analyzed by transfecting hDPSCs with Flag-USP49, Flag-USP49CA, and sgRNA targeting *USP49*, followed by immunoprecipitation with an anti-MSX1 antibody and immunoblotting with an anti-ubiquitin antibody. The cells were then treated with MG132 for 6 h prior to harvest for all experiments. The presented immunoblots are representative of two independent experiments (*n* = 2). **F** HEK293 cells were transfected with Myc-MSX1 and HA-ubiquitin, Flag-USP49, Flag-USP49CA, and sgRNA targeting *USP49*. The deubiquitination of MSX1 was confirmed by co-immunoprecipitation with an anti-Myc antibody and immunoblotting with an anti-HA antibody. The presented immunoblots are representative of two independent experiments (*n* = 2).

Next, we checked whether PAX9 and MSX1 undergo degradation via the 26 S proteasomal pathway. Increasing concentrations of the proteasomal inhibitor MG132 produced an increase in endogenous PAX9 and MSX1 proteins (Supplementary Fig. S8A, B). Moreover, the downregulation effect of sgRNA targeting *USP49* on PAX9 and MSX1 was rescued by MG132 treatment (Supplementary Fig. S8C, D). To date, no ubiquitination studies have been performed on the PAX9 protein; therefore, we assessed its ubiquitination status in HEK293 cells. The ubiquitin smear was observed only in the transfected PAX9 samples, along with the ubiquitin construct (Supplementary Fig. S8E, lane 4). Furthermore, the ubiquitination of endogenous PAX9 in the TUBEs assay showed an increased level of PAX9 ubiquitination in USP49 KO-hDPSCs (Fig. 4A, lane 3) compared with the mock-hDPSCs (Fig. 4A, lane 2).

Similarly, an immunoprecipitation analysis of HEK293 cells co-transfected with Flag-MSX1 and HA-ubiquitin showed polyubiquitination of the MSX1 protein (Supplementary Fig. S8F, lane 4), and the TUBEs assay revealed that the loss of USP49 produced a higher ubiquitination status for MSX1 (Fig. 4D, lane 3) than mock-hDPSCs (Fig. 4D, lane 2). Thus, PAX9 and MSX1 ubiquitination was elevated in USP49 KO-hDPSCs, indicating that the loss of USP49 promotes PAX9 and MSX1 protein degradation.

### USP49 deubiquitinates PAX9 and MSX1 proteins

Next, we analyzed the deubiquitinating activity of USP49 on endogenous and exogenous PAX9 and MSX1. USP49 significantly decreased the polyubiquitination of both endogenous PAX9 (Fig. 4B, lane 2) and MSX1 (Fig. 4E, lane 2), as well as exogenous PAX9 (Fig. 4C, lane 4) and MSX1 (Fig. 4F, lane 4). However, no deubiquitinating activity was observed for PAX9 and MSX1 proteins in the presence of USP49CA (Fig. 4B, lane 3; 4E, lane 3; Fig. 4C, lane 5; Fig. 4F, lane 5), upon knockdown of USP49 (Fig. 4B, lane 4; 4E, lane 4; Fig. 4C, lane 6; Fig. 4F, lane 6), USP44, a paralog of USP49 [38] (Supplementary Fig. S9A, B, lane 6), indicating that USP49 deubiquitinates PAX9 and MSX1.

### USP49 promotes the odontogenic differentiation of hDPSCs by stabilizing PAX9 and MSX1

**Fig. 5:**
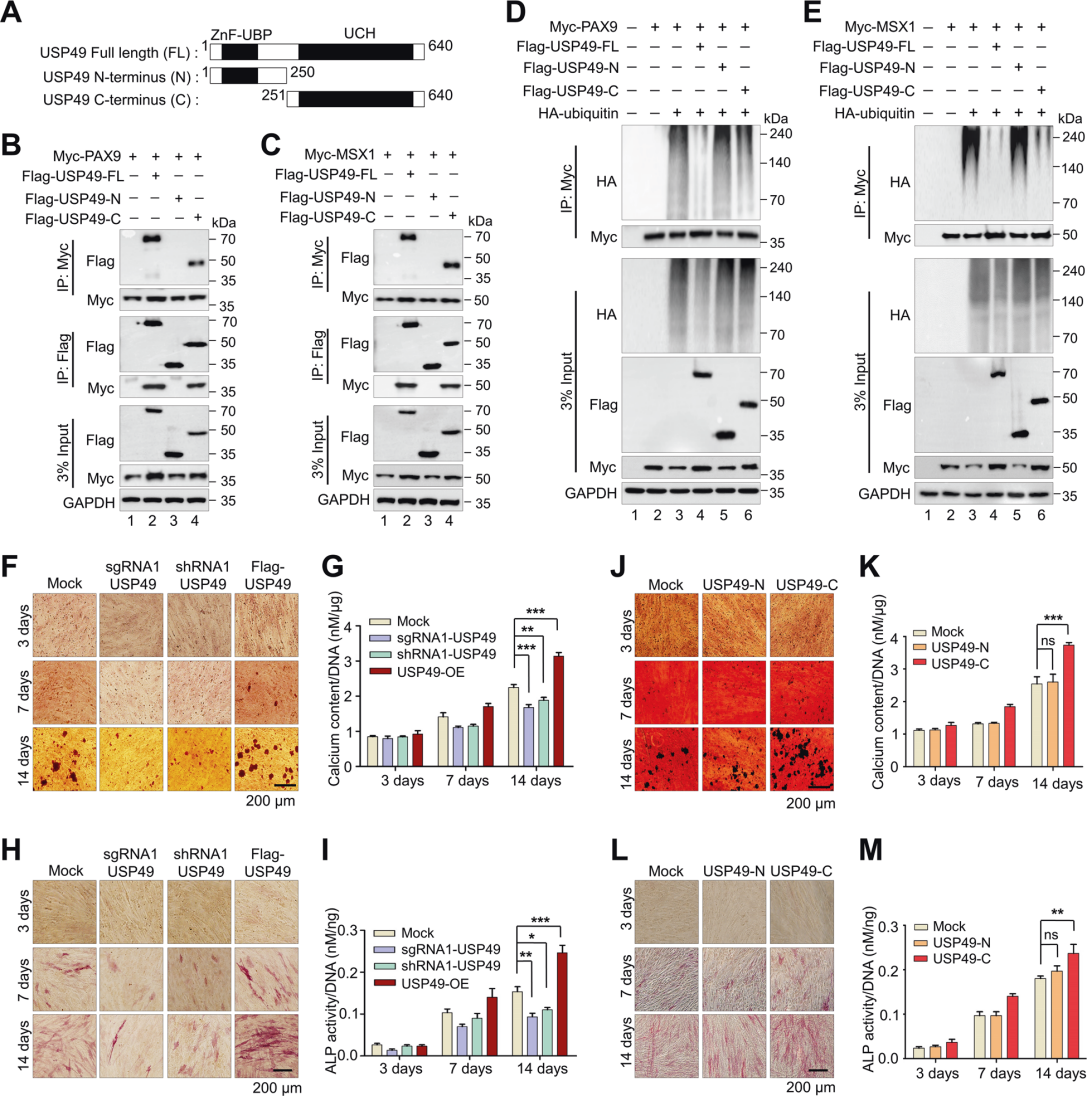
Odontogenic differentiation is regulated by USP49. **A** Schematic representation of full length USP49 (1-640 aa) encoding ZnF-UBP and UCH domains (represented as USP49-FL), N-terminus USP49 (1-250 aa) encoding ZnF-UBP domain (represented as USP49-N), and C-terminus USP49 (251-640 aa) encoding UCH domain (represented as USP49-C). **B** Interactions between USP49-FL and PAX9; USP49-N and PAX9; USP49-C and PAX9 by IP and immunoblotting with the indicated antibodies. The presented immunoblots are representative of three independent experiments (*n* = 3). **C** Interaction between USP49-FL and MSX1; USP49-N and MSX1; USP49-C and MSX1 by IP followed by immunoblotting with the indicated antibodies. The presented immunoblots are representative of three independent experiments (*n* = 3). **D** The deubiquitination of PAX9 was analyzed by transfecting HEK293 cells with USP49-FL, USP49-N, and USP49-C, and determined by IP followed by immunoblotting with the indicated antibodies. The presented immunoblots are representative of three independent experiments (*n* = 3). **E** The deubiquitination of MSX1 was analyzed by transfecting HEK293 cells with USP49-FL, USP49-N, and USP49-C, and determined by IP followed by immunoblotting with the indicated antibodies. The presented immunoblots are representative of three independent experiments (*n* = 3). **F** Representative images of alizarin red staining performed in hDPSCs (Mock, Flag-USP49, sgRNA1 targeting *USP49* and shRNA1 targeting *USP49*) group at the indicated time points (days 3, 7, and 14). Scale bar: 200 µm. The presented microscopic images are representative of three independent experiments (*n* = 3). **G** To quantify the alizarin red staining, the calcium content was quantified using the CPC method and normalized to the DNA content per well as measured on days 3, 7, and 14. The calcium content was calculated based on the mean of three technical replicates for each individual experiment. The data presented here represent the mean ± standard deviation (SD) of three independent experiments (*n* = 3) (**P < 0.001 and ***P < 0.0001) by analysis of variance (ANOVA) followed by Tukey’s post hoc test. (H) Representative images of hDPSCs (Mock, Flag-USP49, sgRNA1 targeting *USP49* and shRNA1 targeting *USP49*) group stained with alkaline phosphatase (ALP) at the time points indicated (days 3, 7, and 14). Scale bar: 200 µm. The presented microscopic images are representative of three independent experiments (*n* = 3). **I** ALP activity was quantified and normalized to the DNA content obtained from each individual dataset on days 3, 7 and 14. The ALP activity was calculated based on the mean of three technical replicates for each individual experiment. The data presented here are the mean ± SD of three independent experiments (*n* = 3) (*P < 0.05, **P < 0.001 and ***P < 0.0001), by ANOVA followed by Tukey’s post hoc test. **J** Representative images from ALZ staining in hDPSCs (Mock, USP49-N, USP49-C) on days 3, 7, and 14. Scale bar: 200 µm. The presented microscopic images are representative of three independent experiments (*n* = 3). **K** To quantify the ALZ staining, the calcium content was quantified using the CPC method and normalized to the DNA content per well as measured on days 3, 7, and 14. The calcium content was calculated based on the mean of three technical replicates for each individual experiment. The data presented here represent the mean ± SD of three independent experiments (*n* = 3) (ns = non-significant, **P < 0.001) by ANOVA followed by Tukey’s post hoc test. **L** Representative images of hDPSCs (Mock, USP49-N, USP49-C) group stained with ALP on days 3, 7, and 14. Scale bar: 200 µm. The presented microscopic images are representative of three independent experiments (*n* = 3). **M** ALP activity was quantified and normalized to the DNA content obtained from each individual dataset on days 3, 7 and 14. The ALP activity was calculated based on the mean of three technical replicates for each individual experiment. The data presented here represent the mean ± SD of three independent experiments (*n* = 3) (ns = non-significant, **P < 0.001) by ANOVA followed by Tukey’s post hoc test.

To further corroborate our finding that USP49 interacts with PAX9 and MSX1 proteins, we sought to identify which region of USP49 interacts with PAX9 and MSX1. To this end, we generated two truncated variants of USP49 (N-terminus USP49 (1-250 aa) variant encoding the ZnF-UBP domain and C-terminus USP49 (251-640 aa) variant encoding the UCH domain) (Fig. 5A) and performed co-immunoprecipitation with PAX9 and MSX1. The N-terminus USP49 variant did not interact with either PAX9 or MSX1 (Fig. 5B, C; lane 3), whereas the C-terminus USP49 variant interacted with PAX9 and MSX1 (Fig. 5B, C; lane 4), suggesting that the UCH domain of USP49 is required for its interaction with PAX9 and MSX1. Our finding is in line with a previous report showing that the UCH domain in the C-terminus of USP49 is necessary to interact with and deubiquitination of its substrate [39].

Next, we investigated the regulatory role of N- and C-terminus USP49 on PAX9 and MSX1 proteins. C-terminus USP49 with the UCH domain upregulates (Supplementary Fig. S10B, D), deubiquitinates (Fig. 5D, E; lane 6), and extends the half-lives (Supplementary Fig. S10E, F; lanes 11–15) of PAX9 and MSX1 proteins, whereas the N-terminus interaction-defective USP49 showed no such effects (Fig. 5D, E, Supplementary Fig. S10A, C, E, F). Further, we examined the odontogenic differentiation of hDPSCs in the presence or absence of USP49 at various time points using ALZ and ALP assays. Upon USP49 overexpression, the ALZ assay showed a mineralized, nodule-like aggregation of cells during the first week that increased significantly during the second week (Fig. 5F). Similarly, C-terminus USP49 with a UCH domain also showed increased mineralization and nodule-like aggregation (Fig. 5J). In contrast, extracellular matrix mineralization was markedly reduced in USP49 KO-hDPSCs (Fig. 5F). The colorimetric analysis indicated high calcium deposition in USP49 and C-terminus USP49 when compared with USP49 KO-hDPSCs (Fig. 5G, K). Similarly, ALP staining and activity were more intense in USP49 and C-terminus USP49 than in USP49 KO-hDPSCs (Fig. 5H, I, L, M). Importantly, the extent of extracellular matrix mineralization and calcium deposition in the N-terminus interaction-defective USP49 was similar to that in the mock control (Fig. 5J, K). Likewise, N-terminus USP49 showed similar ALP staining and intracellular ALP activity as mock-control (Fig. 5L, M), indicating that the interaction between USP49 and PAX9 or MSX1 is essential for odontogenesis.

### Generation of single cell-derived USP49-knockout clones in human pluripotent stem cells

**Fig. 6:**
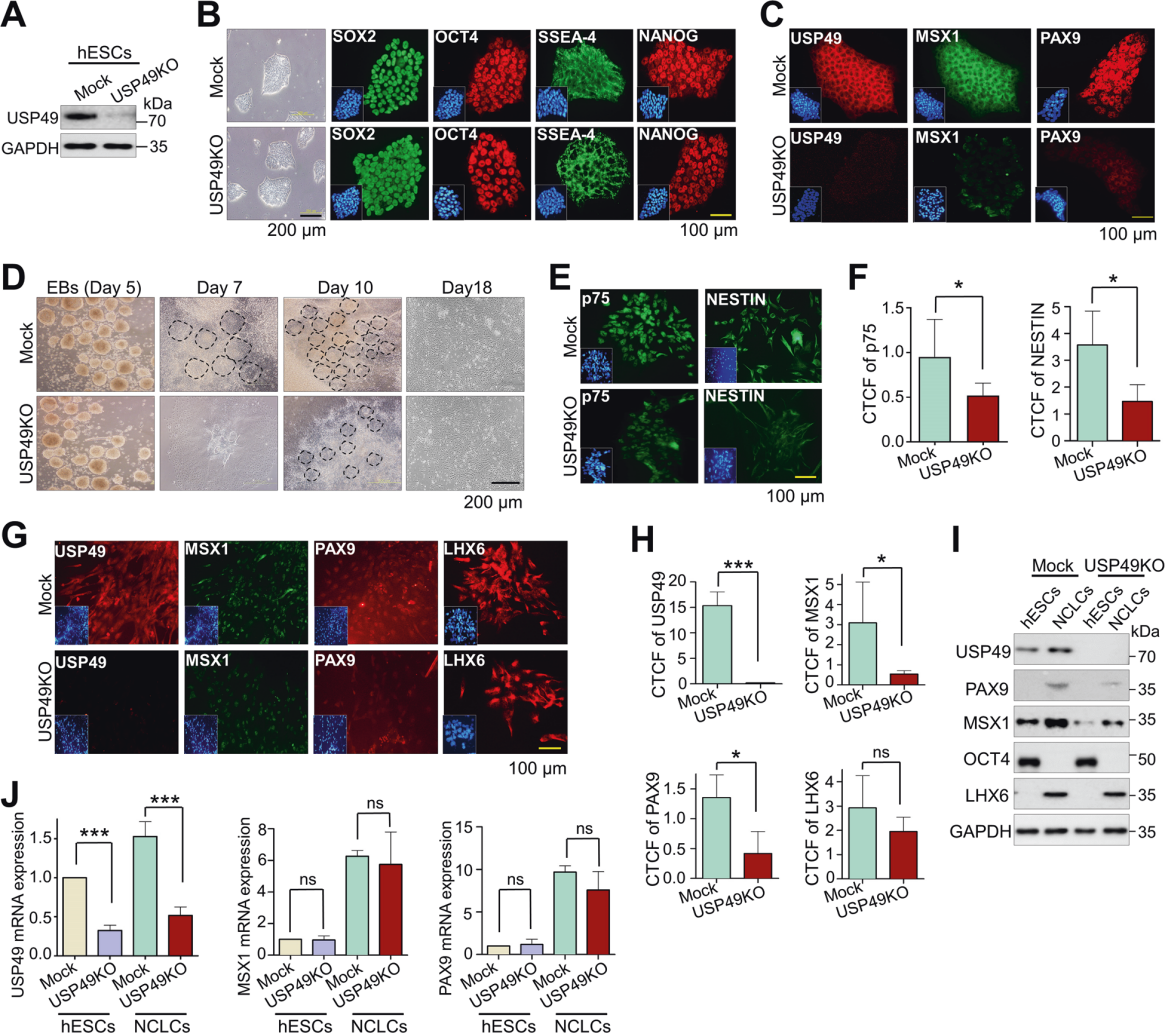
Loss of USP49 in hESCs delays differentiation by downregulating PAX9 and MSX1. **A** USP49 KO in hESCs was confirmed by analyzing endogenous USP49 protein expression through Western blotting. GAPDH was used as the internal loading control. The presented immunoblots are representative of three independent experiments (*n* = 3). **B** Expression levels of endogenous pluripotency markers (SOX2, OCT4, SSEA-4 and NANOG) were confirmed by immunofluorescence staining in mock and USP49 KO-hESCs. Scale bar: 100 µm. The presented microscopic images are representative of three independent experiments (*n* = 3). **C** Endogenous expression levels of USP49, MSX1 and PAX9 were confirmed by immunofluorescence staining in mock and USP49 KO-hESCs. Scale bar: 100 µm. The presented microscopic images are representative of three independent experiments (*n* = 3). **D** Embryoid bodies (EBs) derived from mock and USP49 KO-hESCs were differentiated into NCLCs. Cell morphology during differentiation was analyzed by bright field microscopy at the indicated time points. Dotted outline indicates neural rosette formation during differentiation. Scale bar: 200 µm. The presented microscopic images are representative of three independent experiments (*n* = 3). **E** Immunofluorescence staining was performed on mock and USP49 KO-derived NCLCs on day 18 to analyze the NCLC markers (p75 and NESTIN). Scale bar: 100 µm. The presented microscopic images are representative of three independent experiments (*n* = 3). **F** Corrected total cell fluorescence (CTCF) intensity of p75 and NESTIN in mock and USP49 KO-derived NCLCs was quantified by the ImageJ software. CTCF intensities were calculated for each individual experiment based on the mean of three technical replicates The data presented here represent the mean ± SD of three independent experiments (*n* = 3), (*P < 0.05) by Student’s *t* test. **G** Effects of *USP49* gene disruption on the endogenous expression levels of dental mesenchymal markers (MSX1, PAX9, and LHX6) were analyzed by immunofluorescence staining on day 18 of NCLC differentiation. Scale bar: 100 µm. The presented microscopic images are representative of three independent experiments (*n* = 3). **H** Corrected total cell fluorescence (CTCF) intensity of USP49, MSX1, PAX9, and LHX6 in mock and USP49 KO-derived NCLCs was quantified by the ImageJ software. CTCF intensities were calculated for each individual experiment based on the mean of three technical replicates. The data presented here represent the mean ± SD of three independent experiments (*n* = 3), (*P < 0.05, ***P < 0.0001 and non-significant (ns), by Student’s *t* test). **I** Effects of USP49 KO on the endogenous expression levels of dental mesenchymal markers (MSX1, PAX9, and LHX6) and a pluripotency marker (OCT4) in undifferentiated and differentiated cells were analyzed by immunoblotting with specific antibodies. GAPDH was used as the internal loading control. The presented immunoblots are representative of two independent experiments (*n* = 2). **J** Effects of USP49 KO on the mRNA expression levels of *USP49*, *MSX1*, and *PAX9* in undifferentiated and differentiated cell lines were analyzed by qRT-PCR with specific primers. Relative mRNA expression levels are shown after normalization to GAPDH mRNA expression. The fold changes of mRNA levels were calculated for each individual experiment based on the mean of three technical replicates. The data presented here represent the mean ± SD of three independent experiments (*n* = 3). For *USP49* mRNA expression: ***P < 0.0001 for mock-hESCs vs. USP49 KO-hESCs, ***P < 0.0001 for mock-NCLCs vs. USP49 KO-NCLCs. For *MSX1* mRNA expression: non-significant (ns) for mock-hESCs vs. USP49 KO-hESCs, non-significant (ns) for mock-NCLCs vs. USP49 KO-NCLCs. For *PAX9* mRNA expression: non-significant (ns) for mock-hESCs vs. USP49 KO-hESCs, non-significant (ns) for mock-NCLCs vs. USP49 KO-NCLCs. Statistical significance was analyzed by ANOVA followed by Tukey’s post hoc test.

Next, we examined the effect of *USP49* KO on the differentiation of hPSCs (hESCs and hiPSCs) into NCLCs. To this end, we generated USP49 KO-clones in hESCs using the CRISPR-Cas9 system. The transfected hESCs were diluted and seeded into 96-well plates for single-cell clonal selection and screened for *USP49* gene disruption using the T7E1 assay (Supplementary Fig. S11A). The T7E1-positive clones were subjected to Sanger sequencing, which showed a large deletion within the exon 1 region of USP49 KO-clone#22 (Supplementary Fig. S11B). The expression of USP49 at both mRNA (Supplementary Fig. S11C) and protein levels (Fig. 6A) was significantly reduced in USP49 KO-clone#22. Therefore, we used USP49 KO-clone#22 (hereafter USP49 KO) for further differentiation studies. Next, we characterized the pluripotency of USP49 KO-hESCs using RT-PCR, Western blotting, and immunostaining. USP49 KO-clone did not affect the stem cell morphology (Fig. 6B, left panel) and expression of the pluripotency marker, OCT4 at the mRNA (Supplementary Fig. S11D) or protein (Fig. 6B) levels. Moreover, immunostaining showed a complete reduction in USP49 expression in the USP49 KO-clone, which reduced PAX9 and MSX1 expression (Fig. 6C).

Additionally, we generated USP49 KO-clones in hiPSCs (Supplementary Fig. S12A) showing an out-of-frame mutation in USP49 KO-clone#28 (Supplementary Fig. S12B). USP49 KO-clone#28 showed a significant reduction in USP49 at both the protein and mRNA levels compared with mock-hiPSCs (Supplementary Fig. S12C, D) without affecting the pluripotency marker OCT4 (Supplementary Fig. S12E, F). Therefore, we used USP49 KO-clone#28 for further differentiation studies. USP49 expression was significantly reduced in USP49 KO-clone#28, resulting in reduced PAX9 and MSX1 expression in USP49 KO-clone#28 compared with the mock control (Supplementary Fig. S12G).

### Loss of USP49 destabilizes PAX9 and MSX1 and retards the differentiation of hPSCs

Next, we analyzed how the loss-of-function of *USP49* affected the differentiation of hESCs into NCLCs in vitro. Embryoid bodies derived from mock control and USP49 KO-hESCs were subjected to neural differentiation. Homogenous differentiation of mock-hESCs into NCLCs produced extensive, microscopically visible colonies containing clusters of neural rosettes on day 7. USP49 KO-hESCs showed delayed neural rosette formation (days 10–11) (Fig. 6D). We then allowed neural migratory cells with a stellate morphology to differentiate into neural crest cells. Immunostaining of the differentiated NCLCs on day 18 showed that the USP49 KO-clones had lower expression of neural crest cell markers such as p75 and NESTIN than the mock controls (Fig. 6E, F). Moreover, immunostaining showed lower levels of dental mesenchymal cell markers (MSX1, PAX9, and LHX6) in USP49-depleted-NCLCs, indicating delayed differentiation into NCLCs (Fig. 6G, H). Western blot analysis also showed a reduction in MSX1 and PAX9 protein expression in USP49-depleted-NCLCs compared with mock-NCLCs (Fig. 6I). Interestingly, we found no significant difference in the mRNA expression of *MSX1* or *PAX9* between the mock-NCLCs and USP49-depleted-NCLCs (Fig. 6J), confirming that USP49 post-translationally regulates MSX1 and PAX9 during hESC differentiation. Furthermore, RT-PCR analysis of neural crest cell-specific transcriptional factors (*NOTCH1*, *NOTCH2*, *SLUG)* showed that the loss of USP49 hindered their mRNA expression (Supplementary Fig. S13B).

In hiPSCs, loss-of-function of the *USP49* gene in hiPSCs also caused delayed differentiation into NCLCs (Supplementary Fig. S14A). Immunostaining of p75, NESTIN, MSX1, PAX9, and LHX6 was reduced in USP49-depleted-NCLCs (Supplementary Fig. S14B, C). Western blotting also showed reduced expression of USP49, PAX9, and MSX1 (Supplementary Fig. S14D). Overall, our results suggest that USP49 depletion delays the differentiation of hPSCs into NCLCs.

### Depletion of USP49 impairs normal odontogenesis

**Fig. 7:**
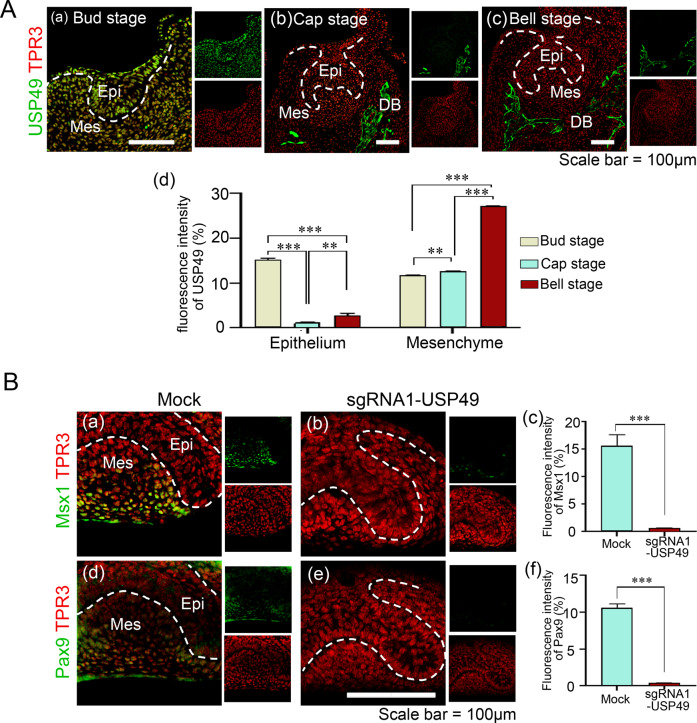
USP49 localization and its effects on the expression profiles of Pax9 and Msx1 during different stages of tooth development. **A** USP49 expression patterns during tooth development at the bud, cap, and bell stages. USP49 was expressed in the epithelium and mesenchyme (a) at the bud stage and in the developing bone surrounding the developing tooth germs at both the (b) cap and (c) bell stages. (d) Quantification of the fluorescence intensity of USP49 in developing dental epithelium and dental mesenchyme of tooth germs. Scale bar: 100 µm. Six biological replicates at each stage (*n* = 6) (fluorescence intensities were quantified from three biological replicates with randomly selected sections at each stage). The data presented here represent the mean ± SD of three independent experiments (**P < 0.001 and ***P < 0.0001). Statistical significance was analyzed by ANOVA followed by Tukey’s post hoc test. **B** Expression levels of Msx1 and Pax9 in tooth germs (a, d) mock-group and (b, e) sgRNA1-USP49-depletion group after 2 days of in vitro culture. The fluorescence intensities of (c) Msx1 and (f) Pax9 were compared between the mock-group and USP49-depletion group (*n* = 3). Scale bar = 100 µm. Epi = Epithelium, Mes = Mesenchyme, DB = Developing Bone. Six biological replicates per group (*n* = 6) (fluorescence intensities were quantified from three biological replicates with randomly selected sections at each stage). The data presented here represent the mean ± SD of three independent experiments (***P < 0.0001) by Student’s *t* test.

We further investigated the role of USP49 in tooth development in mice. USP49 localization was assessed at various stages of embryonic tooth development. USP49 is expressed in the dental epithelium and mesenchyme during the bud stage (Fig. 7A (a)). However, during the cap and bell stages, the USP49 protein was limited to the presumptive bone development in the dental mesenchyme surrounding the developing tooth germ, but not in the dental epithelium (Fig. 7A (b, c)). The intensity of USP49 expression was high in the dental epithelium at the bud stage, but in the dental mesenchyme, USP49 expression was high at the bell stage (Fig. 7A (d)). USP49, which was localized in the dental epithelium at the bud stage, was translocated to the developing bone region during the later stages of embryonic tooth development.

Next, we investigated the function of USP49 and its effects on PAX9 and MSX1 during tooth development. The tooth germ from the bud stage of mouse embryonic day 12.5 (E12.5) was surgically dissected. The samples were electroporated with an empty vector (mock control) or sgRNA targeting *USP49* along with Cas9, and then cultured under optimum in vitro organ culture conditions (Supplementary Fig. S15). We observed mesenchymal condensation during tooth formation using H&E staining. The USP49-knockdown tooth germs were smaller than the mock controls (Supplementary Fig. S16A), indicating that USP49 depletion affects tooth formation. Immunostaining showed MSX1- and PAX9-positive cells in the dental mesenchyme of the mock control group (Fig. 7B (a, d)), which is in line with previous reports [14, 40], but USP49-depletion (Fig. 7B (b, e)) markedly diminished the expression of both PAX9 and MSX1 in the dental mesenchyme (Fig. 7B (c, f)).

**Fig. 8:**
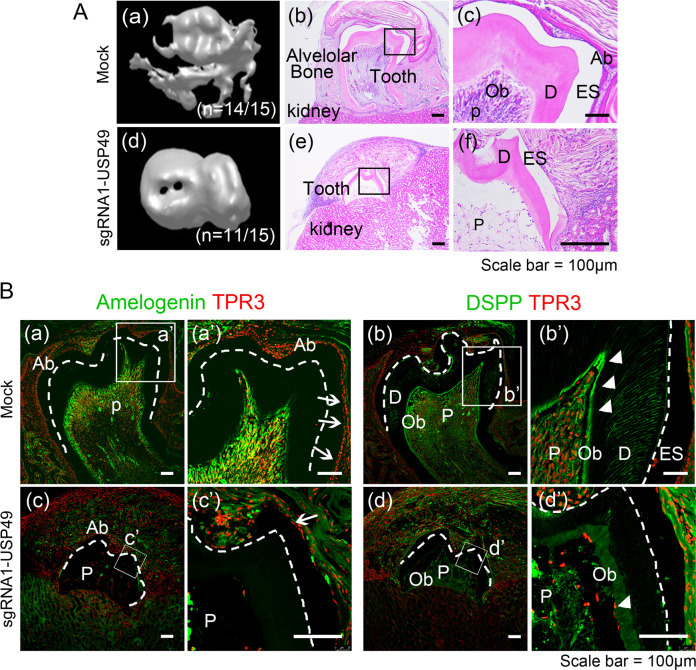
Depletion of USP49 impairs the tooth development. **A** Calcified teeth obtained after kidney capsule transplantation for 4 weeks. Three-dimensional (3D) reconstruction images from micro-computed tomography of calcified teeth in the (a) mock-group and (d) USP49-depletion group. H&E staining images from the (b, c) mock-group and (e, f) USP49-depletion group. Scale bar: 100 µm. (a (a, b)) In mock-group, the tooth is well calcified and surrounded by bone. (c) Pulp, odontoblasts, dentin, and ameloblasts were observed in the mock group. (a (d, e)) In the USP49-depletion group, the tooth is not fully calcified and not surrounded by bone. (f) Tooth showed defects in the pulp, dentin formation with few odontoblasts, and enamel formation with few ameloblasts. Fifteen biological replicates per group (*n* = 15) (H&E staining was performed from six biological replicates with randomly selected sections per group). **B** Amelogenin and DSPP expression levels in the (a and a’; b and b’) mock group and (c and c’, d and d’) USP49-depletion group. Scale bar = 100 µm. a’, b’, c’, and d’ are higher magnification images of a, b, c, and d, respectively. Ab, ameloblast; ES, enamel space; D, dentin; Ob, odontoblast; P, pulp; dotted line, dentinoenamel junction; arrows, positive cells in ameloblasts; arrow heads, positive cells in odontoblasts. The microscopic images from six biological replicates with randomly selected sections per group.

To explore the function of USP49 in tooth development, mandibular molars at the bud stage electroporated with their respective constructs were cultured for two days in vitro. The mock-control and USP49-depleted samples were then transplanted into the subrenal capsules of mice to provide tooth germs with an in vivo biological environment for proper calcification. After 4 weeks, the fully calcified tissues and well-developed teeth surrounded by alveolar bone were surgically removed and examined for mineralization. In the mock group, micro-computed tomography images showed calcified tissues (*n* = 14/15) (Fig. 8A (a)), whereas in the USP49-depleted condition, fewer calcified tissues were observed with morphological defects (*n* = 11/15) (Fig. 8A (d)). Furthermore, the size of teeth obtained from the USP49-depleted group was much smaller than that of the mock group (Fig. 8A (a, d)). Next, histological analysis was performed to identify defects in enamel and dentin formation. H&E staining results showed that odontoblasts, ameloblasts, dental pulp, decalcified enamel (only enamel space remained), dentin, and periodontal ligament were well organized in the mock group (Fig. 8A (b, c)). However, the USP49-depleted samples showed malformed teeth with defective dentin, pulp, and abnormal enamel formation, with very few ameloblasts and odontoblasts (Fig. 8A (e, f)). The expression of amelogenin was prominent in the ameloblasts of the mock-group (Fig. 8B (a, a’)), while reduced amelogenin was observed in USP49-knockdown-group (Fig. 8B (c, c’)). Additionally, dentin sialophosphoprotein (DSPP) was expressed in odontoblasts and dentin in the mock-group (Fig. 8B (b, b’)) while, the expression was significantly reduced in the USP49-knockdown-group (Fig. 8B (d, d’)). Altogether, our findings suggest that USP49 is a key protein stabilizer of PAX9 and MSX1 that plays a critical role in amelogenesis and dentinogenesis during tooth development.

## Discussion

Crosstalk between the epithelium and mesenchyme, mediated by *PAX9* and *MSX1*, is required to ensure proper tooth development [6, 12]. In this study, we aimed to understand the DUB-mediated regulation of PAX9 and MSX1 protein abundance during odontogenesis. We used a CRISPR/Cas9-based DUB knockout library kit to screen and identify DUBs for PAX9 and MSX1 [31, 32]. Our screening system is more reliable and robust than methods that employ RNAi or shRNA molecules. Gene disruption using the CRISPR-Cas9 system is permanent, whereas RNAi- or shRNA-based methods produce only partial repression that is restored in a few days. Our screening results identified USP49 as a regulator of both PAX9 and MSX1 protein levels (Figs. 1 and 2).

The turnover of any protein is regulated by an interplay between ubiquitinases and deubiquitinases. The DUB-mediated removal of ubiquitin molecules from the target substrate plays a critical role in coordinating protein turnover [41–43]. Here, we demonstrated that USP49 interacts with and regulates the protein turnover of PAX9 and MSX1 by extending their half-lives (Fig. 3). Importantly, the loss of USP49 promoted the polyubiquitination of PAX9 and MSX1, thereby triggering rapid protein degradation (Fig. 4).

Reportedly, additional domains surrounding the catalytic active site of DUBs aid in the recognition of specific target substrates [39, 44–47]. Here, we demonstrated that USP49 with the UCH domain at the C-terminus is necessary for the interaction and deubiquitination of PAX9 and MSX1. In contrast, N-terminus USP49 lacking the UCH domain was unable to interact with PAX9 and MSX1 to deubiquitinate these proteins and influence their half-lives (Fig. 5). Human DPSCs possess self-renewal and multilineage differentiation abilities, and are able to regenerate a dentin pulp-like complex [48, 49]. We demonstrated that USP49 promoted odontogenic differentiation, as evidenced by increased ALP activity and calcium mineralization in hDPSCs. However, the USP49 with defective interaction failed to promote odontogenic differentiation in hDPSCs. The latter suggests that the interaction between USP49 and PAX9 or MSX1 is essential for normal odontogenesis (Fig. 5).

Neural crest (NC) cells are important for the development of dental tissues, including pulp, dentin, and periodontal tissues [50]. The loss of PAX9 in NC cells results in defects in the NC-derived mesenchymal components [51]. The upregulated expression of the neural plate border specifier MSX1 in the neural border is critical for the formation of bona fide NCs [52, 53]. Furthermore, it has been reported that high expression of Msx1 induces the production of NC cells in *Xenopus* embryo [52], whereas depletion of Msx1 inhibits this process. In this context, we demonstrated that USP49 knockout resulted in decreased PAX9 and MSX1 proteins with lower expression of NCLCs and dental mesenchymal cell markers suggesting that the expression of USP49 is essential for NC specification (Fig. 6).

Amelogenin is one of the main components of the developing tooth enamel matrix [54], and any malformation of amelogenin can lead to amelogenesis imperfecta [55]. Dentin sialophosphoprotein (DSPP), a marker of late odontoblast differentiation [56], is crucial for the mineralization of tooth dentin and has been implicated in dentinogenesis imperfecta [57]. Herein, we observed that the depletion of USP49 showed a significant decrease in PAX9 and MSX1 proteins in the dental mesenchyme (Fig. 7). Amelogenin was expressed in the ameloblasts of the mock group, whereas amelogenin expression persisted in the enamel matrix and was not evident in ameloblasts in the USP49-knockdown group (Fig. 8). DSPP was expressed in odontoblasts and dentin in the mock group, but its expression was significantly lower in the USP49-knockdown group (Fig. 8). In summary, the loss of USP49 results in abnormal tooth formation in the dentin, pulp, and enamel, including heavily disrupted ameloblasts and odontoblasts, suggesting USP49 as a novel regulator of odontogenesis.

## Supplementary information

Supplemental legends and tables

Supplementary Figures

Reproducibility checklist

Original Data File

## Data Availability

All data supporting the findings of this study are present in the the paper and/or the Supplementary information. Data supporting the present study are available from the corresponding author upon reasonable request.
