# Supplementary material for: Genome-wide screening for deubiquitinase subfamily identifies ubiquitin-specific protease 49 as a novel regulator of odontogenesis
Source: Cell Death Differ. 2022 Mar 10;29(9):1689–704. doi: 10.1038/s41418-022-00956-7 (PMC9433428; doi:10.1038/s41418-022-00956-7)
Supplement: Supplementary file 1 — Supplemental legends and tables [file 41418_2022_956_MOESM1_ESM.docx]

**Genome-wide screening for deubiquitinase subfamily identifies Ubiquitin-specific protease 49 as a novel regulator of odontogenesis**

Kamini Kaushal^1†^, Eun-Jung Kim^2†^, Apoorvi Tyagi^1†^, Janardhan Keshav Karapurkar^1^, Saba Haq^3^, Han-Sung Jung^2^*****, Kye-Seong Kim^1,4^***** and Suresh Ramakrishna^1,4^*****

^1^Graduate School of Biomedical Science and Engineering, Hanyang University, Seoul, 04763, South Korea

^2^Division in Anatomy and Developmental Biology, Department of Oral Biology, Taste Research Center, Oral Science Research Center, BK21 FOUR Project, Yonsei University College of Dentistry, Seoul, 03722, South Korea

^3^Department of Life Science, College of Natural Sciences, Hanyang University, Seoul, 04763, South Korea

^4^College of Medicine, Hanyang University, Seoul, 04763, South Korea

^†^ These authors contributed equally: Kamini Kaushal, Eun-Jung Kim, and Apoorvi Tyagi

***Corresponding authors**

SR (E-mail: [suri28@hanyang.ac.kr](mailto:suri28@hanyang.ac.kr), [suresh.ramakris@gmail.com](mailto:suresh.ramakris@gmail.com));

KS (E-mail: ks66kim@hanyang.ac.kr);

HSJ (E-mail: hsj8076@gmail.com)

**Running Title:** *Loss of ubiquitin-specific protease 49 impairs odontogenesis*

**Supplementary Information**

**Supplementary Fig. S1. Comparison of sgRNA efficiencies in targeting the USP49 gene in hDPSCs**

(A) Schematic representation of the RNA-guided engineered [nuclease](https://www.sciencedirect.com/topics/medicine-and-dentistry/nuclease) (RGEN) targeting the human *USP49* gene via the designed sgRNA1 (T1), sgRNA2 (T2), sgRNA3 (T3), and sgRNA4 (T4), which all target sequences in [exon](https://www.sciencedirect.com/topics/medicine-and-dentistry/exon) 1. The blue boxes represent exons. Red [arrowheads](https://www.sciencedirect.com/topics/biochemistry-genetics-and-molecular-biology/arrowhead) indicate the positions of sgRNAs that target the top strand. PAM sequences are presented in bold blue font.

(B) The cleavage efficiency of sgRNA1 (T1), sgRNA2 (T2), sgRNA3 (T3), and sgRNA4 (T4) was determined by the T7E1 assay in hDPSCs after [transfection](https://www.sciencedirect.com/topics/medicine-and-dentistry/genetic-transfection) with [plasmids](https://www.sciencedirect.com/topics/medicine-and-dentistry/plasmid) encoding [Cas9](https://www.sciencedirect.com/topics/medicine-and-dentistry/cas9) and sgRNA. Non-targeting sgRNA transfected cells were used as mock control (mock). The size marker (M) is shown. The red arrow indicates the expected position of the cleaved DNA bands. The numbers at the bottom of the gel indicate the mutation percentages measured by band intensity using ImageJ software. The data are representative of three independent experiments (n = 3).

**Supplementary Fig. S2. Expression of endogenous USP49, PAX9 and MSX1 proteins in different mammalian cell lines.** (a) USP49, (b) PAX9, (c) MSX1.

**Supplementary Fig. S3. USP49 regulates both PAX9 and MSX1 exogenous proteins**

(A) HEK293 cells were transiently transfected with sgRNA1, sgRNA3, and shRNA1, which all targeted *USP49*, along with ectopically expressed Myc-PAX9 to check exogenous protein levels. The presented immunoblots are representative of two independent experiments (n = 2).

(B) HEK293 cells were transiently transfected with sgRNA1, sgRNA3 and shRNA1, which all targeted *USP49*, along with ectopically expressed Flag-MSX1 to check exogenous protein levels. The presented immunoblots are representative of two independent experiments (n = 2).

(C) HEK293 cells were transfected with Myc-PAX9 and increasing concentrations of Flag-USP49. The presented immunoblots are representative of two independent experiments (n = 2).

(D) HEK293 cells were transfected with Flag-MSX1 and increasing concentrations of Flag-USP49. The presented immunoblots are representative of two independent experiments (n = 2).

(E) HEK293 cells were transfected with Myc-PAX9 and increasing concentrations of Flag-USP49CA. The presented immunoblots are representative of two independent experiments (n = 2).

(F) HEK293 cells were transfected with Flag-MSX1 and increasing concentrations of Flag-USP49CA. Protein expression was detected through the indicated antibodies and analyzed by Western blotting. GAPDH or β-actin were used as a loading control. The presented immunoblots are representative of two independent experiments (n = 2).

**Supplementary Fig. S4. USP49 reconstitution in USP49 depleted HEK293 cells**

(A) The rescue of Myc-PAX9 protein mediated by USP49 was analyzed by reconstitution with Flag-USP49. The presented immunoblots are representative of two independent experiments (n = 2).

(B) The rescue of Flag-MSX1 protein mediated by USP49 was analyzed by reconstitution with Flag-USP49. Protein expression was detected through the indicated antibodies and analyzed by Western blotting. GAPDH or β-actin were used as a loading control. The presented immunoblots are representative of two independent experiments (n = 2).

**Supplementary Fig. S5. Half-lives of the PAX9 and MSX1 proteins**

(A) The half-life of endogenous PAX9 protein, the cycloheximide assay was performed in hDPSCs for indicated time intervals as mentioned above the blot. The presented immunoblots are representative of three independent experiments (n = 3). Densitometric analysis of PAX9 expression (normalized to GAPDH) is reported under the blot and represents the mean of three independent experiments (n = 3).

(B) The half-life of endogenous MSX1 protein, the cycloheximide assay was performed in hDPSCs for different time intervals as indicated above the blot. The presented immunoblots are representative of three independent experiments (n = 3). Densitometric analysis of MSX1 expression (normalized to GAPDH) is reported under the blot and represents the mean of three independent experiments (n = 3).

(C) The half-life of endogenous PAX9 in hDPSCs transfected with either mock or Flag-USP49 and treated with CHX for the indicated time points. The presented immunoblots are representative of three independent experiments (n = 3). Densitometric analysis of PAX9 expression (normalized to GAPDH) is reported under the blot and represents the mean of three independent experiments (n = 3).

(D) The half-life of endogenous MSX1 in hDPSCs transfected with either Mock or Flag-USP49 and treated with CHX for the indicated time points. The protein band intensities were estimated using ImageJ software with reference to GAPDH control. The presented immunoblots are representative of three independent experiments (n = 3). Densitometric analysis of MSX1 expression (normalized to GAPDH) is reported under the blot and represents the mean of three independent experiments (n = 3).

**Supplementary Fig. S6. Effect of USP49 and catalytic mutant USP49CA on the half-lives of the PAX9 and MSX1 proteins**

(A) HEK293 cells were transfected with Myc-PAX9 and Flag-USP49. The presented immunoblots are representative of two independent experiments (n = 2).

(B) HEK293 cells were transfected with Flag-MSX1 and Flag-USP49. The presented immunoblots are representative of two independent experiments (n = 2).

(C) HEK293 cells were transfected with Myc-PAX9 and Flag-USP49CA. The presented immunoblots are representative of two independent experiments (n = 2).

(D) HEK293 cells were transfected with Flag-MSX1 and Flag-USP49CA. The presented immunoblots are representative of two independent experiments (n = 2).

HEK293 cells were treated with CHX (150 μg/mL), harvested at different times, and analyzed by Western blotting with the indicated antibodies. Band intensity was estimated using ImageJ software, normalized with GAPDH or β-actin, and graphically represented.

**Supplementary Fig. S7. mRNA expression of *PAX9* and *MSX1* in hDPSCs as determined by qRT-PCR**

(A) Effect of overexpressing Mock and USP49 on the mRNA level of *PAX9* in hDPSCs.

(B) Effect of overexpressing Mock and USP49 on the mRNA level of *MSX1* in hDPSCs.

The fold changes of mRNA levels were calculated for each individual experiment based on the mean of three technical replicates. The data presented here represent the mean ± SD of three independent experiments (n = 3). Non-significant (ns); statistical analysis was performed by Student’s t-test.

**Supplementary Fig. S8. PAX9 and MSX1 proteins undergo 26S proteasomal degradation**

(A) The PAX9 protein level was analyzed in hDPSCs treated with increasing concentrations of the proteasomal inhibitor, MG132 (0, 1.25, 2.5, 5, 7.5, 10 µM/mL). The presented immunoblots are representative of three independent experiments (n = 3). The data presented here represent the mean ± SD of three independent experiments (n = 3), (***P < 0.0001 by Student’s t-test).

(B) The MSX1 protein level was analyzed in hDPSCs treated with increasing concentrations of the proteasomal inhibitor MG132 (0, 1.25, 2.5, 5, 7.5, 10 µM/mL). The presented immunoblots are representative of three independent experiments (n = 3). The data presented here represent the mean ± SD of three independent experiments (n = 3), (***P < 0.0001 by Student’s t-test).

(C) Protein level of PAX9 in hDPSCs transfected with sgRNA1-USP49 and treated with or without MG132. The presented immunoblots are representative of three independent experiments (n = 3).

(D) Protein level of MSX1 in hDPSCs transfected with sgRNA1-USP49 and treated with or without MG132. The presented immunoblots are representative of three independent experiments (n = 3).

(E) HEK293 cells were transfected with Myc-PAX9 and HA-ubiquitin, immunoprecipitated with anti-Myc antibody, and immunoblotted with the indicated antibodies. The presented immunoblots are representative of two independent experiments (n = 2).

(F) HEK293 cells were transfected with Flag-MSX1 and HA-ubiquitin, immunoprecipitated with anti-Flag antibody, and immunoblotted with the indicated antibodies. The presented immunoblots are representative of two independent experiments (n = 2).

**Supplementary Fig. S9. USP49 deubiquitinates PAX9 and MSX1 proteins**

(A) HEK293 cells were transfected with Myc-PAX9, HA-ubiquitin, Flag-USP49, Myc-USP44, and shRNA targeting *USP49*. Deubiquitination of PAX9 was conﬁrmed by co-immunoprecipitation with the anti-Myc antibody and immunoblotting with the indicated antibodies. The presented immunoblots are representative of one independent experiment (n = 1).

(B) HEK293 cells were transfected with Flag-MSX1, HA-ubiquitin, Flag-USP49, Myc-USP44, and shRNA targeting USP49. Deubiquitination of MSX1 was conﬁrmed by co-immunoprecipitation with the anti-Flag antibody and immunoblotting with the indicated antibodies. The presented immunoblots are representative of one independent experiment (n = 1).

**Supplementary Fig. S10. The effect of N- and C-terminus USP49 on PAX9 and MSX1 proteins**

(A) HEK293 cells were transfected with increasing concentrations of Flag-USP49-N to check the protein levels of Myc-PAX9. The presented immunoblots are representative of three independent experiments (n = 3).

(B) HEK293 cells were transfected with increasing concentrations of Flag-USP49-C to check the protein levels of Myc-PAX9. The presented immunoblots are representative of three independent experiments (n = 3).

(C) HEK293 cells were transfected with increasing concentrations of Flag-USP49-N to check the protein levels of Myc-MSX1. The presented immunoblots are representative of three independent experiments (n = 3).

(D) HEK293 cells were transfected with increasing concentrations of Flag-USP49-C to check the protein levels of Myc-MSX1. The presented immunoblots are representative of three independent experiments (n = 3).

(E) The half-life of Myc-PAX9 in HEK293 cells transfected with either mock, Flag-USP49-N and Flag-USP49-C and treated with CHX for the indicated time points. The presented immunoblots are representative of three independent experiments (n = 3).

(F) The half-life of Myc-MSX1 in HEK293 cells transfected with either mock, Flag-USP49-N and Flag-USP49-C and treated with CHX for the indicated time points. The protein band intensities were estimated using ImageJ software with reference to GAPDH control. The band intensity for PAX9/GAPDH or MSX1/GAPDH were represented below the blots. The presented immunoblots are representative of three independent experiments (n = 3).

**Supplementary Fig. S11. Generation of single cell-derived knockout clones of USP49 in hESCs**

(A) T7E1 screening of single cell-derived USP49 knockout in hESCs transfected with a plasmid encoding Cas9 along with sgRNA2 and sgRNA3 targeting the *USP49* gene. The lane represented with red is T7E1 positive clones. The data is representative of one independent experiment (n = 1).

(B) Sequencing results from T7E1-positive single cell-derived USP49KO clone. DNA sequences of the wild-type (WT) and USP49 mutant clone, with CRISPR recognition sites shown in red and the protospacer adjacent motif (PAM) sequence in blue and bold characters. Dashes indicate deleted bases (the number of deleted bases are described in the parentheses; The number of occurrences is shown in parentheses; X3 and X4 indicate the number of each clone).

(C) mRNA expression of the *USP49* gene was estimated by qRT-PCR in mock and USP49KO-hESCs clone.

(D) mRNA expression of pluripotent markers (*OCT4*) was estimated by qRT-PCR in mock and USP49KO-hESCs clone. The relative mRNA expression levels are shown after normalization to *GAPDH* mRNA expression. The fold changes of mRNA levels were calculated for each individual experiment based on the mean of three technical replicates. The data presented here represent the mean ± SD of three independent experiments (n = 3) (***P < 0.0001 and non-significant (ns)), by Student’s t-test.

**Supplementary Fig. S12. Generation of single cell-derived knockout clones of USP49 in hiPSCs**

(A) T7E1 screening of single cell-derived USP49 knockout in hiPSCs transfected with a plasmid encoding Cas9 and sgRNA2 targeting the *USP49* gene. The lane represented with red is T7E1 positive clones. The data is representative of one independent experiment (n = 1).

(B) Sequencing results from T7E1-positive single cell-derived USP49KO clones. DNA sequences of the wild-type (WT) and USP49 mutant clone, with CRISPR recognition sites shown in red and the protospacer adjacent motif (PAM) sequence in blue and bold characters. Dashes indicate deleted bases (the number of deleted bases are described in the parentheses; The number of occurrences (X2 and X1; X9 and X7) is shown in parentheses for USP49KO-clone#25 and USP49KO-clone#28 respectively; out-of-frame and in-frame mutations are indicated).

(C) USP49 protein expression in mock and USP49KO-hiPSC clones was analyzed by Western blot. The presented immunoblots are representative of two independent experiments (n = 2).

(D) mRNA expression of the *USP49* gene was estimated by qRT-PCR in mock and USP49KO-hiPSC clones.

(E) mRNA expression of pluripotent markers (*OCT4*) was estimated by qRT-PCR in mock and USP49KO-hiPSC clones. The data presented here are the mean ± SD of three independent experiments (***P < 0.0001 and non-significant (ns), by ANOVA followed by Tukey's post hoc test. The fold changes of mRNA levels were calculated for each individual experiment based on the mean of three technical replicates. The data presented here represent the mean ± SD of three independent experiments (n = 3) (***P < 0.0001 and non-significant (ns)), by Student’s t-test.

(F) Expression of endogenous pluripotency markers (SOX2, OCT4, SSEA-4 and NANOG) were confirmed by immunofluorescence staining in mock and USP49KO-hiPSCs. Scale bar: 100 µm. The presented microscopic images are representative of two independent experiments (n = 2).

(G) Endogenous expression of USP49, MSX1 and PAX9 was confirmed by immunofluorescence staining in mock and USP49KO-hiPSCs. Scale bar: 100 µm. The presented microscopic images are representative of two independent experiments (n = 2).

**Supplementary Fig. S13. Characterization of hESC-derived NCLCs**

(A) Scheme representation of the differentiation process from human pluripotent stem cells into NCLCs.

(B) mRNA expression of neural crest-specific transcriptional factors (*NOTCH1*, *NOTCH2*, and *SLUG*) in mock and USP49KO was determined by qRT-PCR. The data presented here are the mean ± SD of three independent experiments. For *NOTCH1* mRNA expression (non-significant (ns) for mock-hESCs versus USP49KO-hESCs, *P < 0.05 for mock-NCLCs versus USP49KO-NCLCs). For *NOTCH2* mRNA expression (non-significant (ns) for mock-hESCs versus USP49KO-hESCs, *P < 0.05 for mock-NCLCs versus USP49KO-NCLCs). For *SLUG* mRNA expression (non-significant (ns) for mock-hESCs versus USP49KO-hESCs, *P < 0.05 for mock-NCLCs versus USP49KO-NCLCs). The fold changes of mRNA levels were calculated for each individual experiment based on the mean of three technical replicates. The data presented here represent the mean ± SD of three independent experiments (n = 3). Statistical significance was analyzed by ANOVA followed by Tukey's post hoc test.

**Supplementary Fig. S14. Characterization of hiPSCs-derived NCLCs**

(A) Embryoid bodies (EBs) derived from mock and USP49KO-hiPSCs were subjected to differentiation into NCLCs. Cell morphology during differentiation was analyzed by bright field microscopy at the indicated times. The dotted outline indicates neural rosette formation during differentiation. Scale bar: 200 µm. The presented microscopic images are representative of three independent experiments (n = 3).

(B) Immunofluorescence staining was performed on mock and USP49KO-derived NCLCs on day 18 to analyze NCLCs markers (p75 and NESTIN). Scale bar: 200 µm. The presented microscopic images are representative of three independent experiments (n = 3).

(C) The effect of *USP49* gene disruption on the endogenous expression of dental mesenchymal markers (MSX1, PAX9 and LHX6) was analyzed by immunofluorescence staining on day 18 of NCLC differentiation. Scale bar: 100 µm. The presented microscopic images are representative of three independent experiments (n = 3).

(D) The effect of USP49 Knockout on the endogenous expression of dental mesenchymal markers (PAX9 and MSX1) and pluripotency marker (OCT4) in undifferentiated and differentiated cells were analyzed by immunoblotting with specific antibodies. GAPDH was used as an internal loading control. The presented immunoblots are representative of two independent experiments (n = 2).

**Supplementary Fig. S15. The transfection efficiency in mouse mandibles was checked based on green fluorescence emitted by the GFP-Cas9 construct after electroporation** Scale bar: 100 µm.

**Supplementary Fig. S16. A. H&E staining of tooth germs** with a. mock and b. USP49 depletion after 2 days of *in vitro* culture. Scale bar: 100 µm. Six biological replicates per group (n = 6) (H&E staining was performed from three biological replicates with randomly selected sections per group).

**Supplementary Table S1. Target sequences used for sgRNA plasmid construction**

| ***USP49*** | sgRNA1 | *Homo sapiens* | GCTAAGAAGCTCCCTCCTGG | Sense |
| --- | --- | --- | --- | --- |
|  | sgRNA2 | *Homo sapiens* | GCTCAATGATAACCCAGAGG | Sense |
|  | sgRNA3 | *Homo sapiens* | GCGGCCGCTATATTGAGGAC | Anti-sense |
|  | sgRNA4 | *Homo sapiens* | CTATATTGAGGACCACGCCC | Anti-sense |
| ***USP49*** | sgRNA1 | *Mus musculus* | GCTGAGAAGCTCCCTCCTGG | Sense |

| ***USP49*** | *Homo sapiens* | shRNA1 | GGTCATGCATCACGGGAAA |
| --- | --- | --- | --- |
|  |  | shRNA2 | GGACTACGTGCTCAATGAT |

**Supplementary Table S2. Target sequences used for shRNA plasmid construction**

**Supplementary Table S3. Oligonucleotide sequences used to get PCR amplicon for T7E1 assay**

| ***USP49*** | *Homo sapiens* | sgRNA1 and sgRNA2 | FP | TCGTGCCAGGTTGAGAAAGT |
| --- | --- | --- | --- | --- |
|  |  |  | FP1 | CTGCTTAGAGTGTGCCACCA |
|  |  |  | RP | AGCTCTTCTCGAACCACAGC |
|  |  |  | RP1 | AGCGTCCTGGCCAGCAGGCGCT |

**Supplementary Table S4. PCR amplicon and cleavage sizes after T7E1 assay**

| ***USP49***  *Homo sapiens* | sgRNA1 | 390 | 224+166 |
| --- | --- | --- | --- |
|  | sgRNA2 | 390 | 191+199 |
|  | sgRNA3 | 390 | 65+325 |
|  | sgRNA4 | 390 | 72+318 |

**Supplementary Table S5. Primers used for qRT-PCR**

| ***USP49*** | FP: 5’-AGTTTGGGAGTTCCCTCCTT-3’ |
| --- | --- |
|  | RP: 5’-GCTGCTCTCCTGTGTGGATA-3’ |
| ***MSX1*** | FP: 5’-AGCCCCCGAATACATCCTAT-3’ |
|  | RP: 5’-ATCCCATTTCCTTTGGCTTC-3’ |
| ***PAX9*** | FP: 5’-AACCAGCTGGGAGGAGTGTT-3’ |
|  | RP: 5’-TGATGTCACACGGTCGGATG-3’ |
| ***OCT4*** | FP: 5’-TAGCATTGAGAACCGTGTGAG-3’ |
|  | RP: 5’-ACTTGATCTTTTGCCCTTCTGG-3’ |
| ***SLUG*** | FP: 5’-GCCAAACTACAGCGAACTGG-3’ |
|  | RP: 5’-GATGGGGCTGTATGCTCCTG-3’ |
| ***NOTCH1*** | FP: 5’-CTTGTGTCAACGGCGGC-3’ |
|  | RP: 5’-TTGGGACCGCTGAAGCC-3’ |
| ***NOTCH2*** | FP: 5’-AGGTGTCAGAATGGAGGGGT-3’ |
|  | RP: 5’-GTGCAGAACTGTCCTGTCCA-3’ |
| ***GAPDH*** | FP: 5’-CATGTTCGTCATGGGTGTGAACCA-3’ |
|  | RP: 5’-AGTGATGGCATGGACTGTGGTCAT-3’ |
