# Supplementary material for: Genome-wide screening for deubiquitinase subfamily identifies ubiquitin-specific protease 49 as a novel regulator of odontogenesis
Source: Cell Death Differ. 2022 Mar 10;29(9):1689–704. doi: 10.1038/s41418-022-00956-7 (PMC9433428; doi:10.1038/s41418-022-00956-7)

Supplementary Fig. S1

A

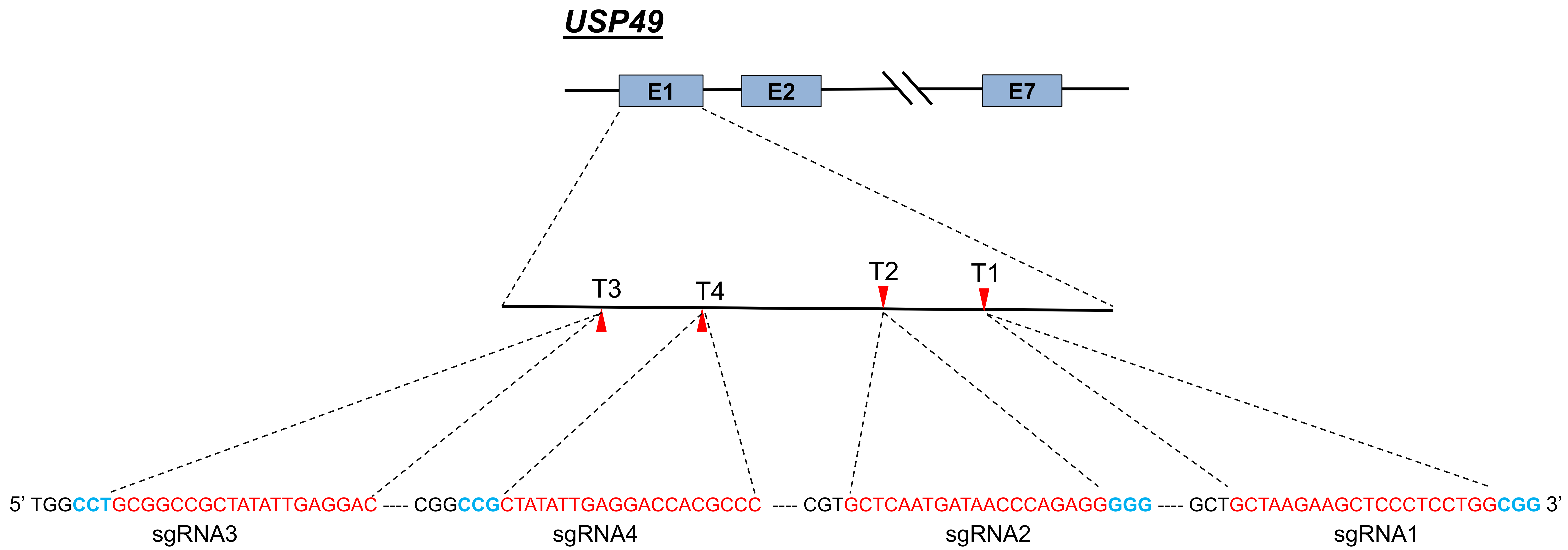

B

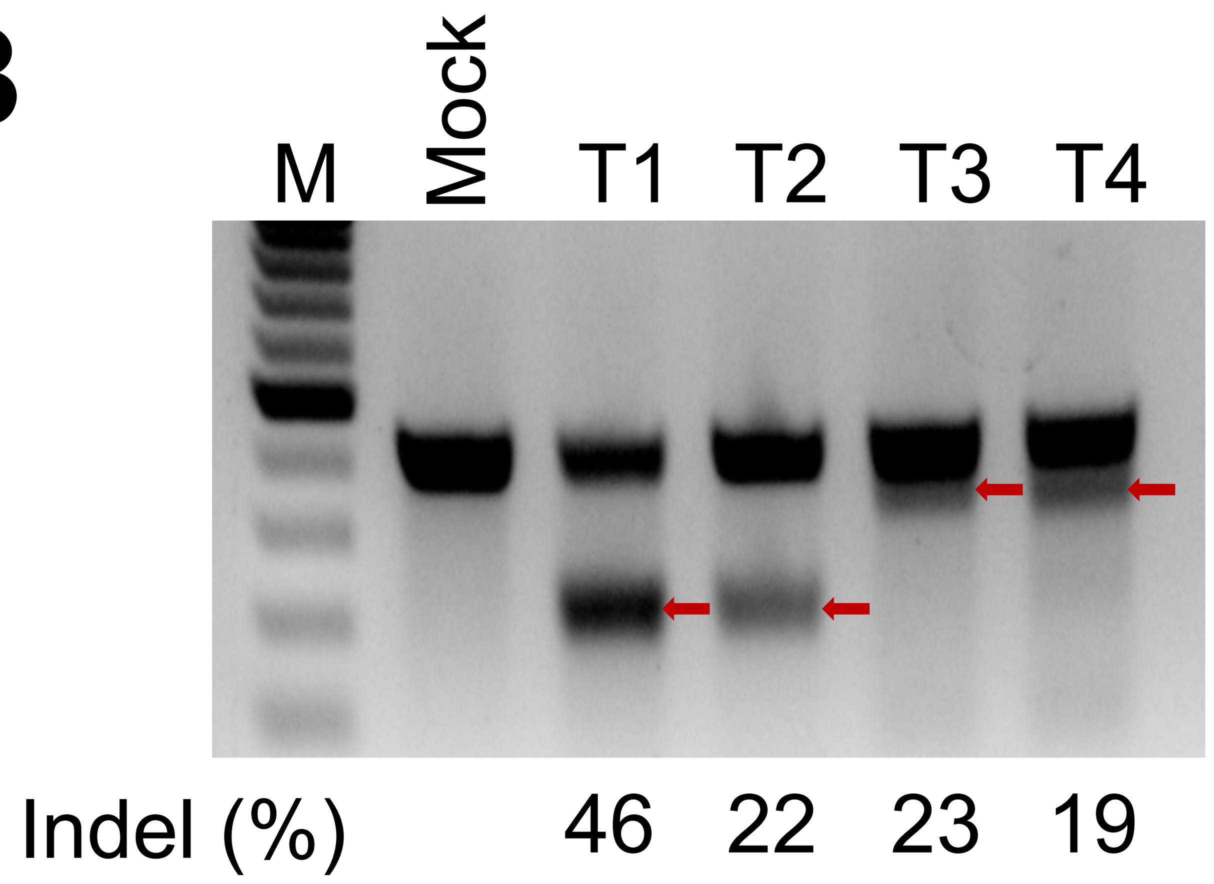

Supplementary Fig. S2

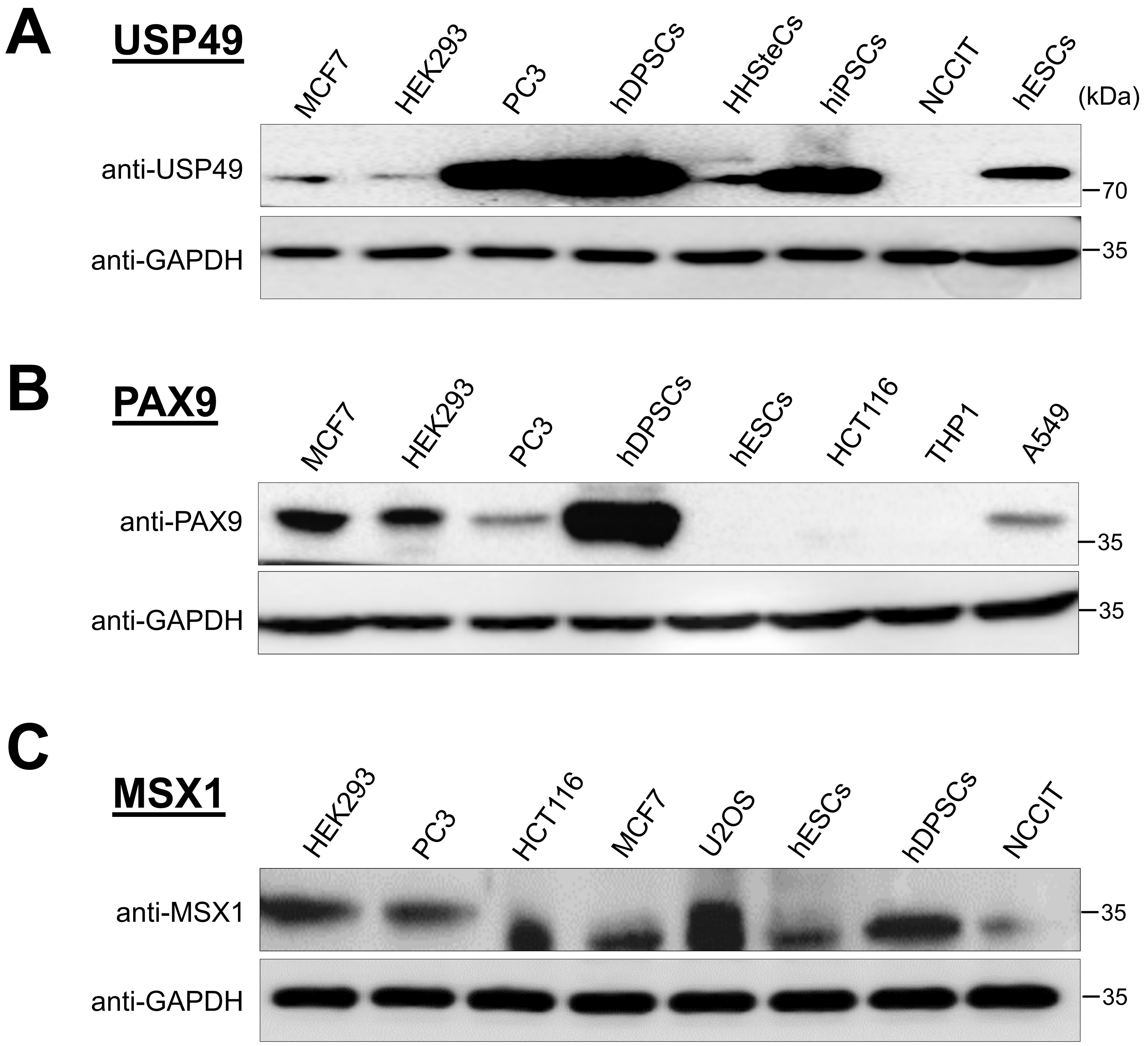

Supplementary Fig.S3

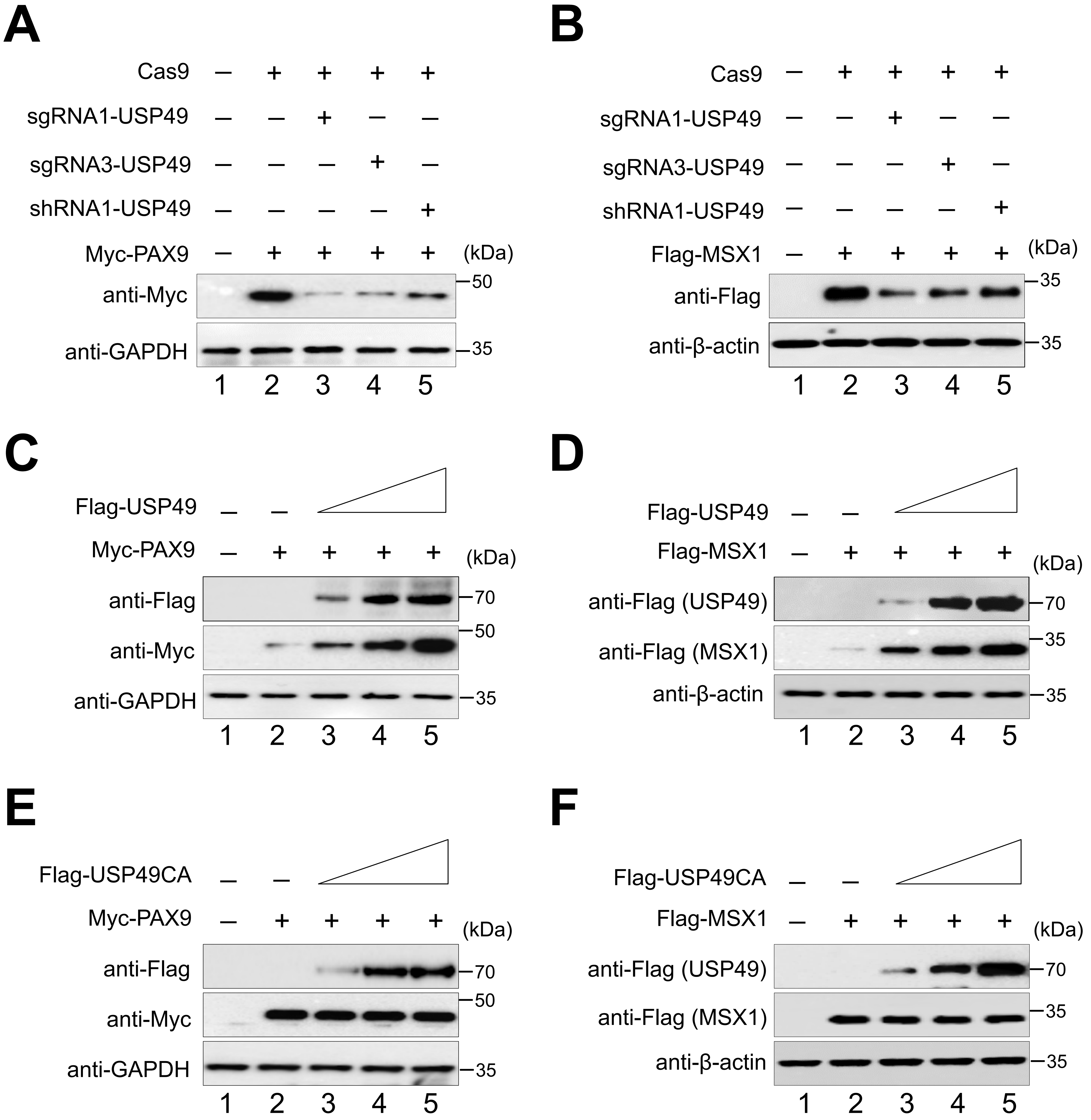

Supplementary Fig. S4

A

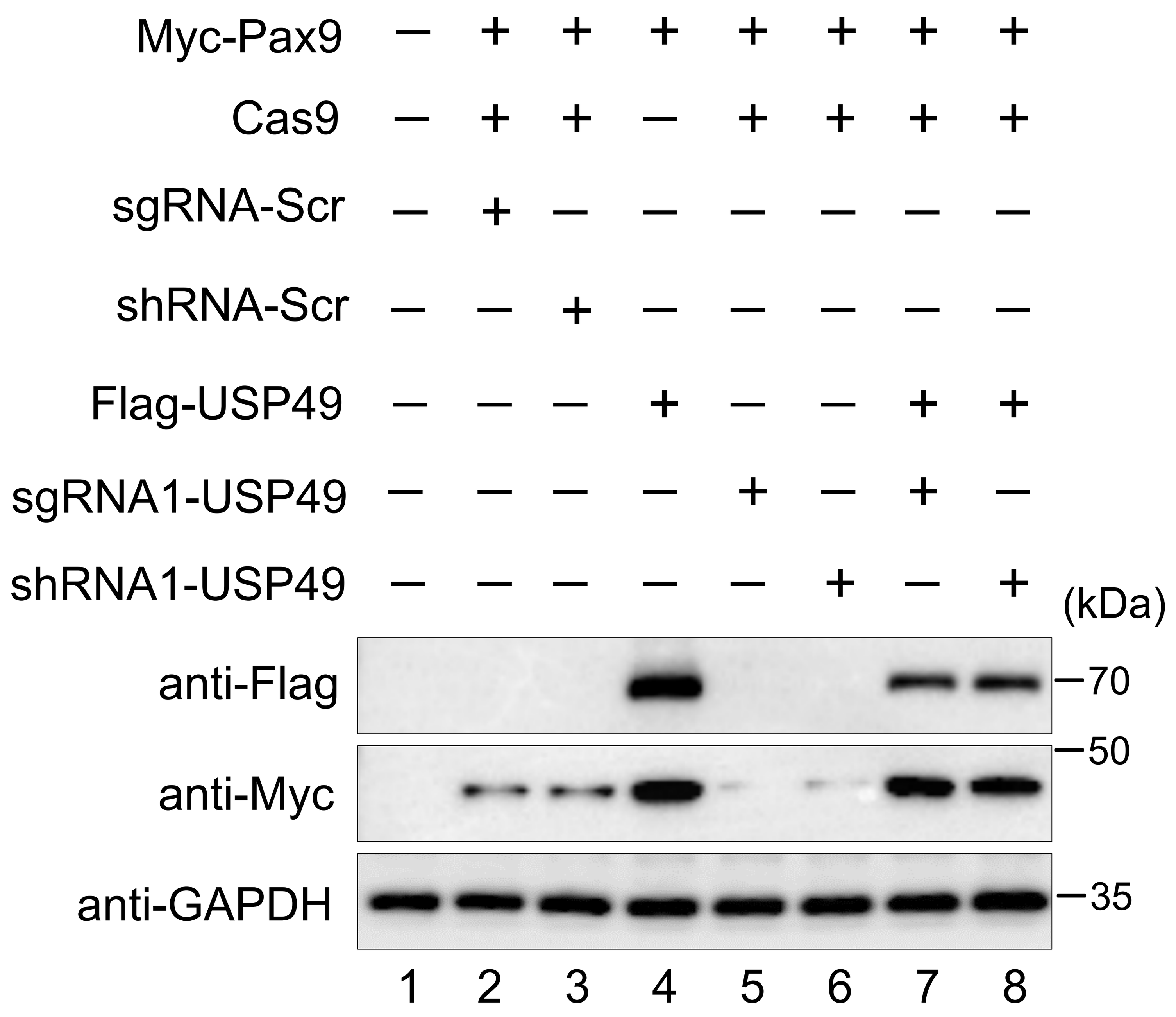

B

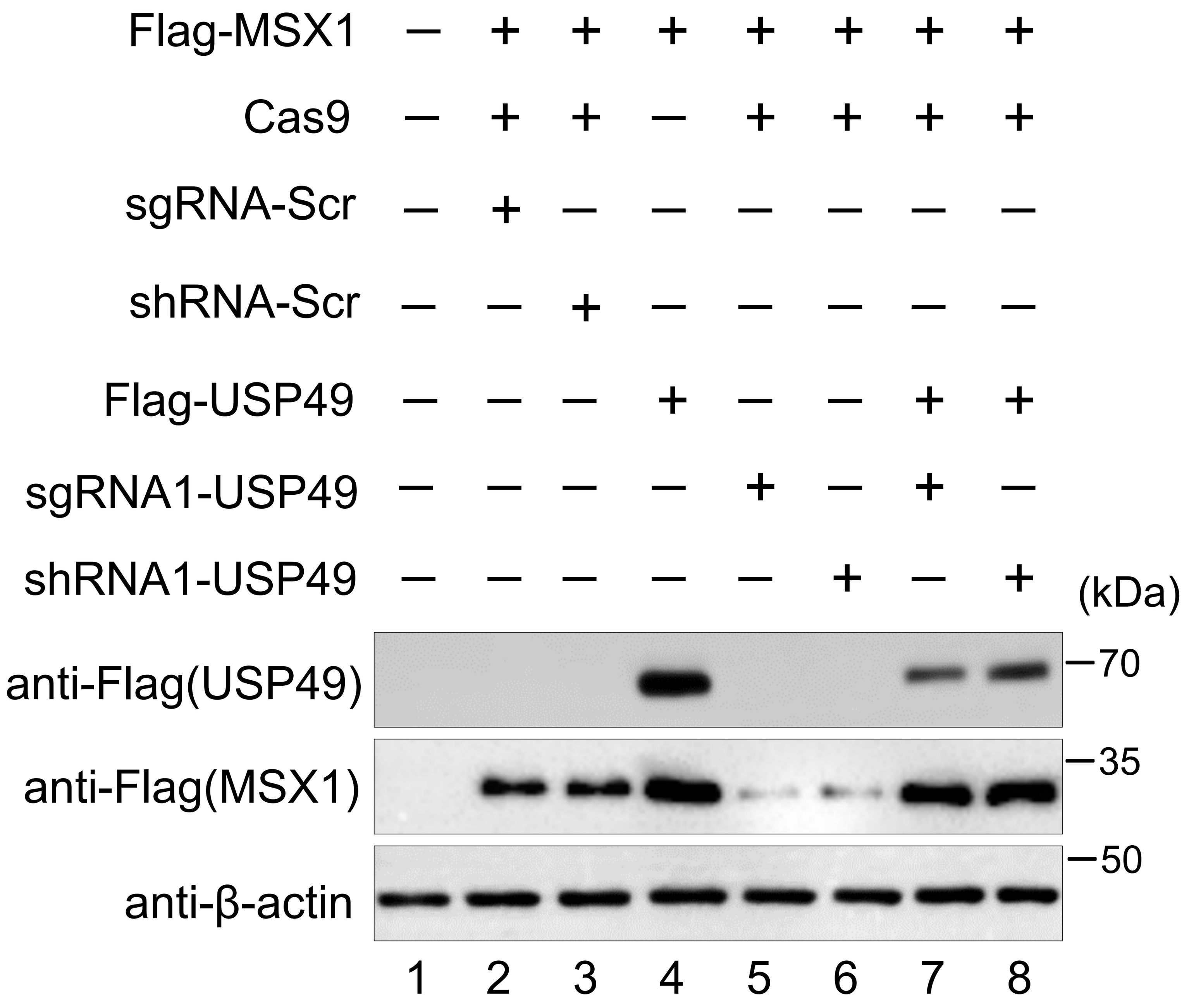

Supplementary Fig. S5

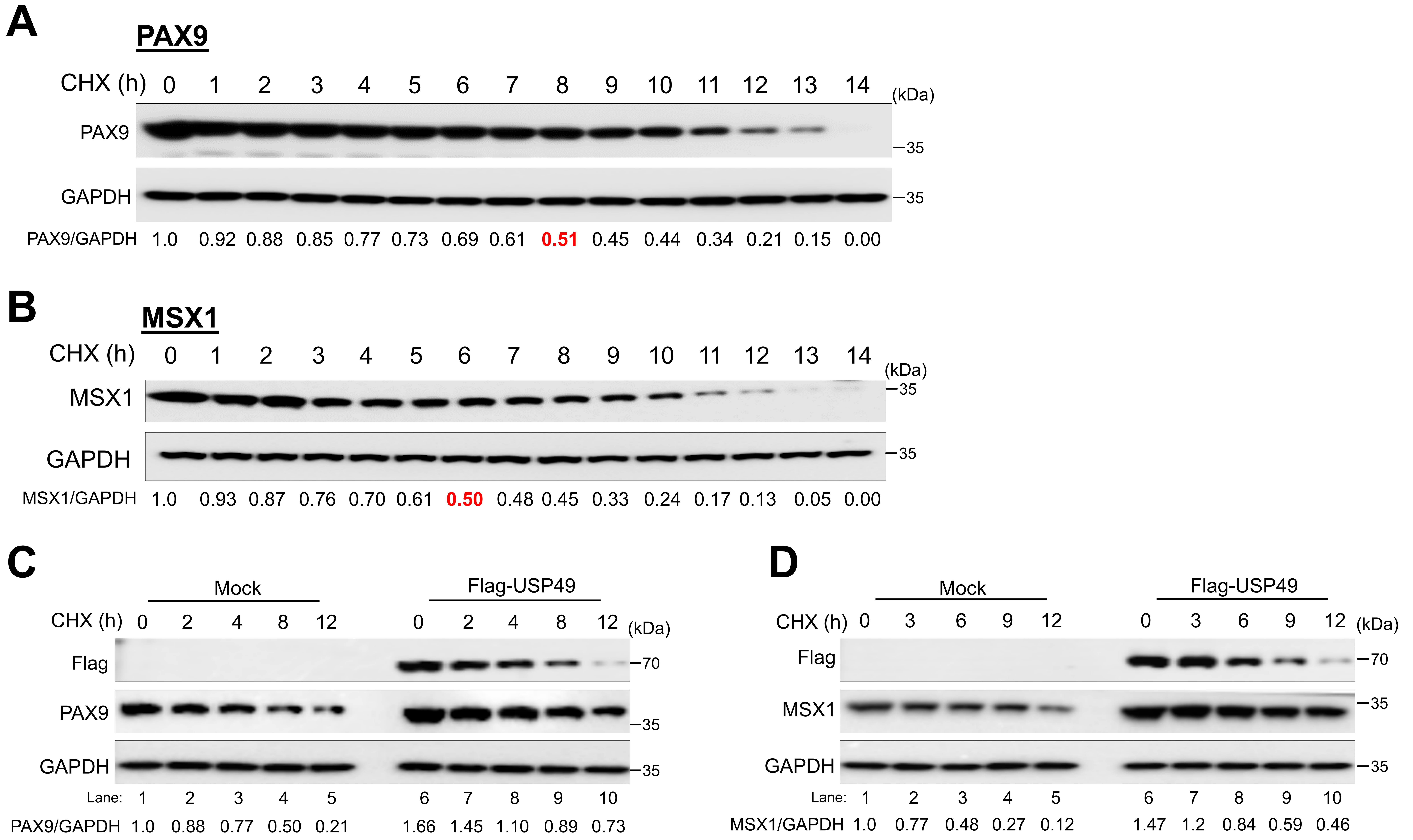

Supplementary Fig. S6

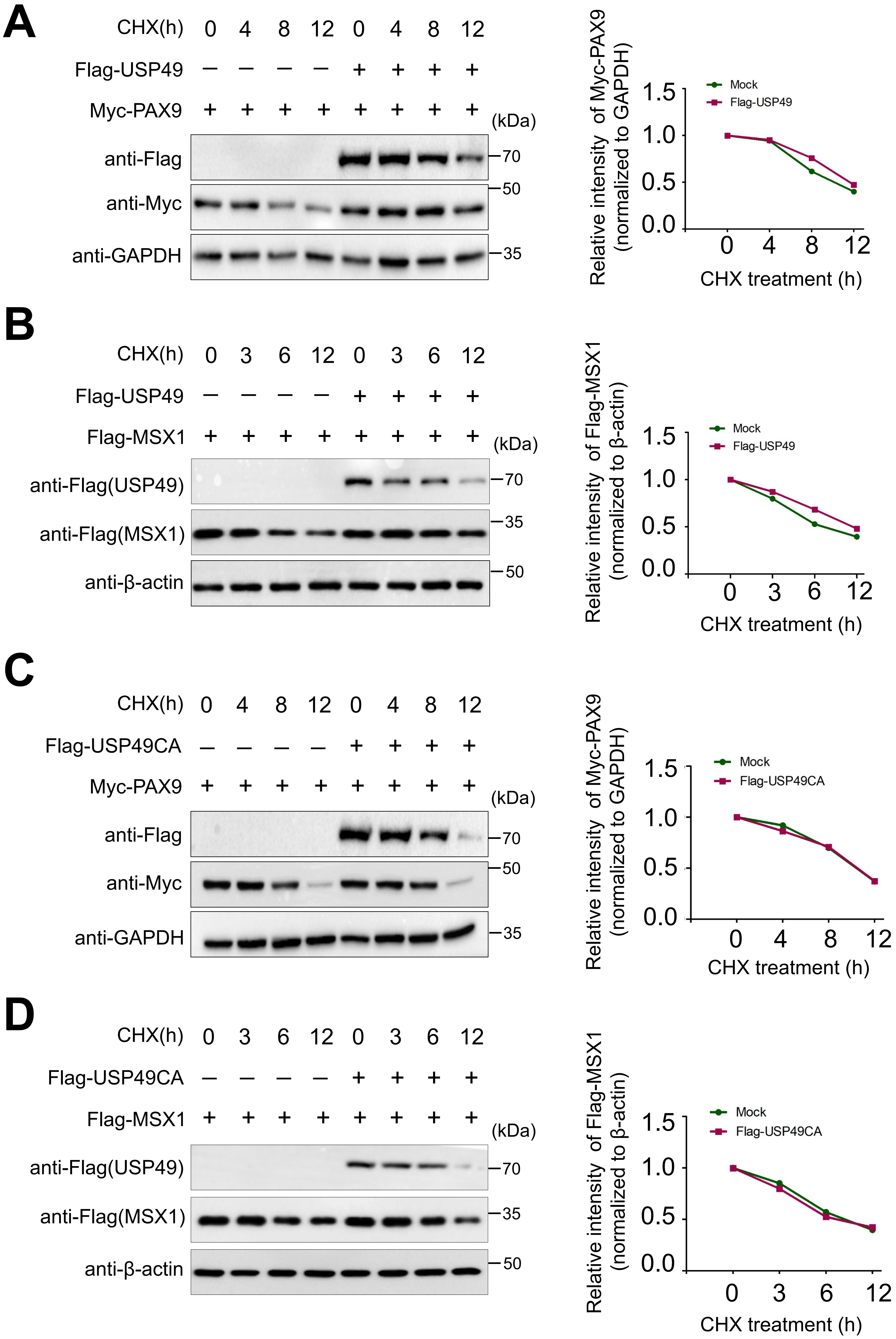

Supplementary Fig. S7

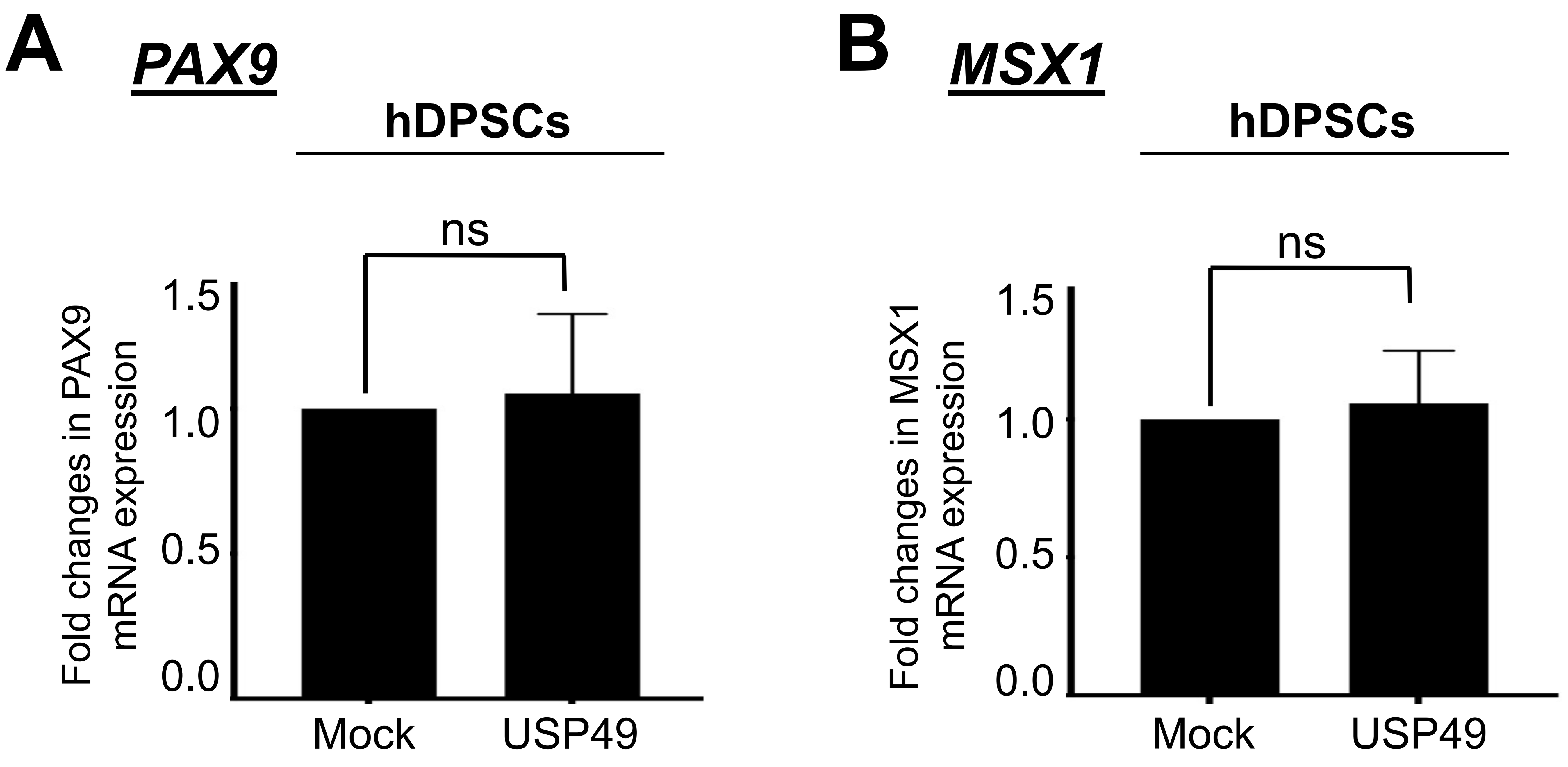

Supplementary Fig. S8

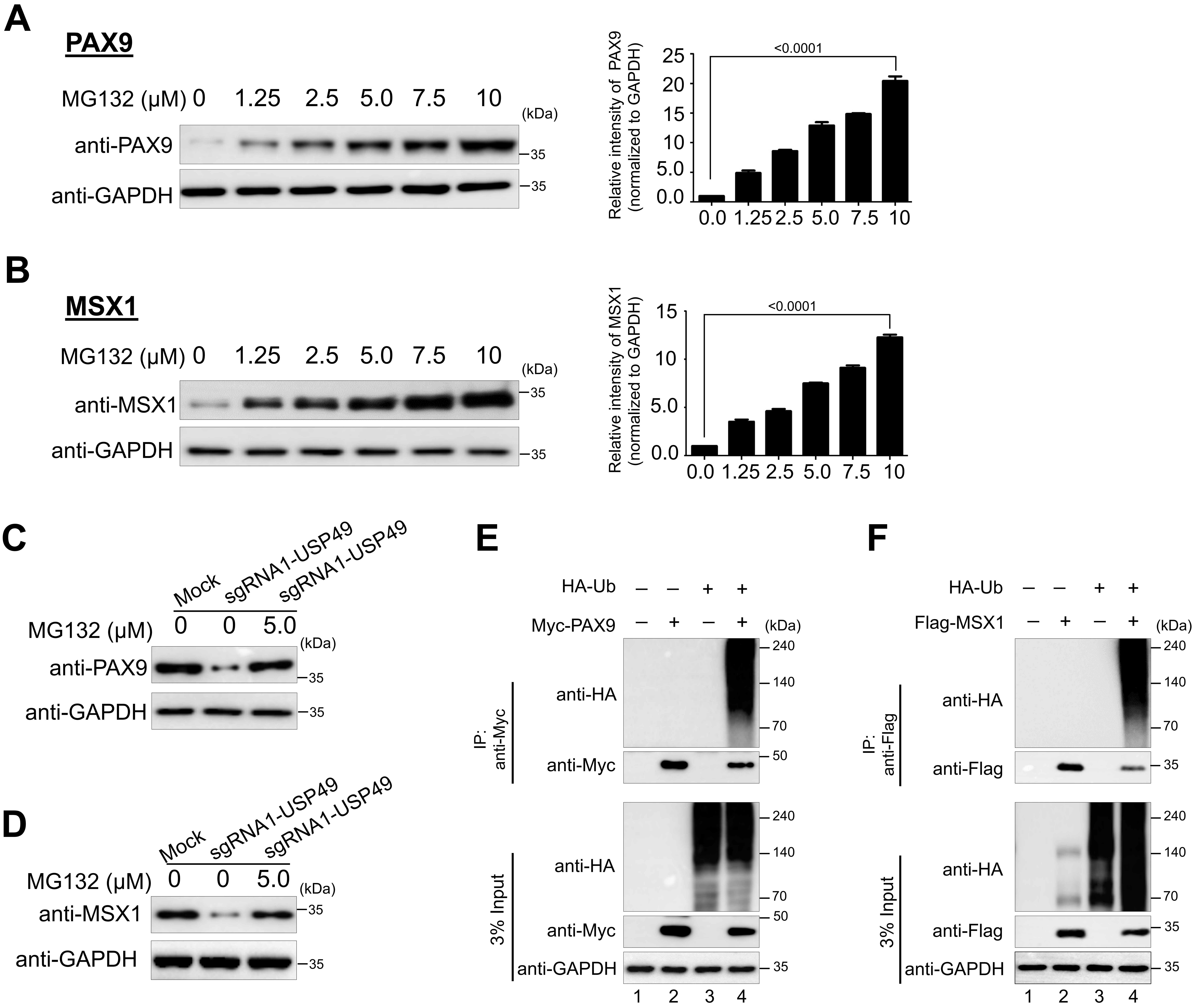

Supplementary Fig. S9

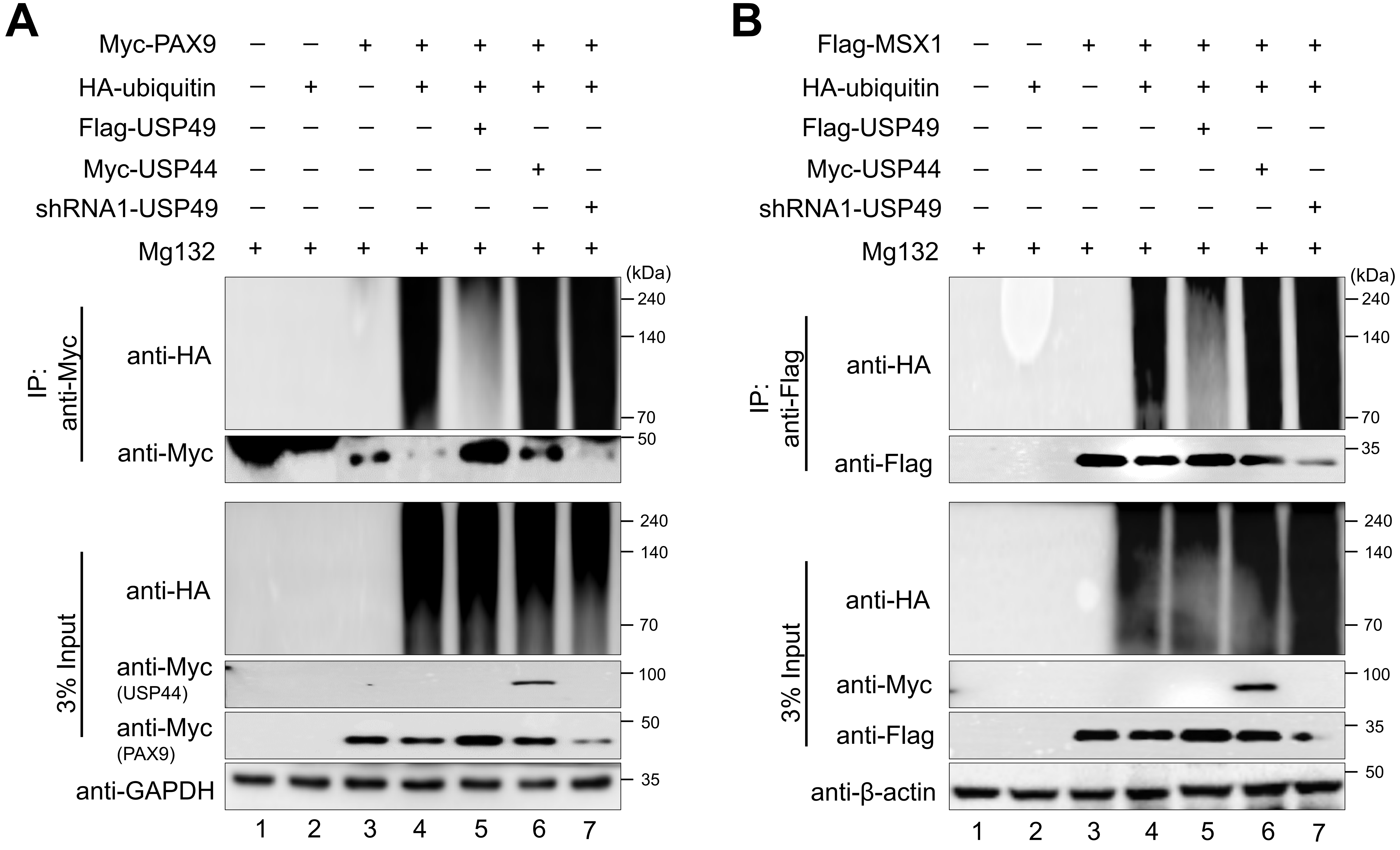

Supplementary Fig. S10

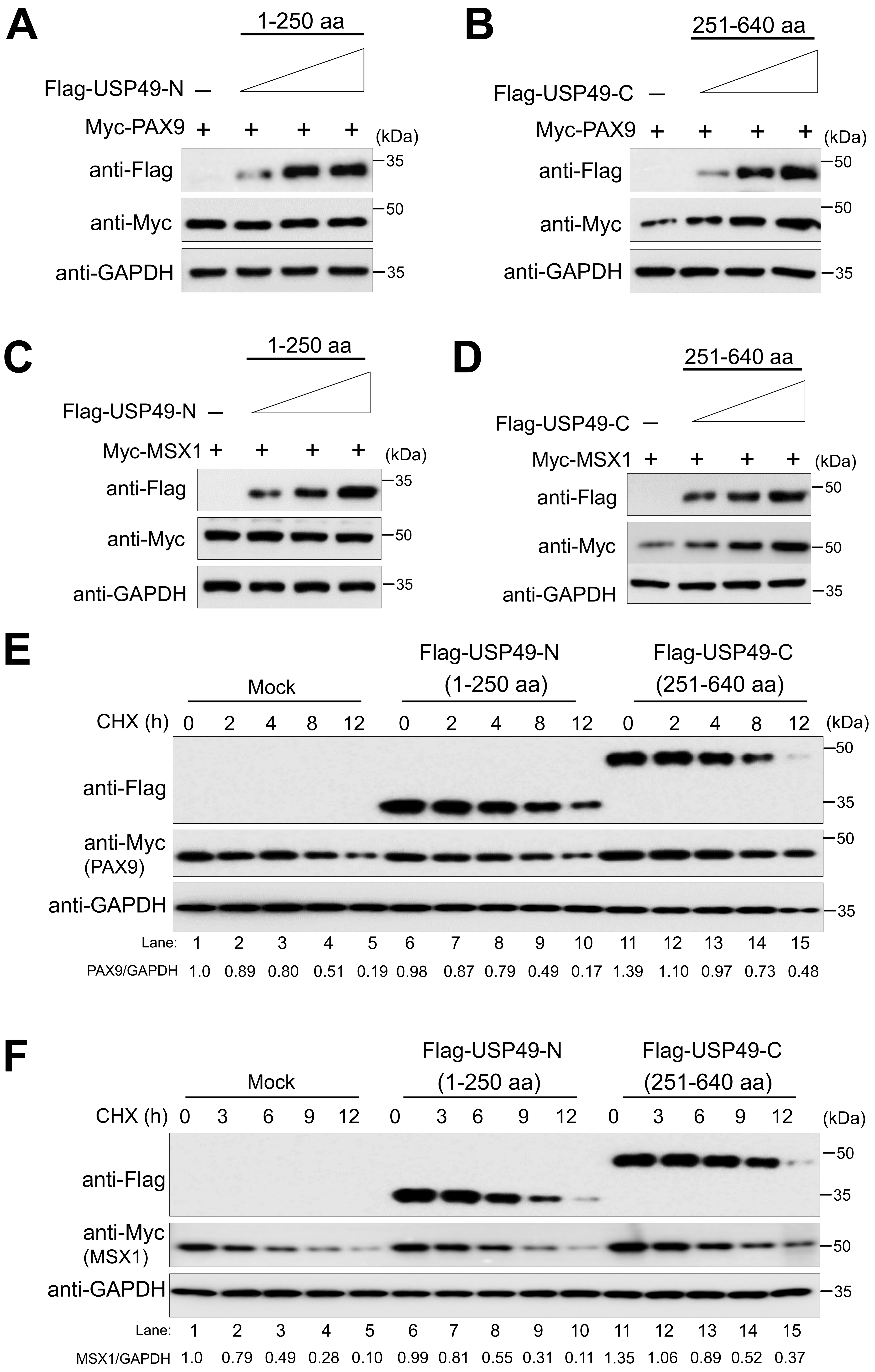

Supplementary Fig. S11

A

USP49

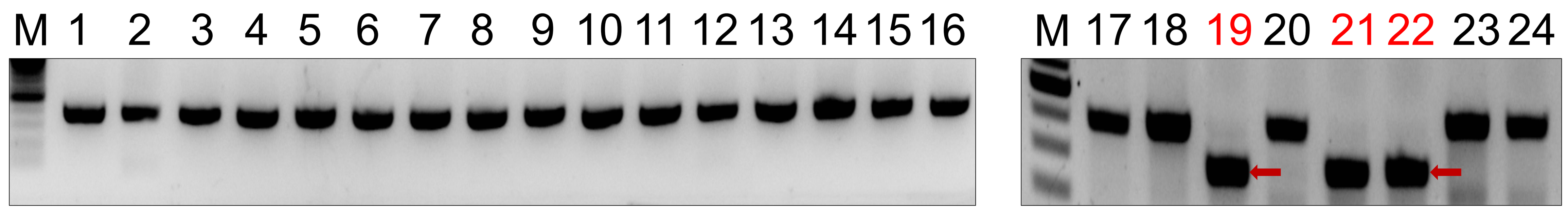

B

***USP49KO*-hESCs Clone#22**

```
                 sgRNA3                                  sgRNA2
TGGCCTGCGGCCGCTATATTGAGGAC-------------GCTCAATGATAACCCAGAGGGGGACCT (WT)
TGGCCTGCGG-----------------------------------------------GAGGGGGACCT (-125)(X3)
TGGCCTGCG------------------------------------------------GAGGGGGACCT (-126)(X4)
```

C

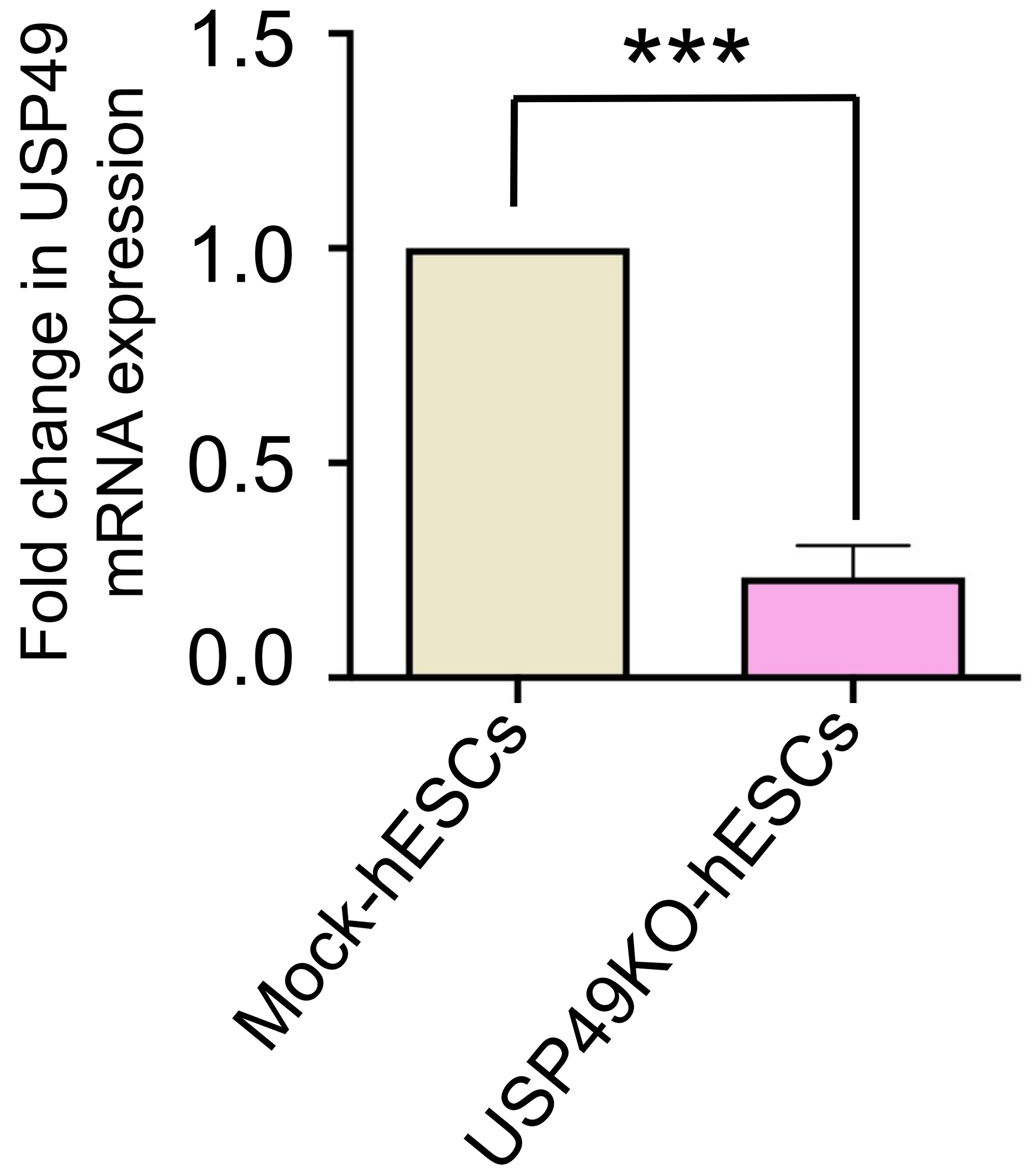

D

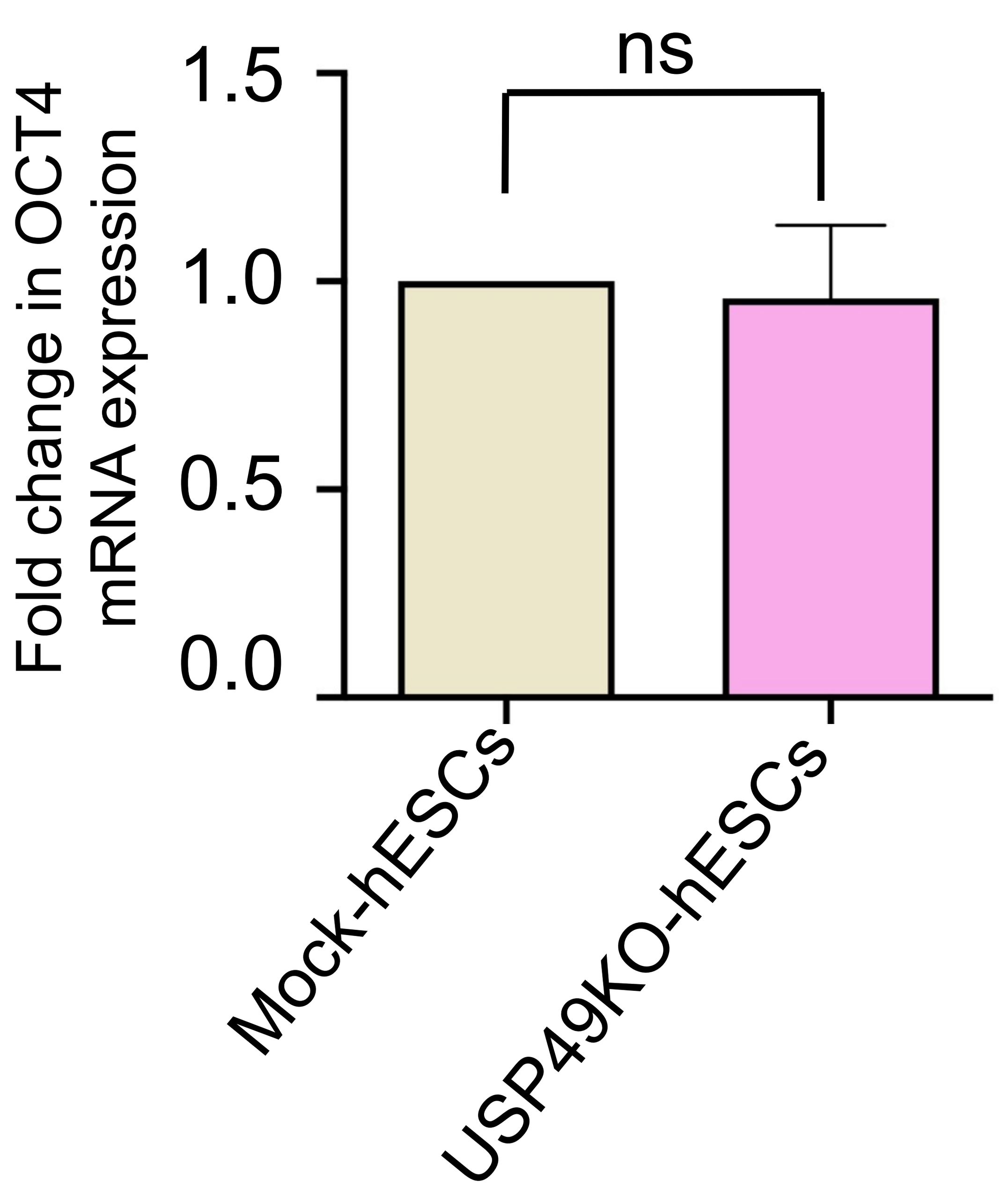

Supplementary Fig. S12

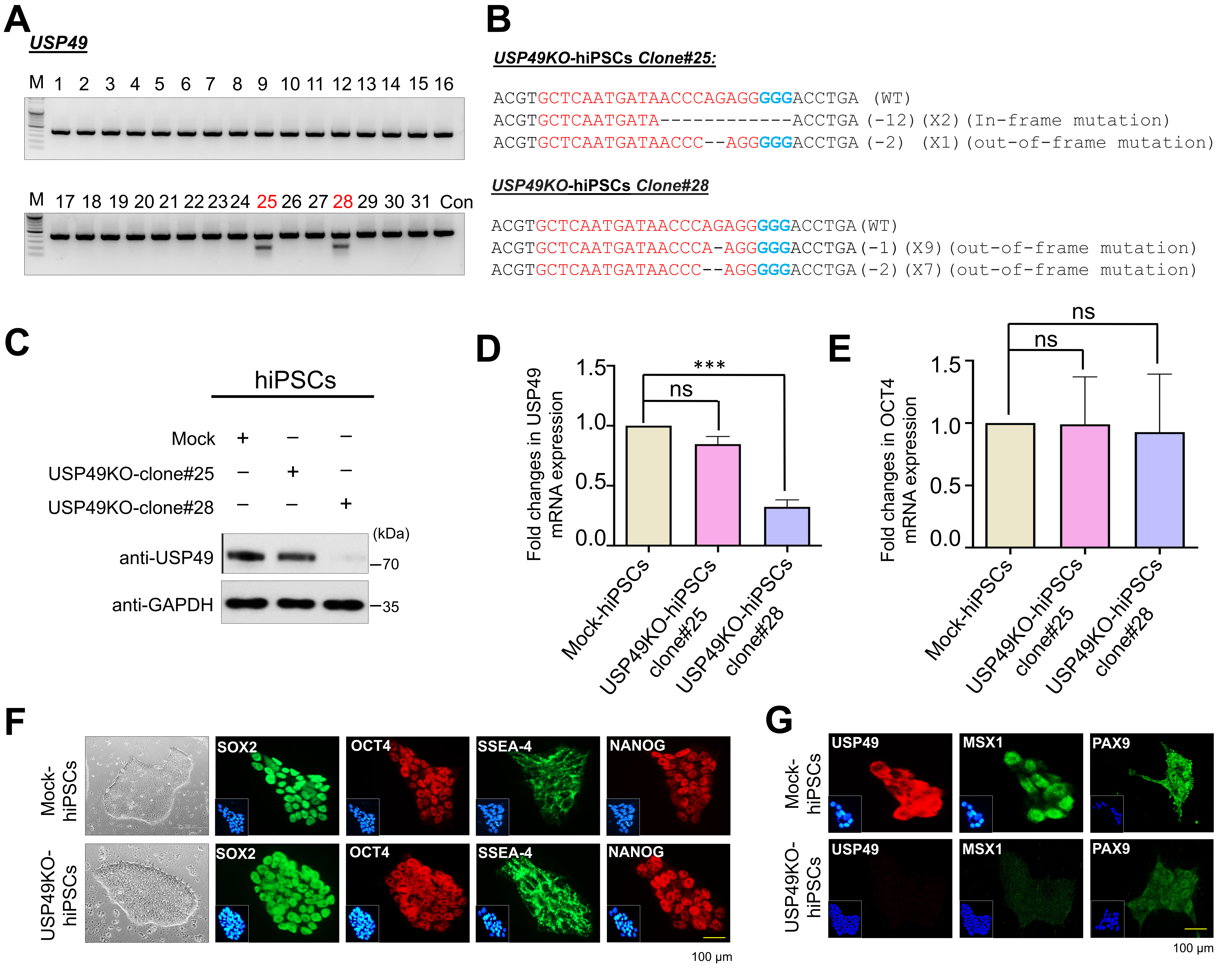

Supplementary Fig. S13

A

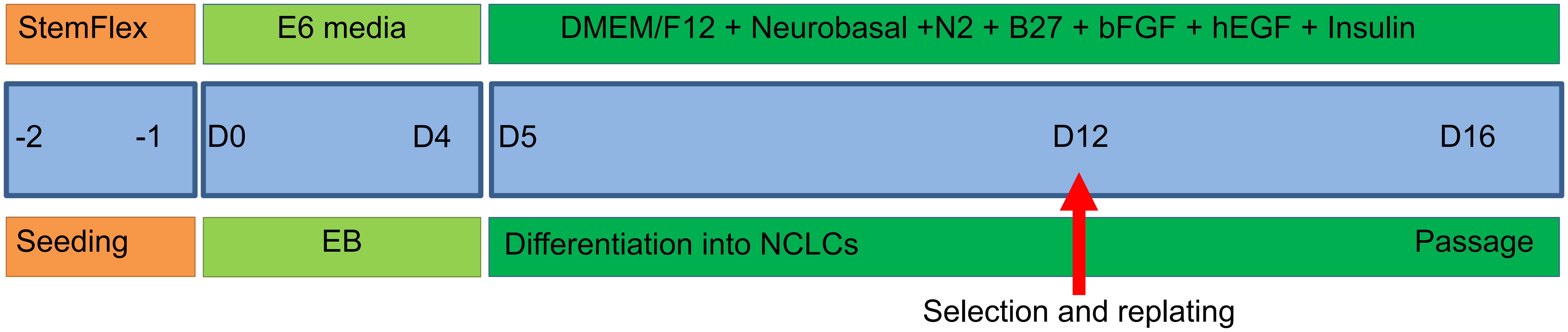

B

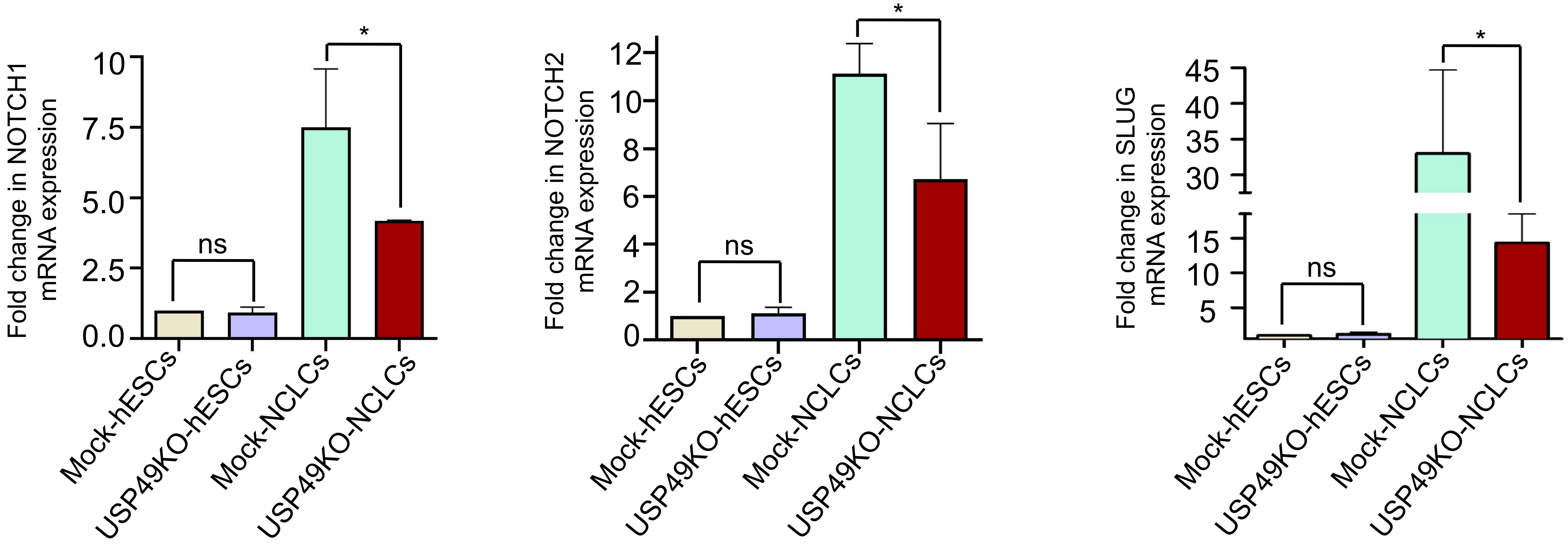

Supplementary Fig. S14

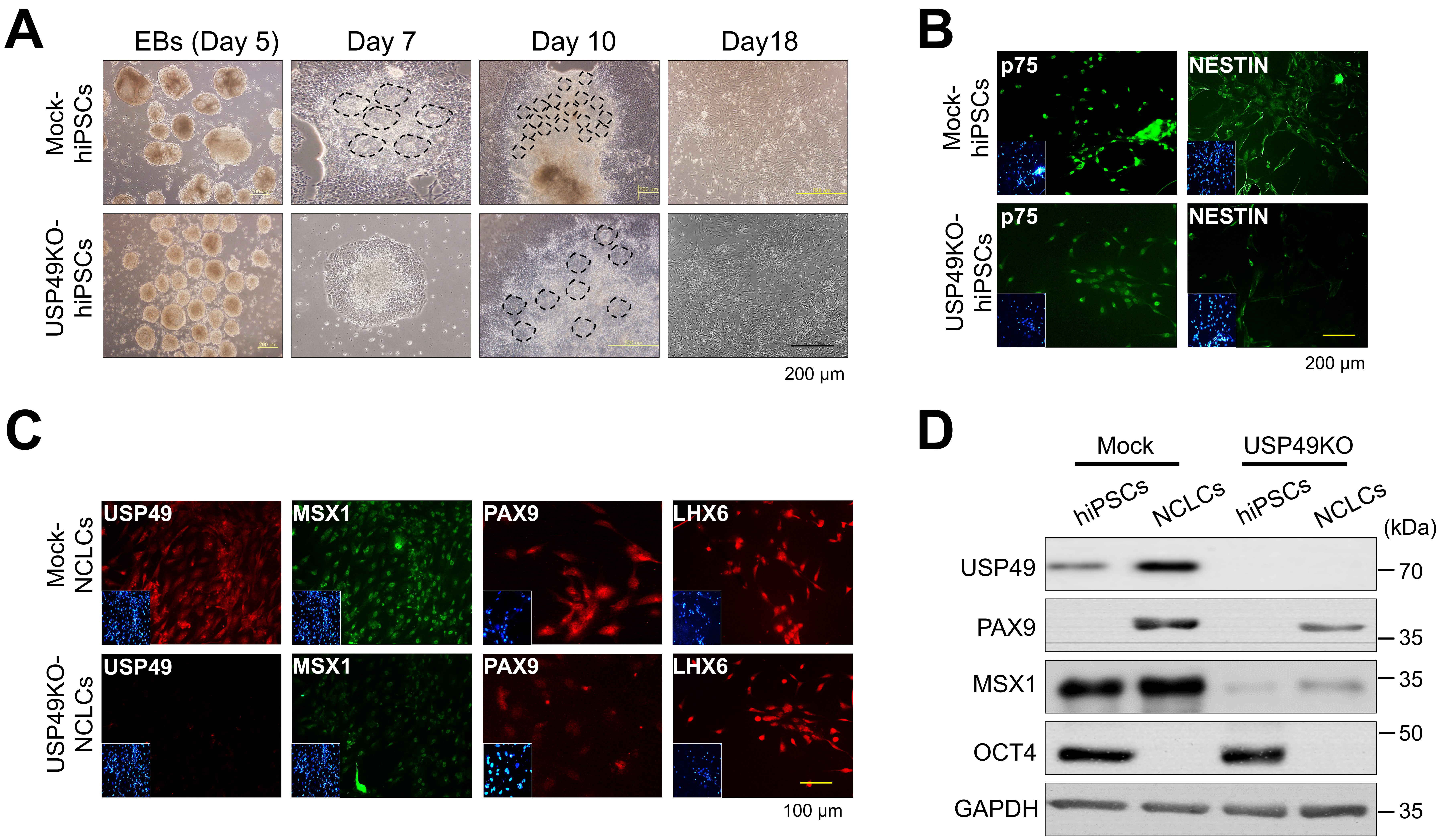

Supplementary Fig. S15

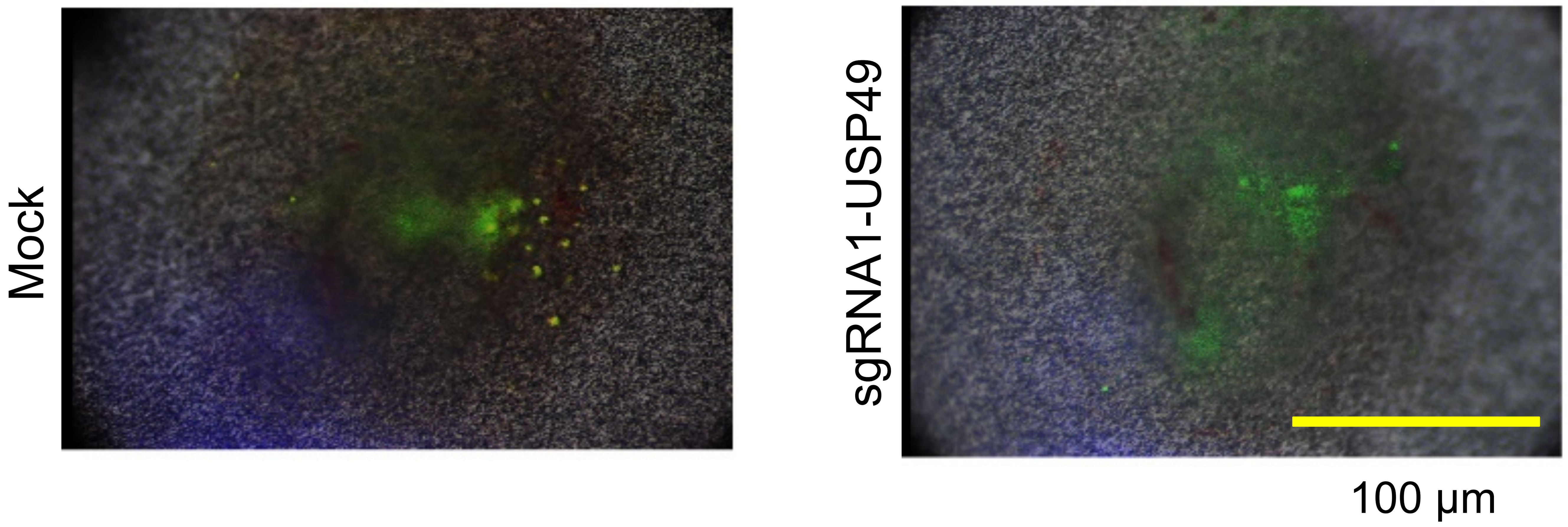

Supplementary Fig. S16

A

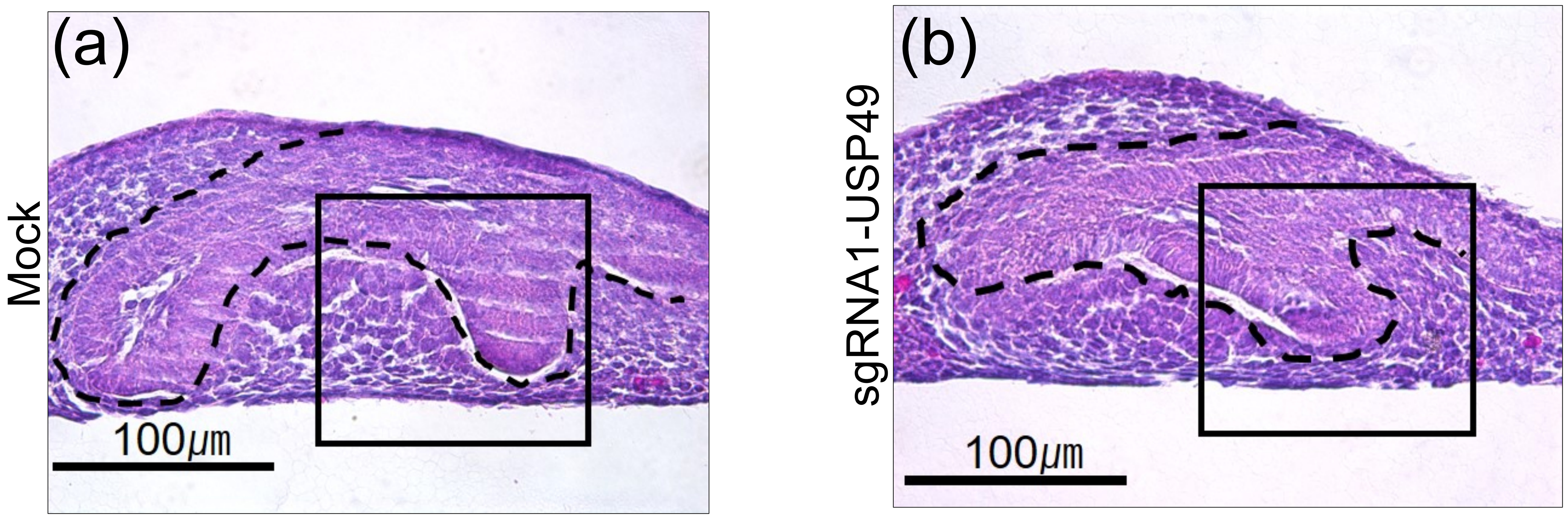

Supplement: Supplementary file 2 — Supplementary Figures [file 41418_2022_956_MOESM2_ESM.pdf]
