# Supplementary material for: Genome-wide screening for deubiquitinase subfamily identifies ubiquitin-specific protease 49 as a novel regulator of odontogenesis
Source: Cell Death Differ. 2022 Mar 10;29(9):1689–704. doi: 10.1038/s41418-022-00956-7 (PMC9433428; doi:10.1038/s41418-022-00956-7)

Uncropped blots of Fig. 1

A

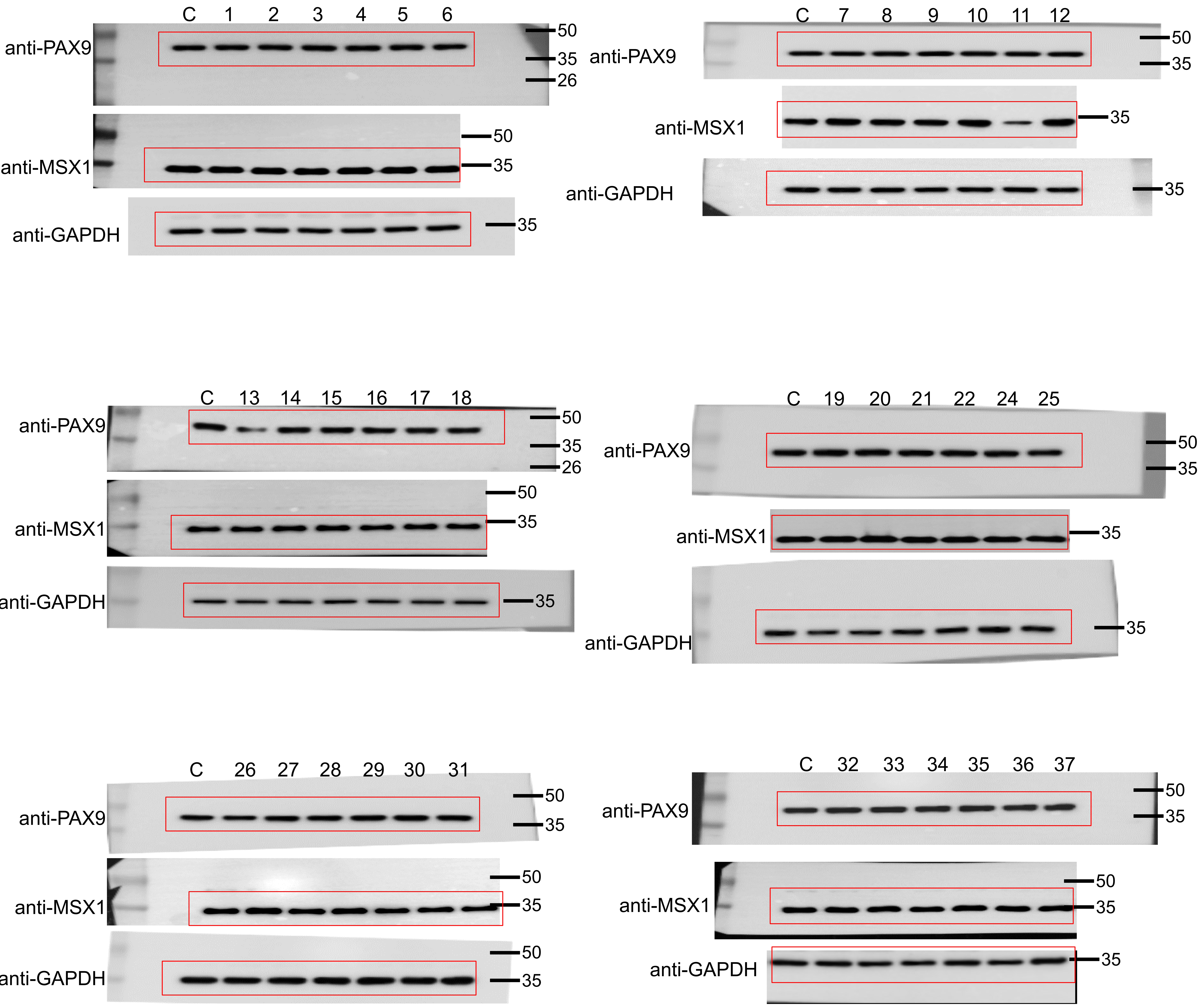

Uncropped blots of Fig. 1

A

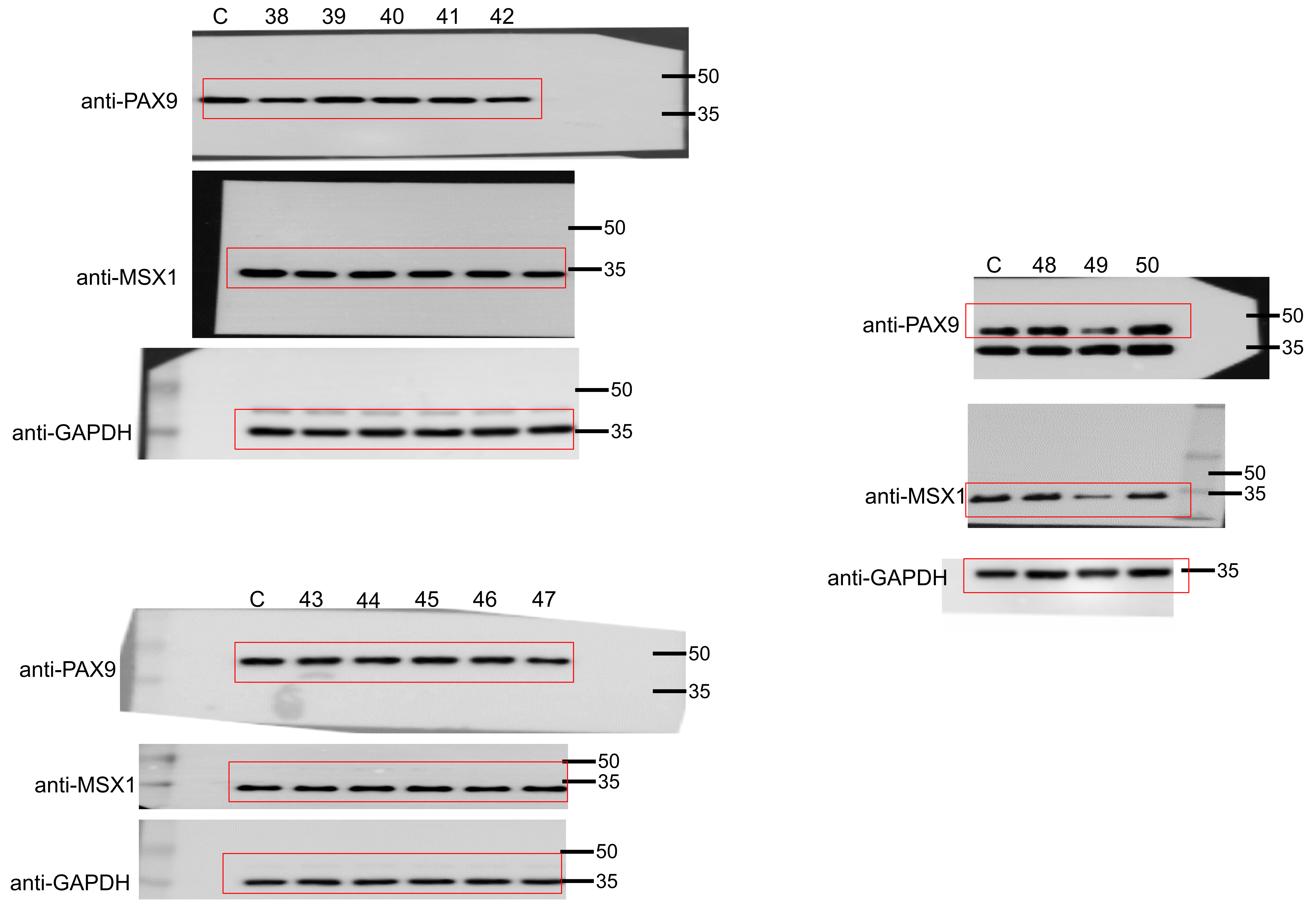

Uncropped blots of Fig. 1

B

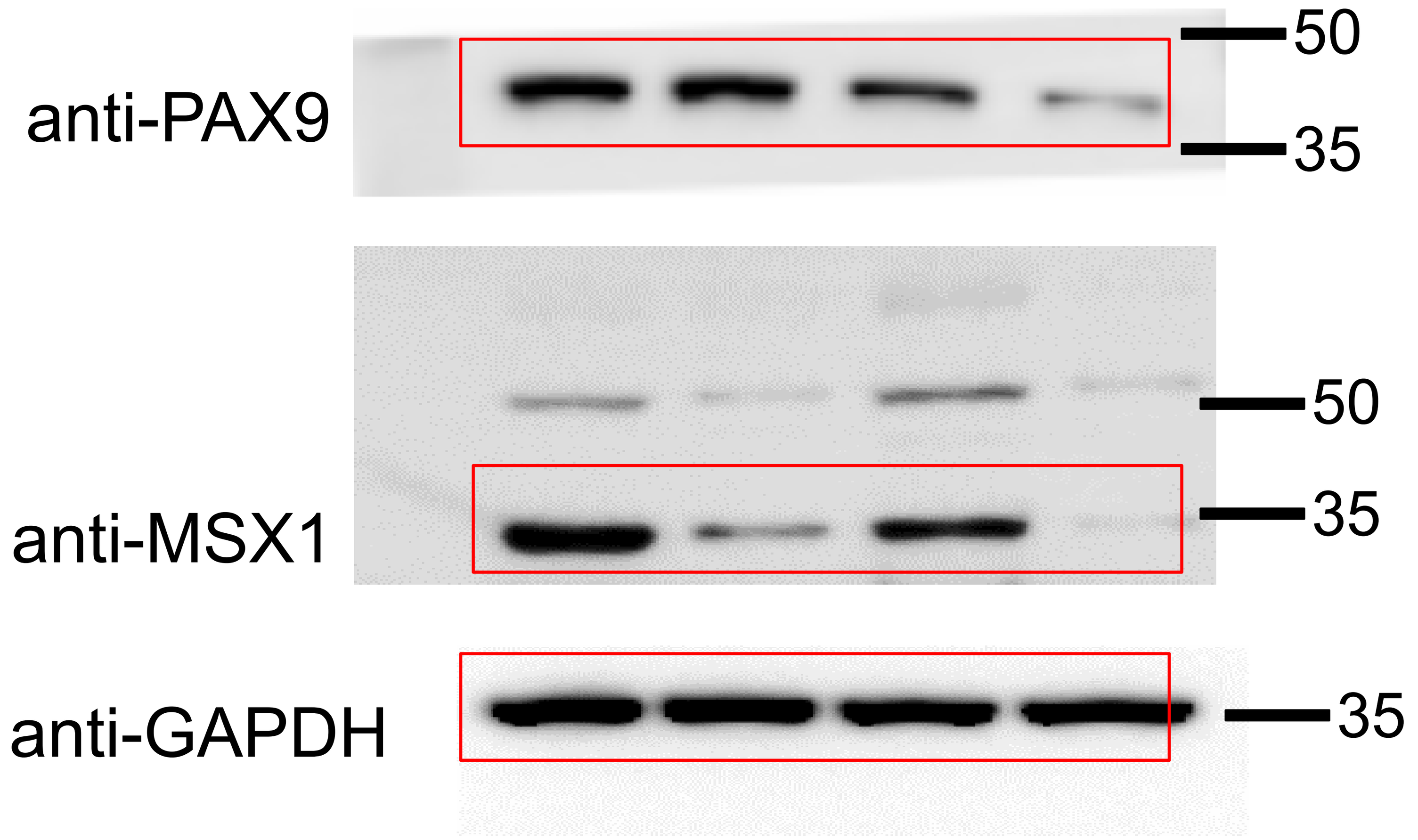

C

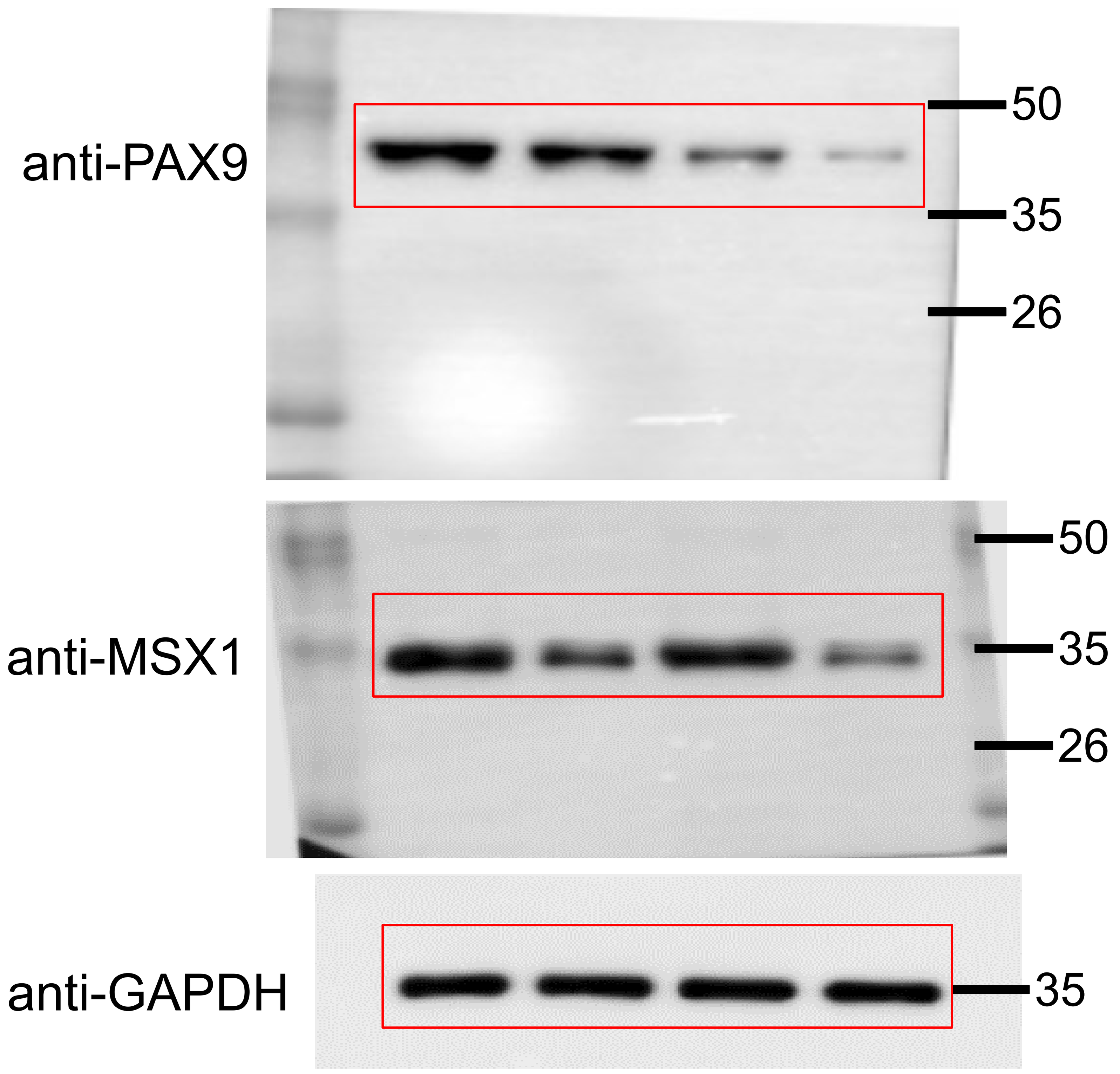

Uncropped blots of Fig. 2

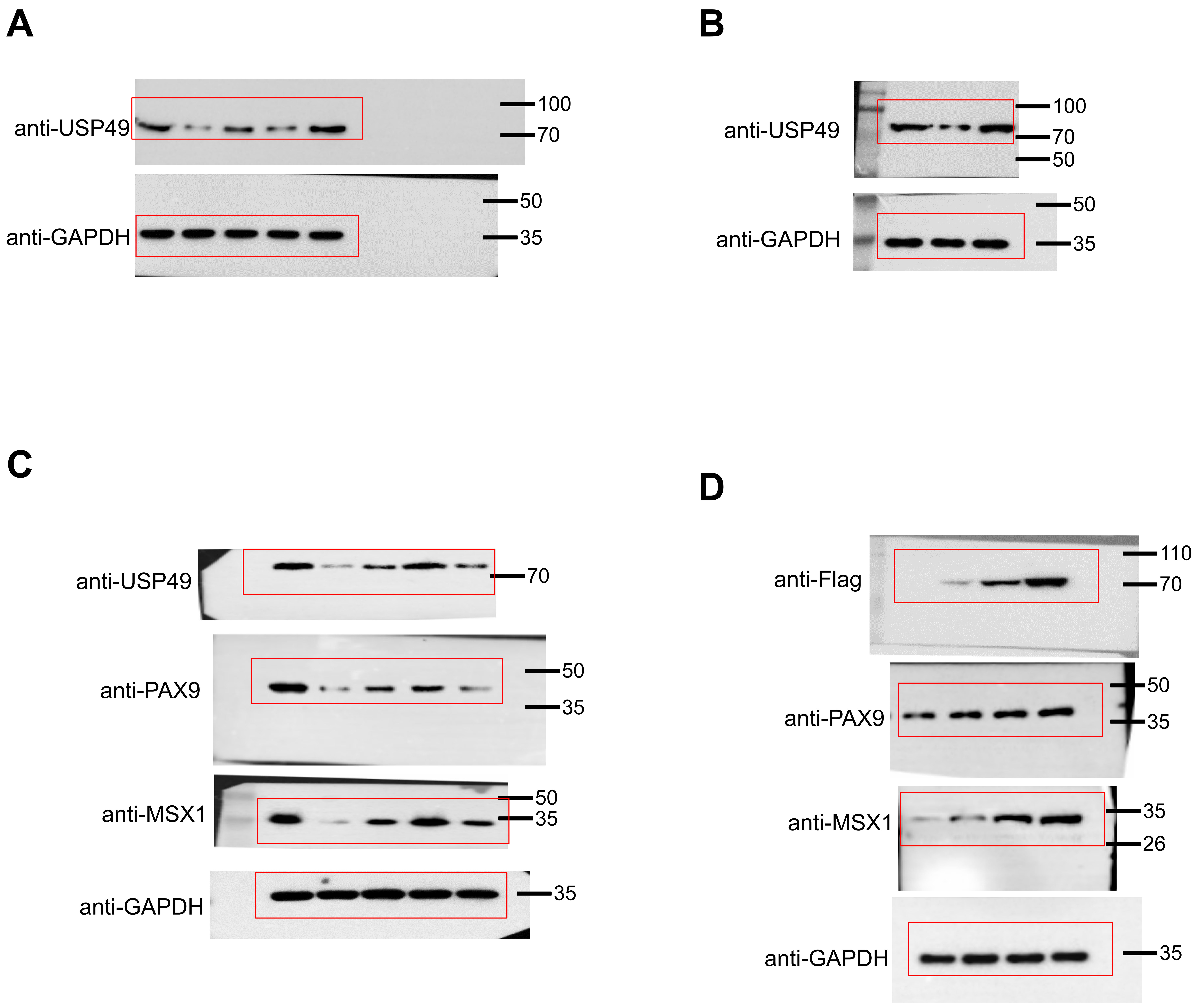

Uncropped blots of Fig. 2

E

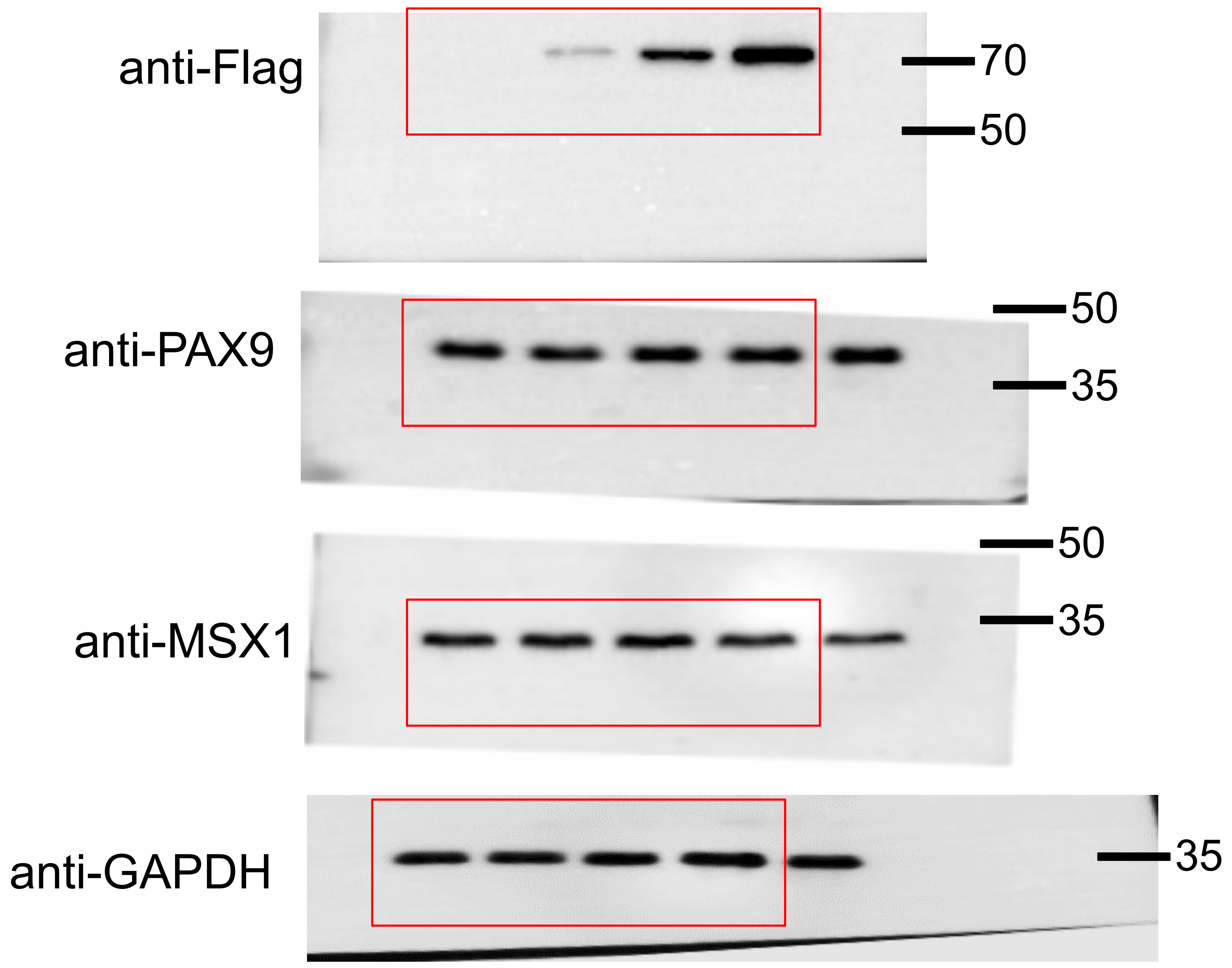

F

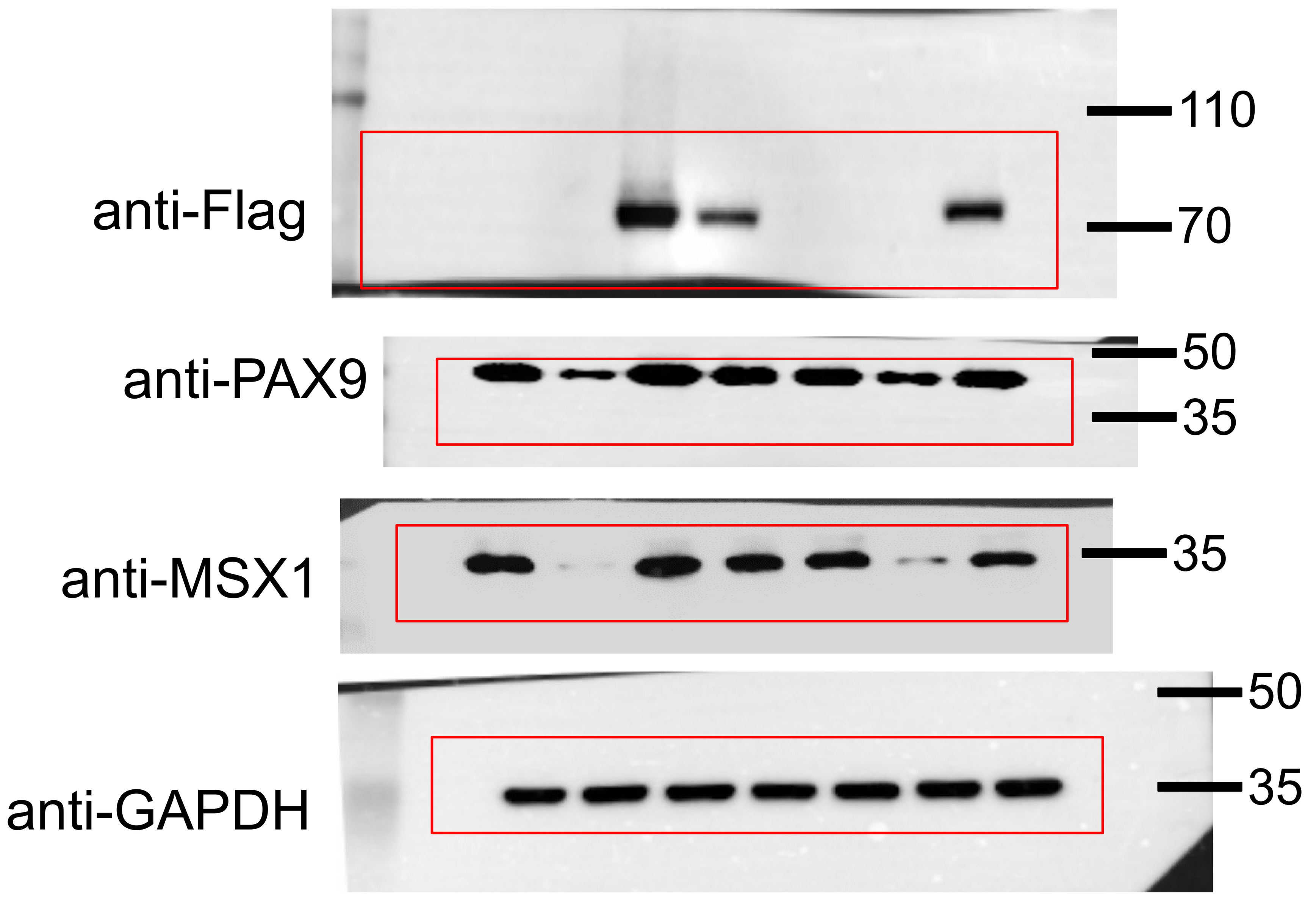

Uncropped blots of Fig. 3

A

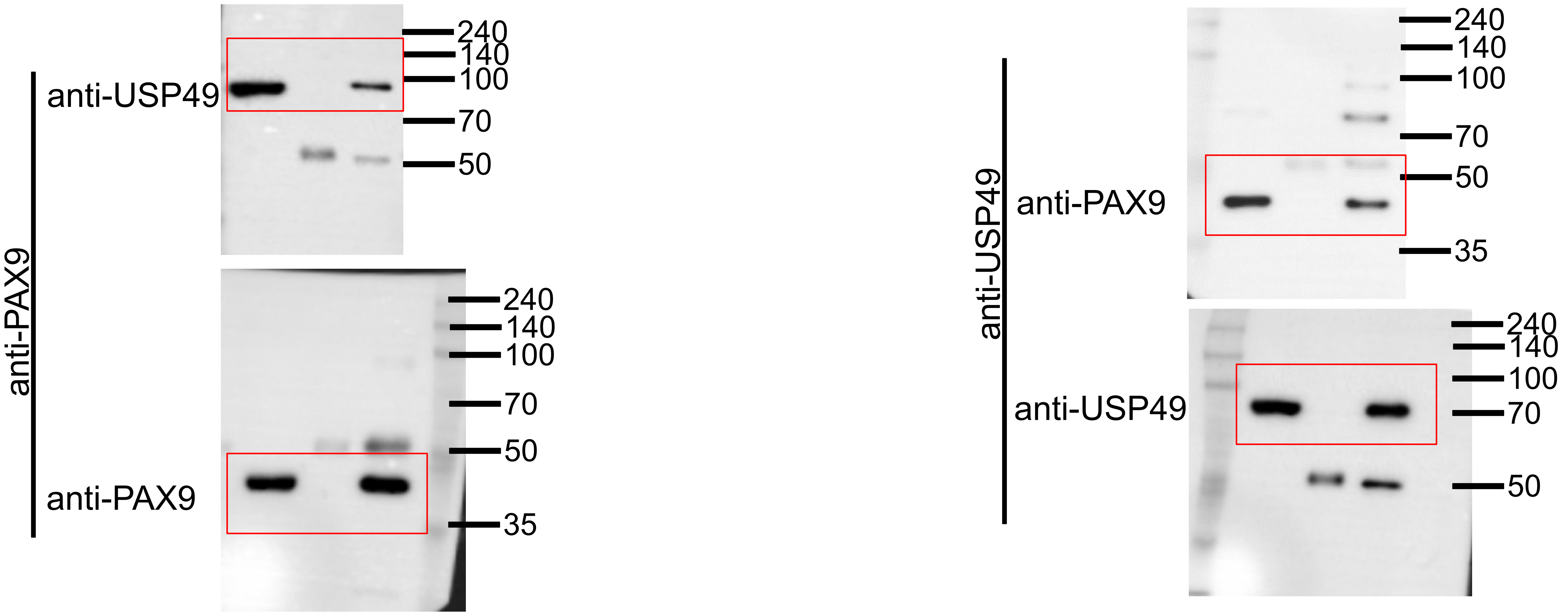

B

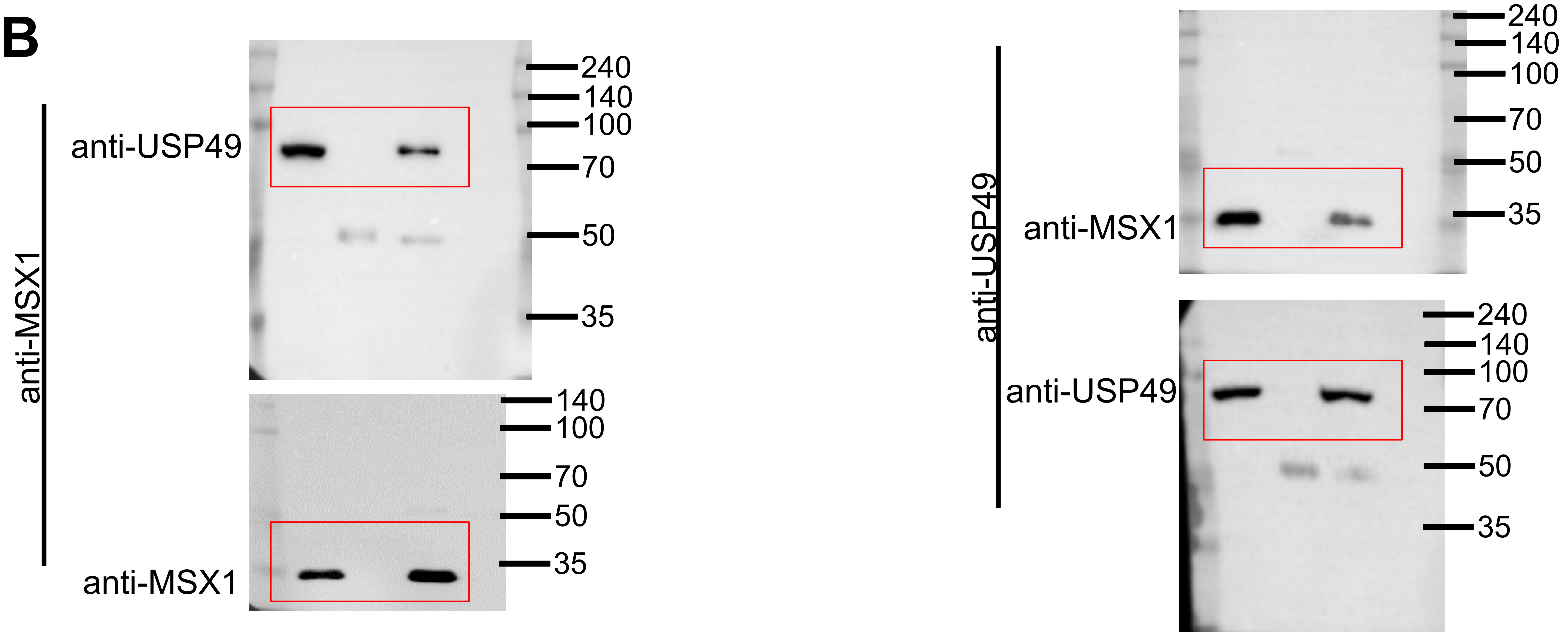

Uncropped blots of Fig. 3

C

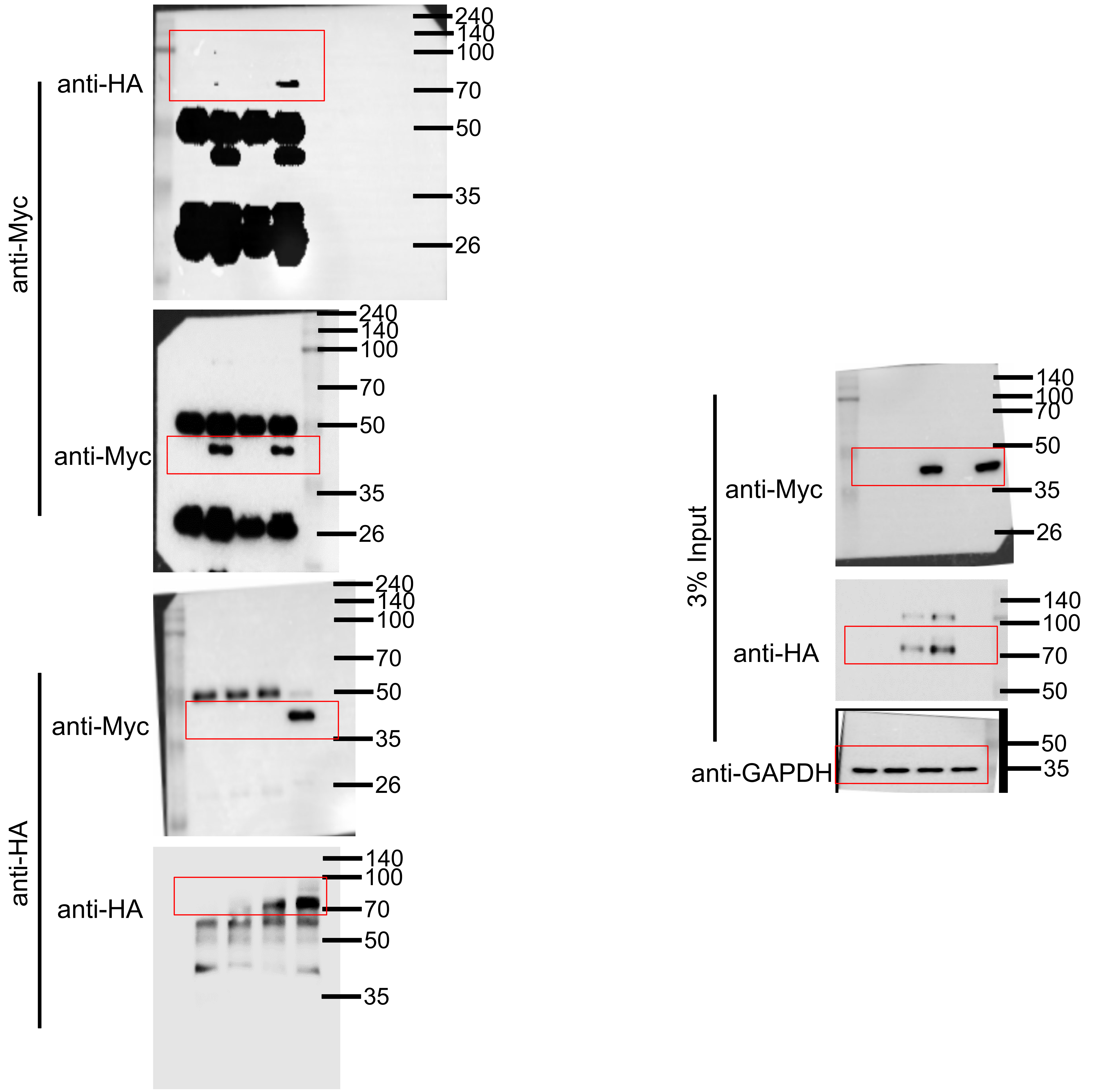

Uncropped blots of Fig. 3

D

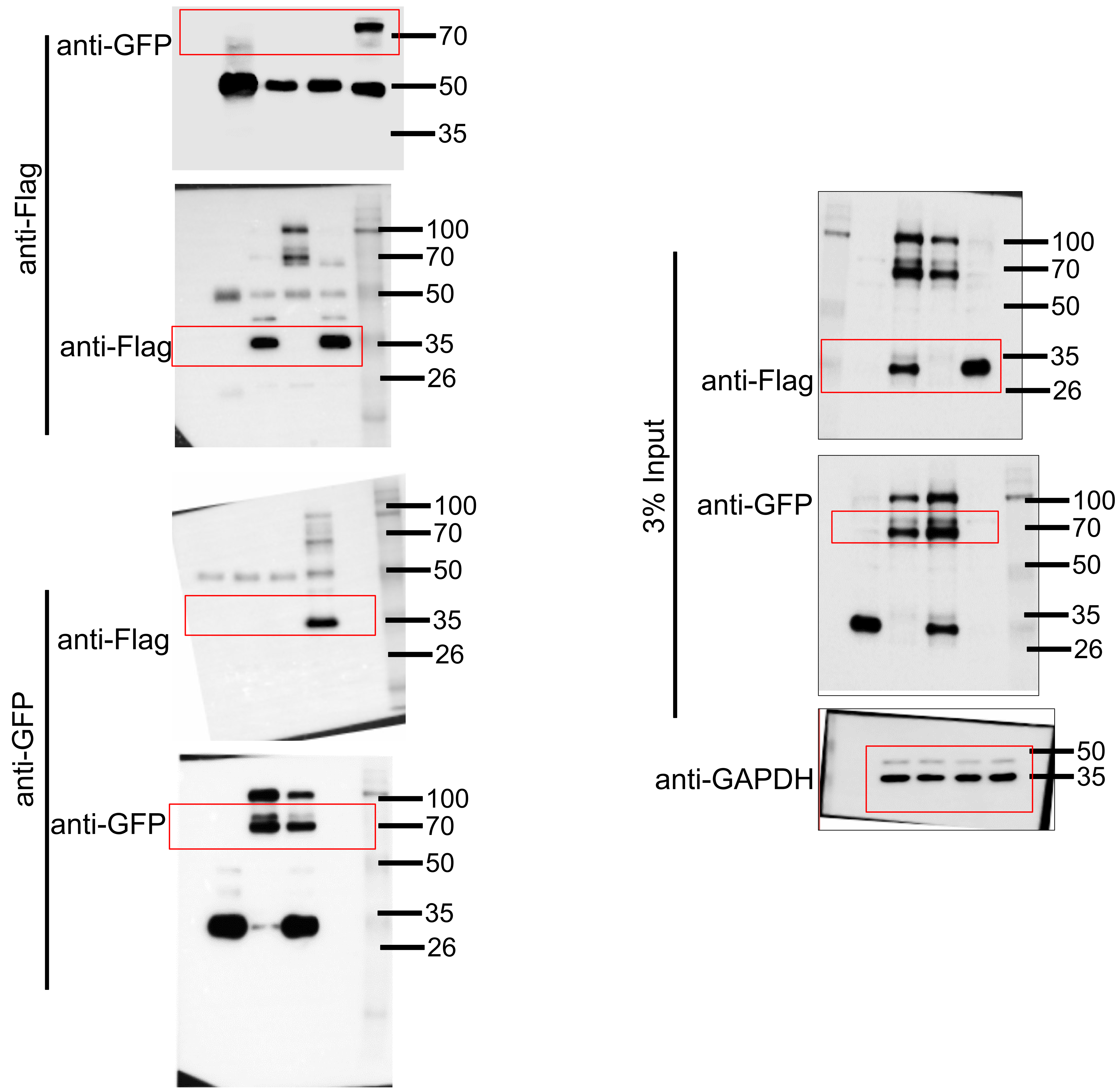

Uncropped blots of Fig. 3

G

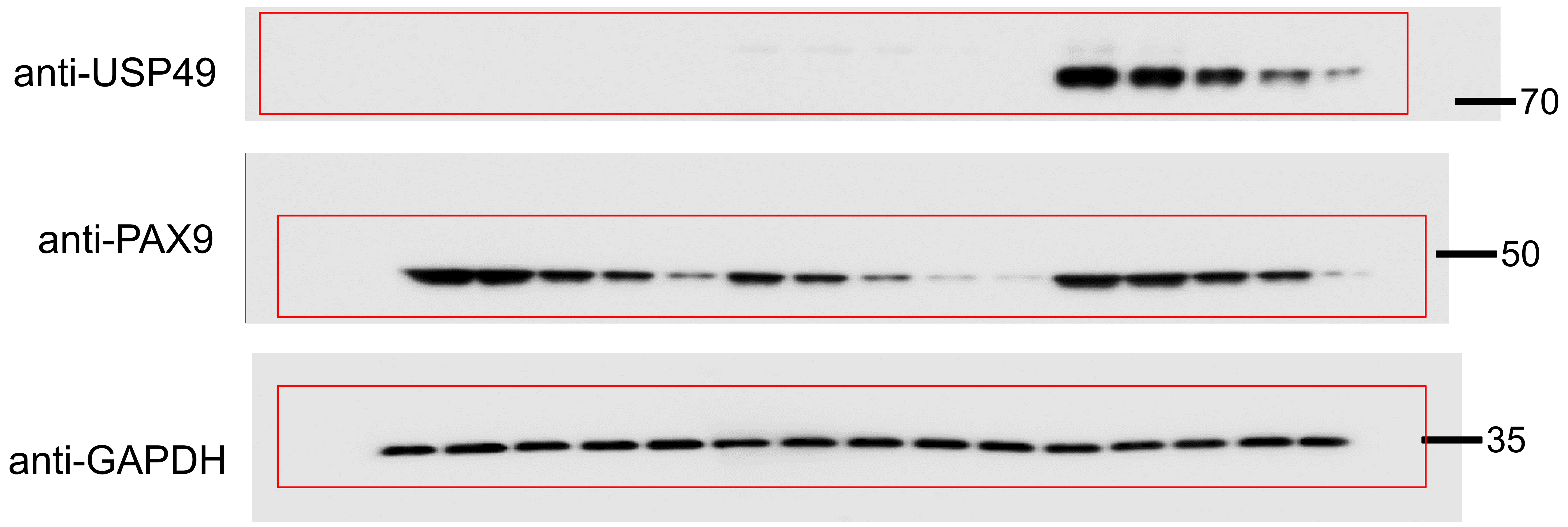

H

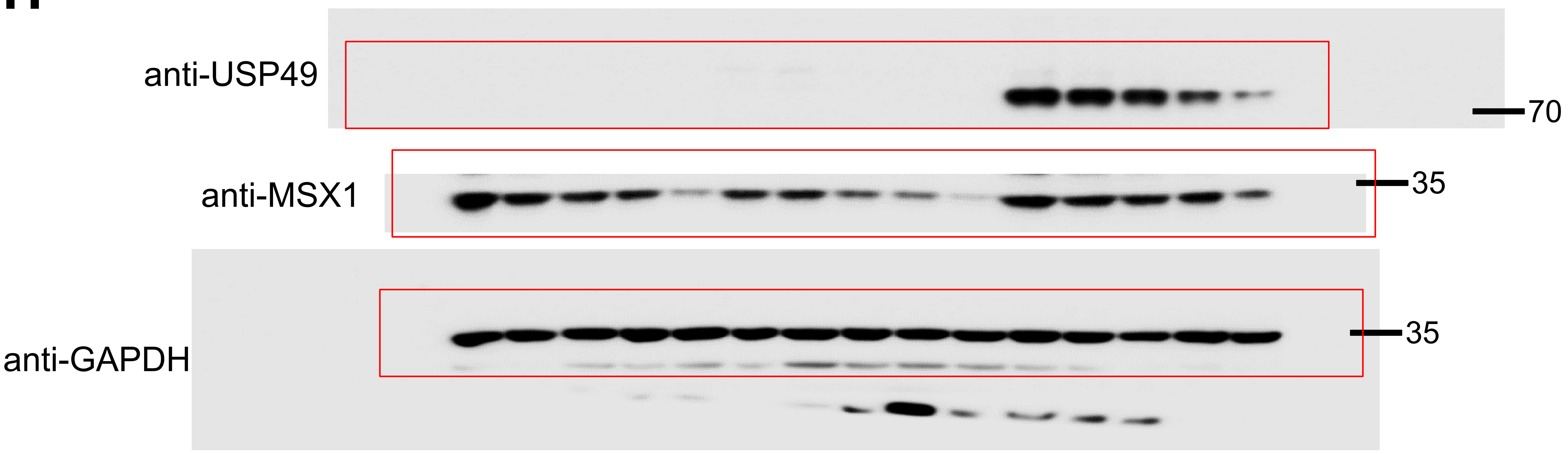

Uncropped blots of Fig. 3

I

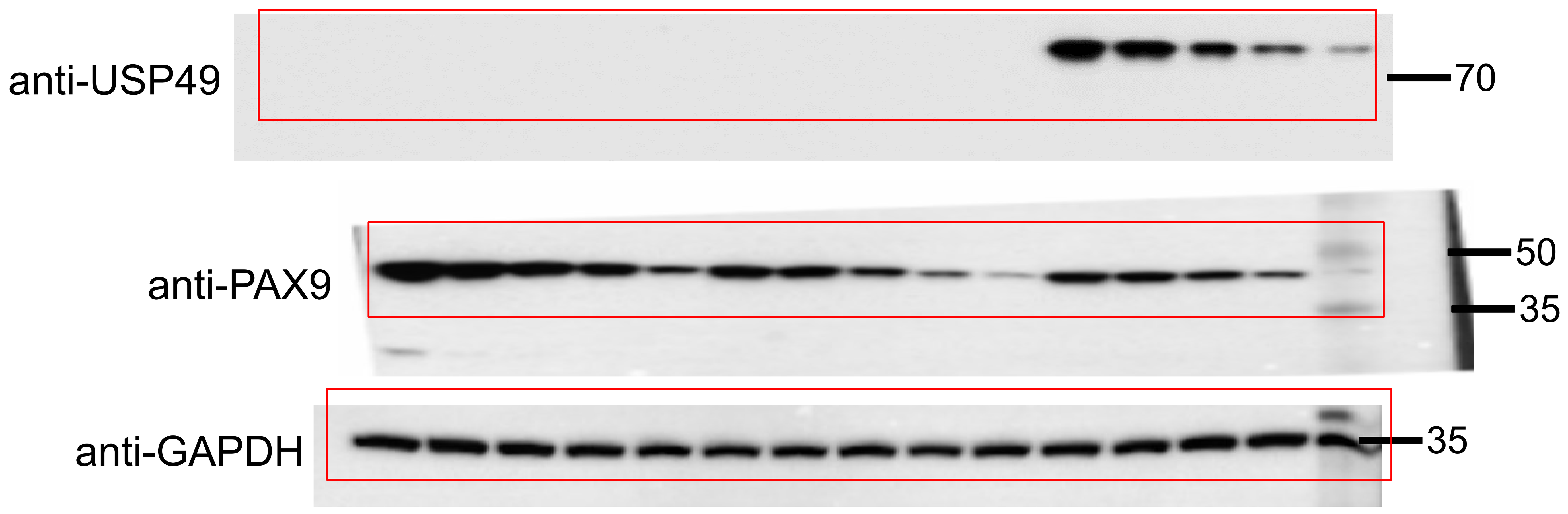

J

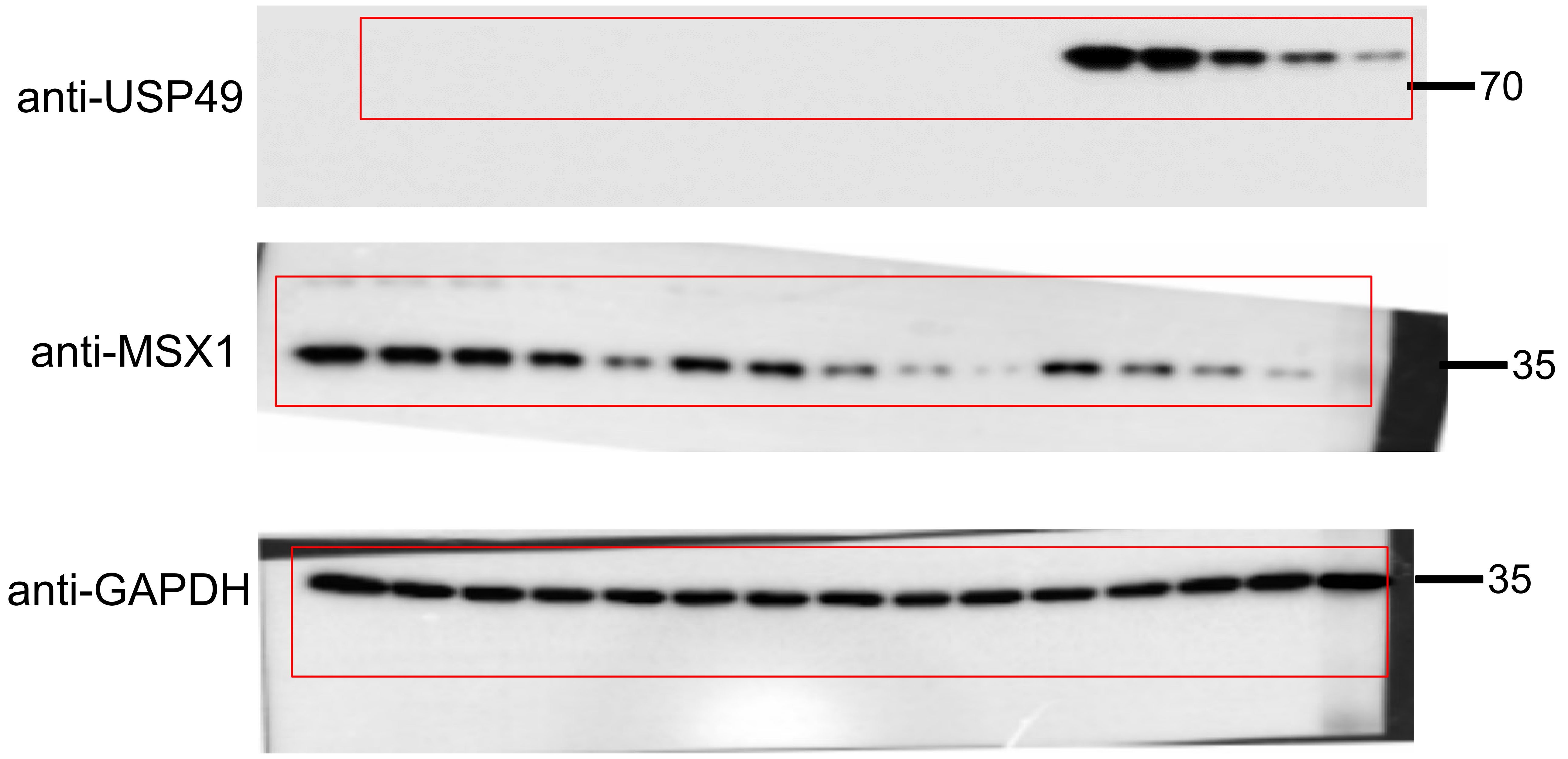

Uncropped blots of Fig. 4

A

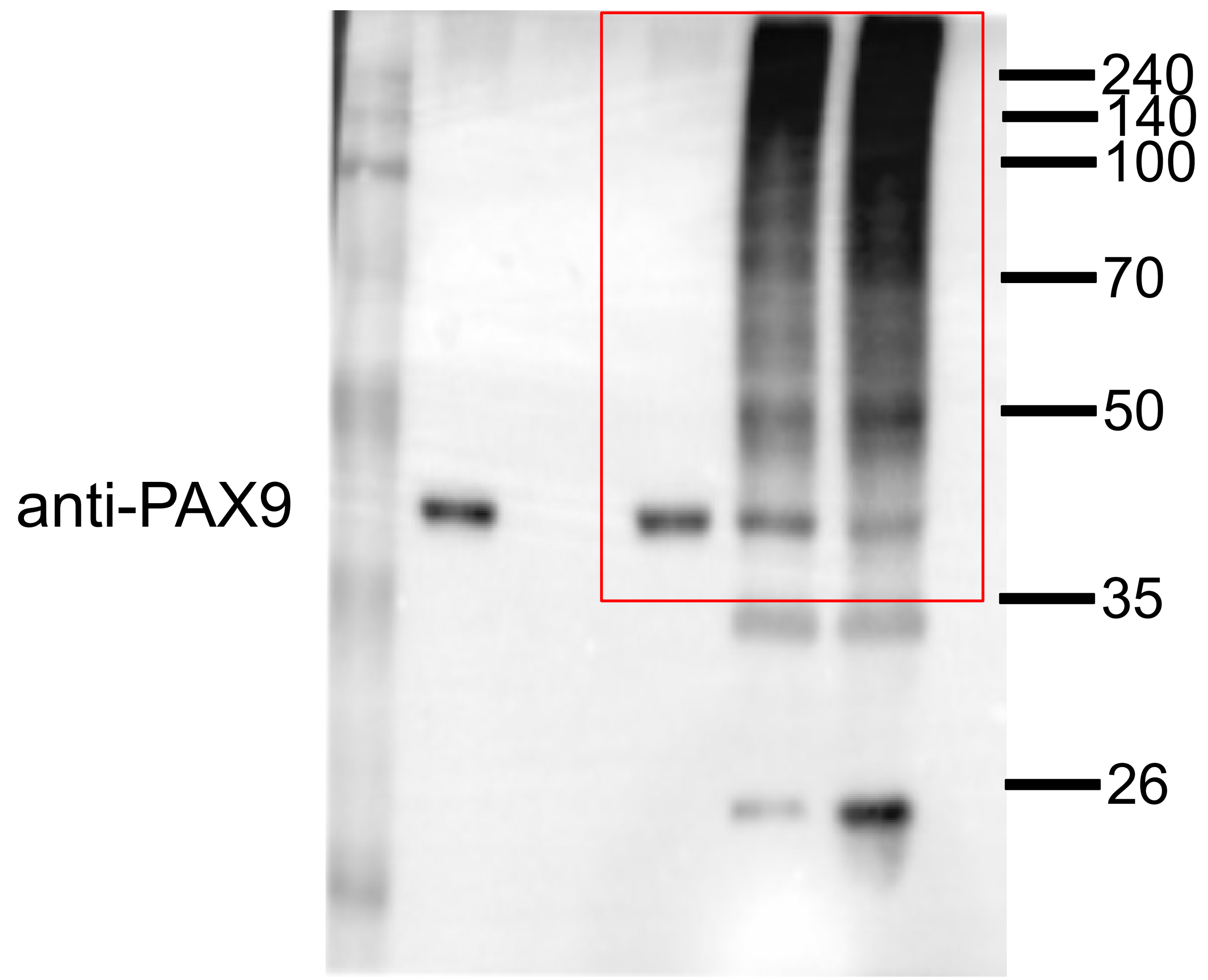

B

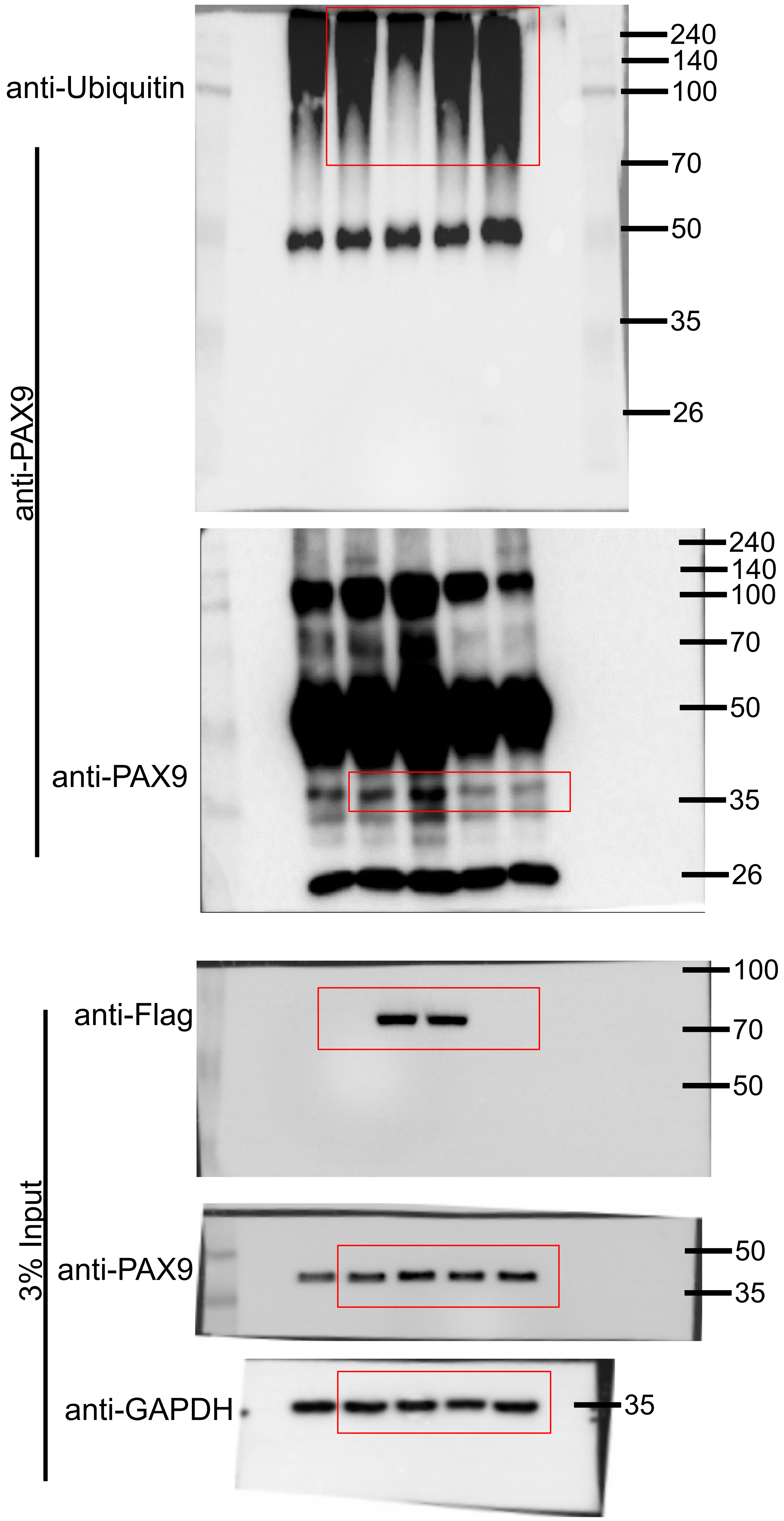

Uncropped blots of Fig. 4

C

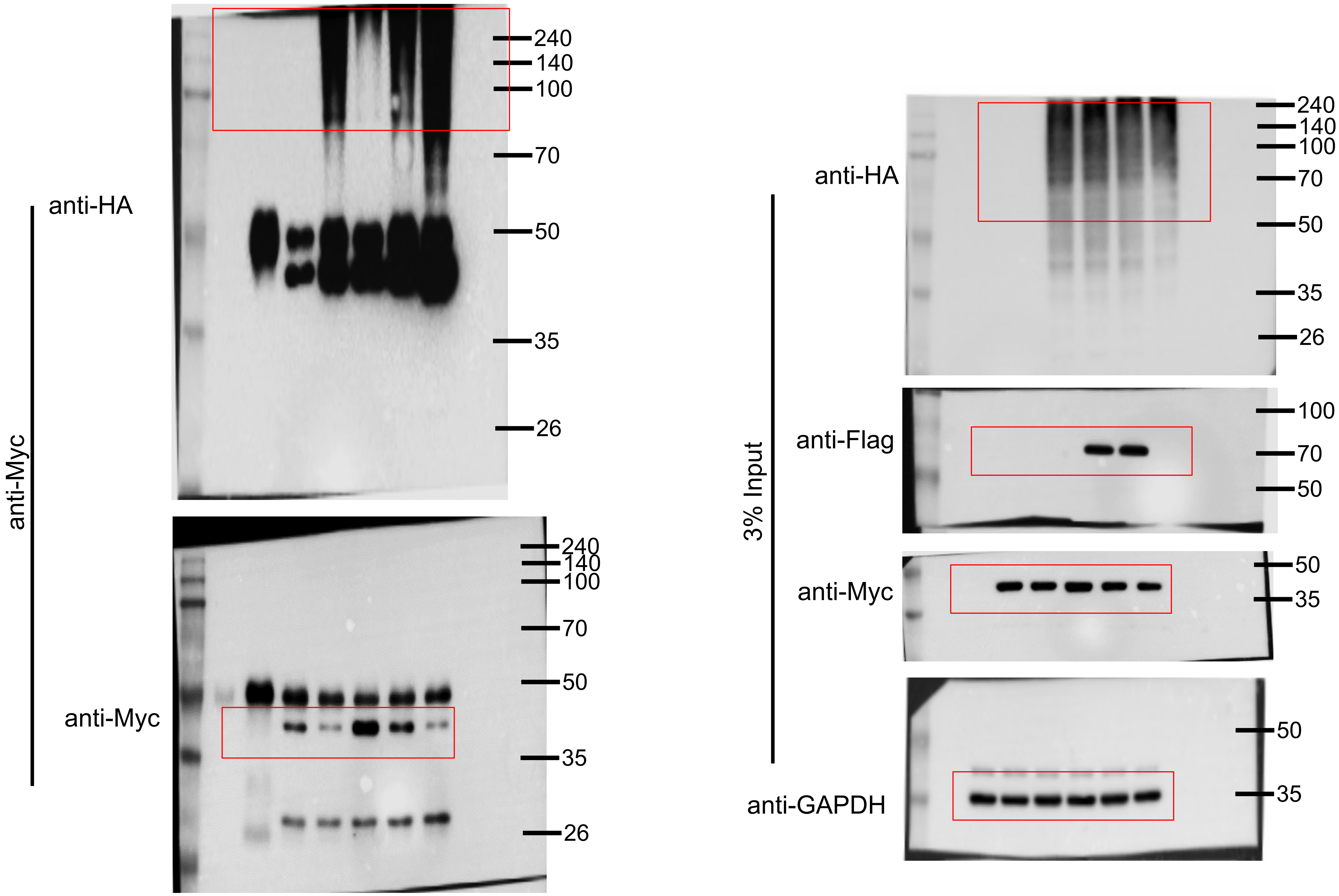

Uncropped blots of Fig. 4

D

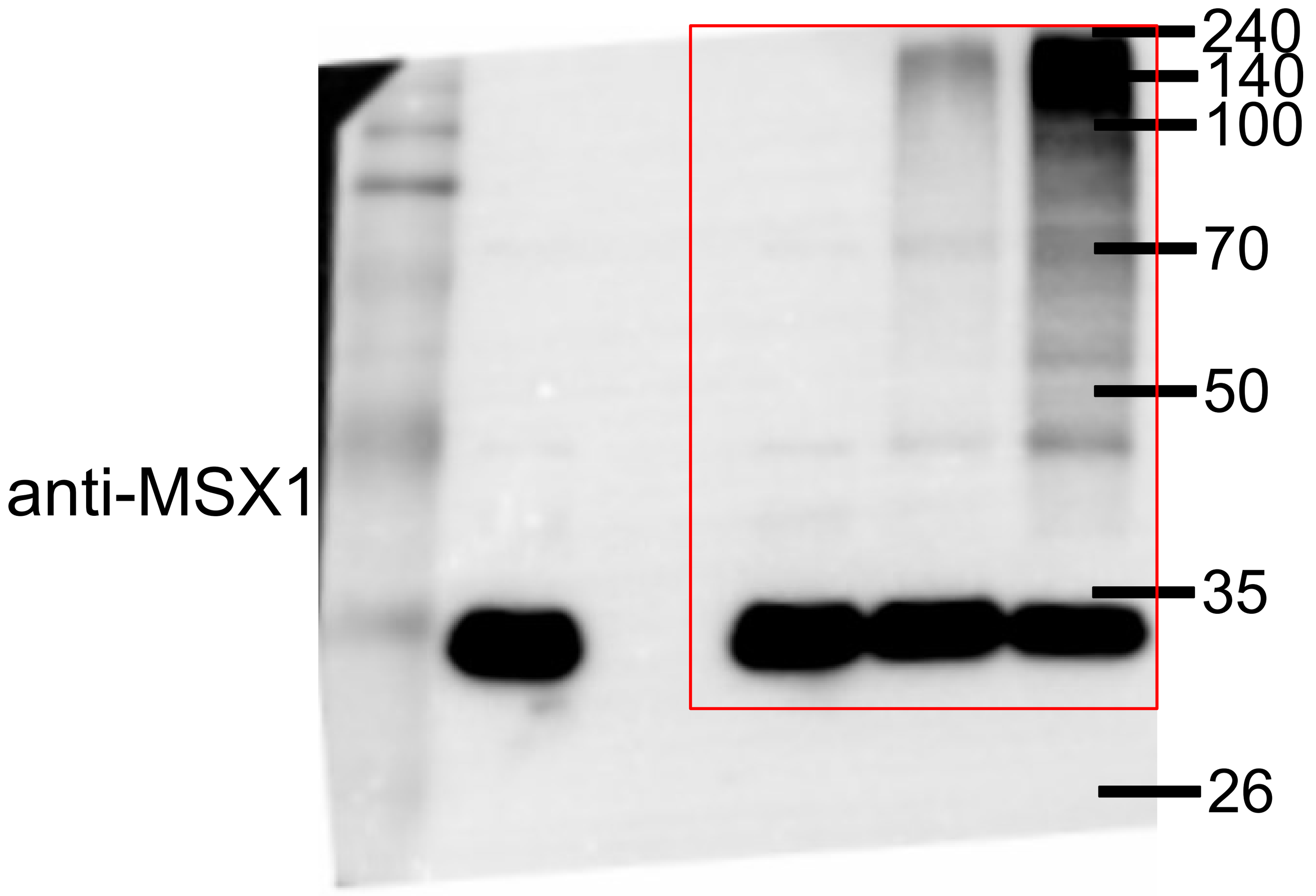

E

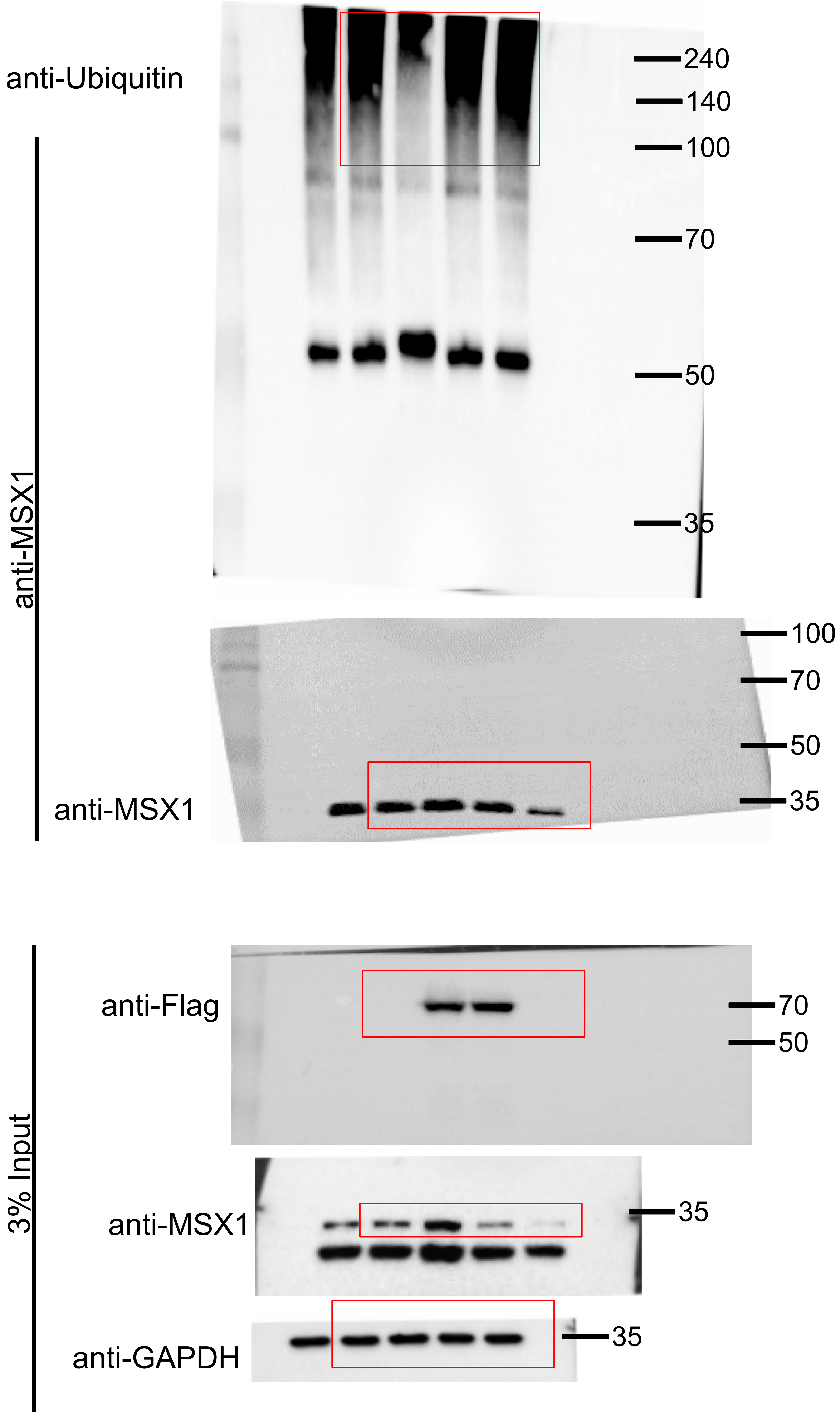

Uncropped blots of Fig. 4

F

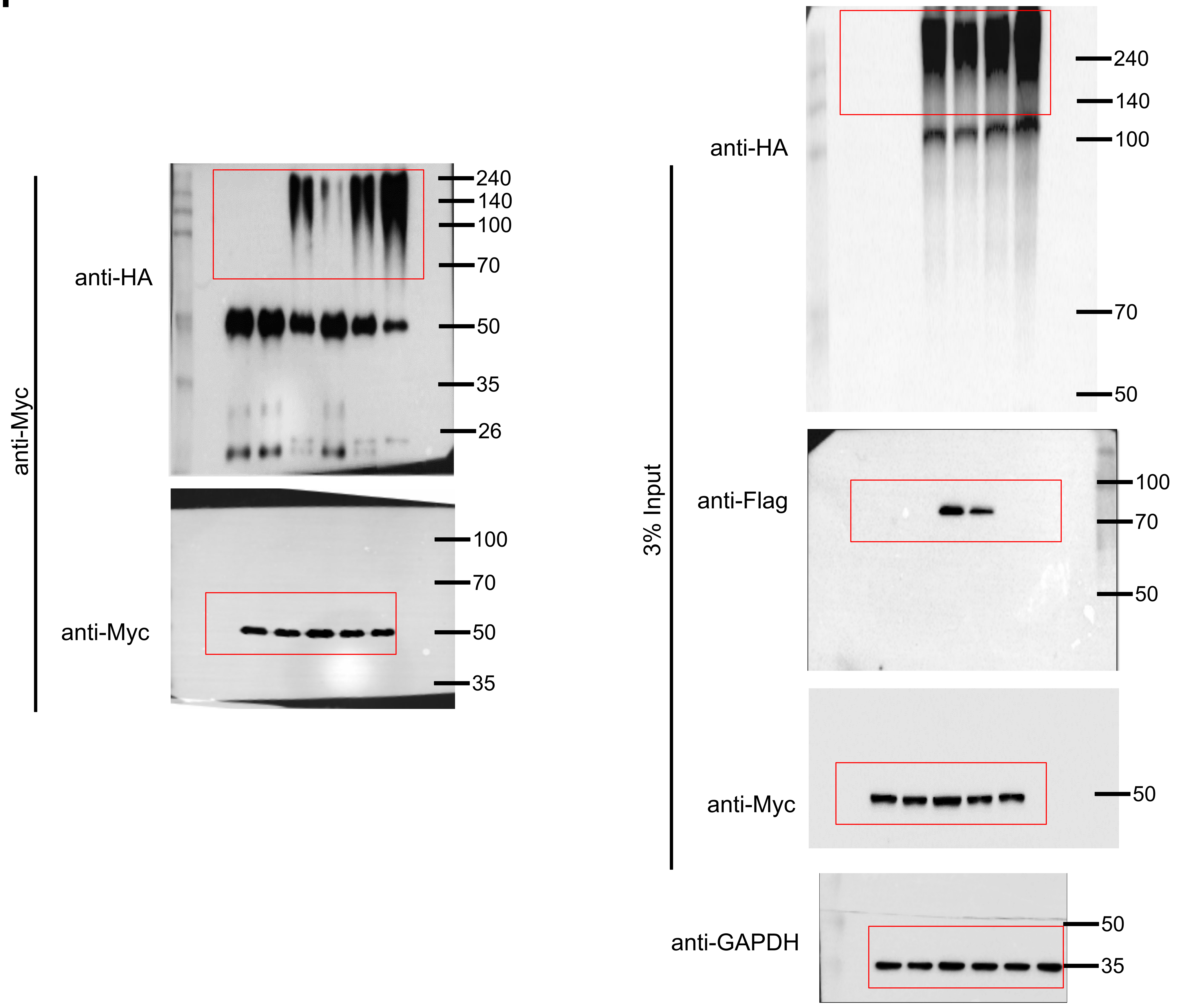

Uncropped blots of Fig. 5

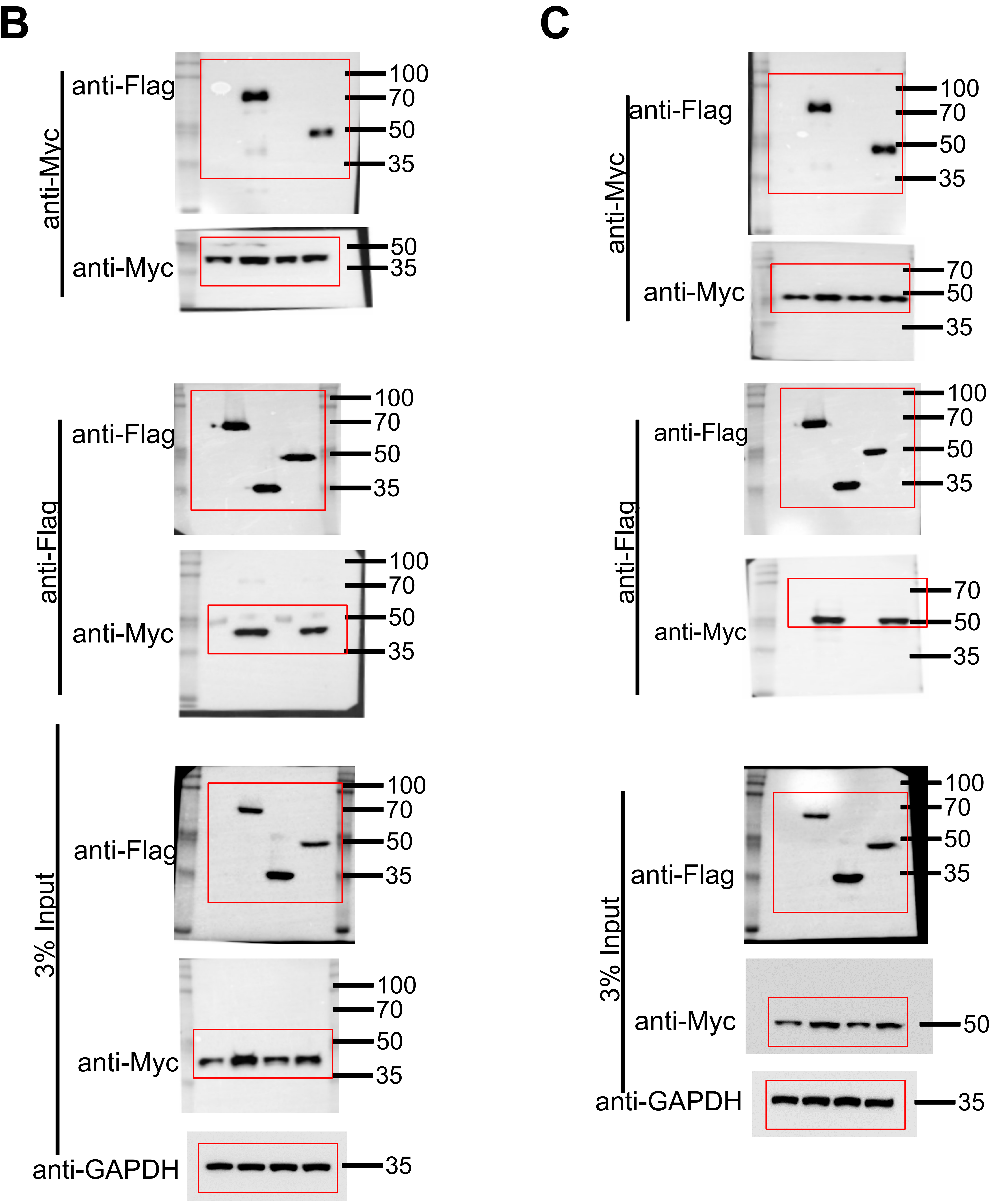

Uncropped blots of Fig. 5

D

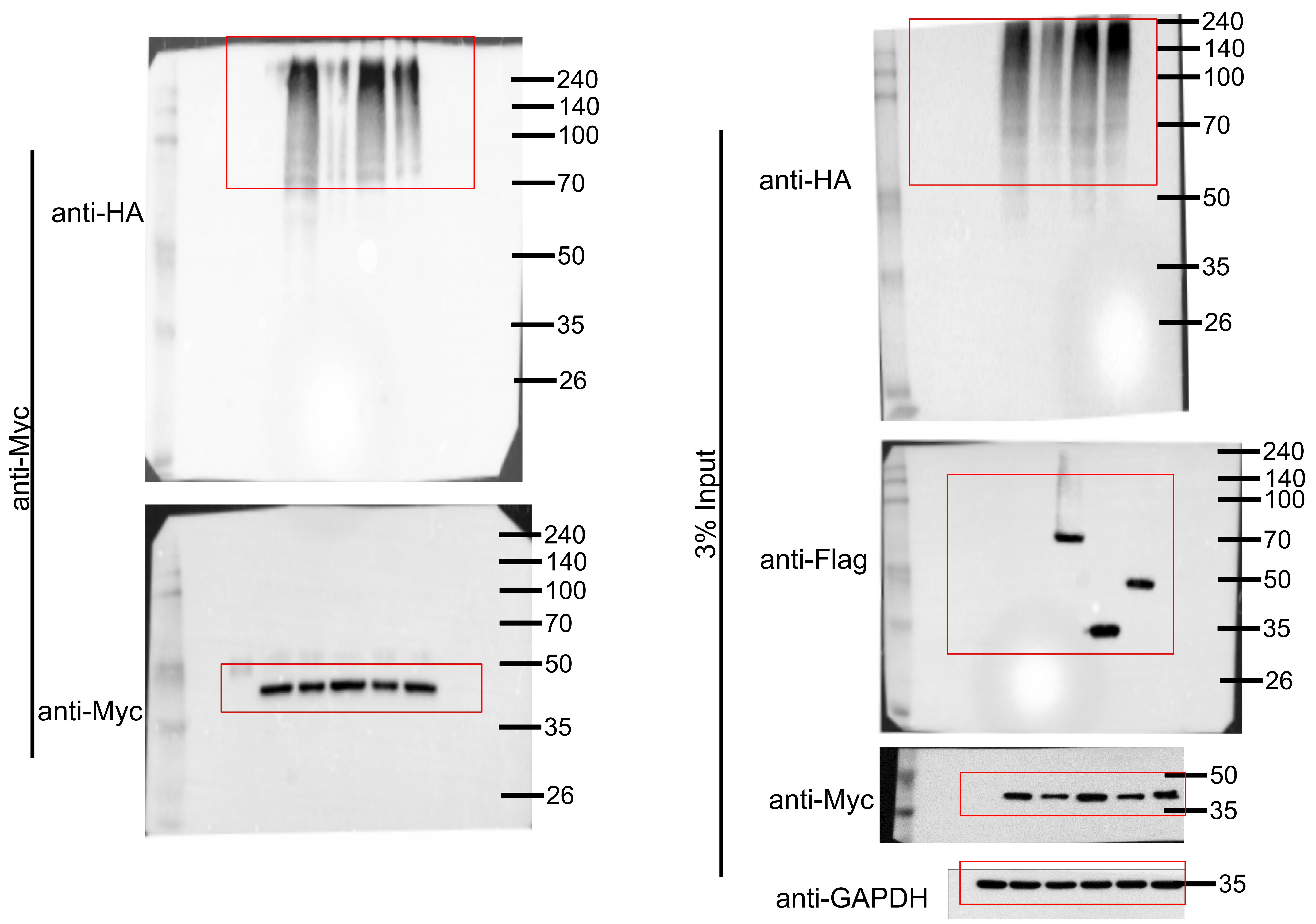

Uncropped blots of Fig. 5

E

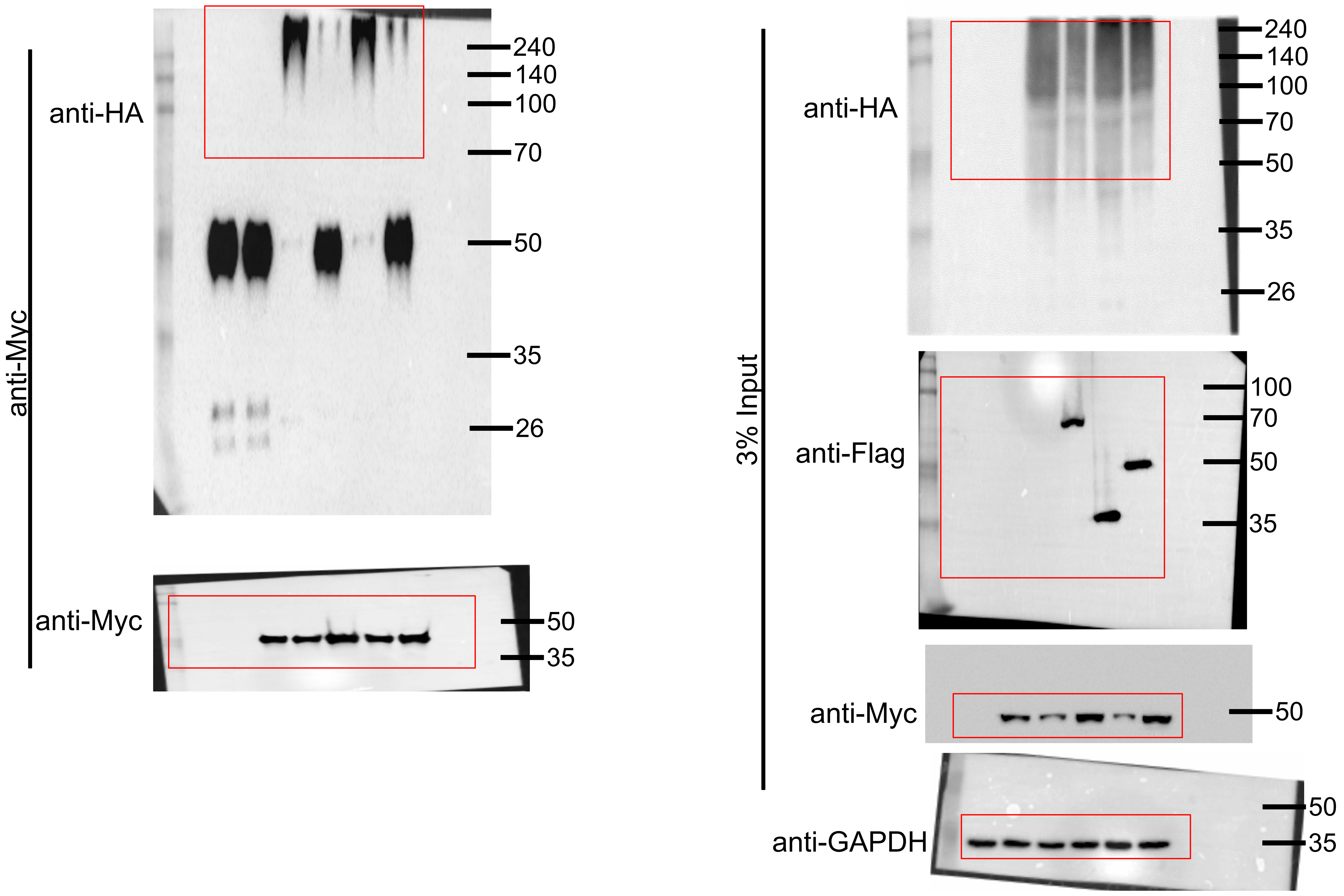

Uncropped blots of Fig. 6

A

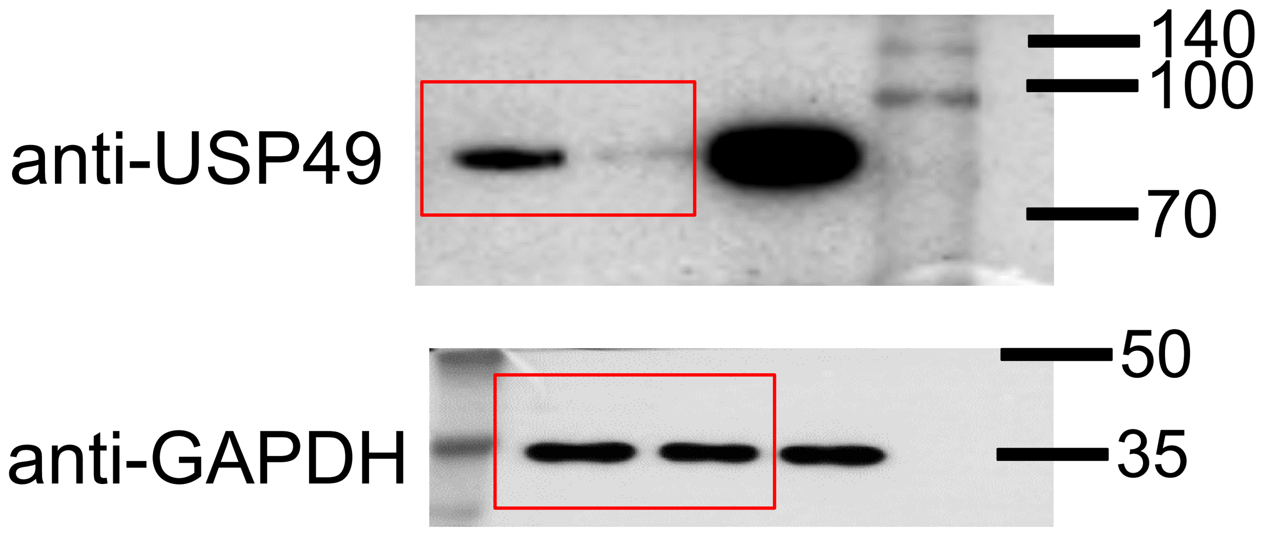

I

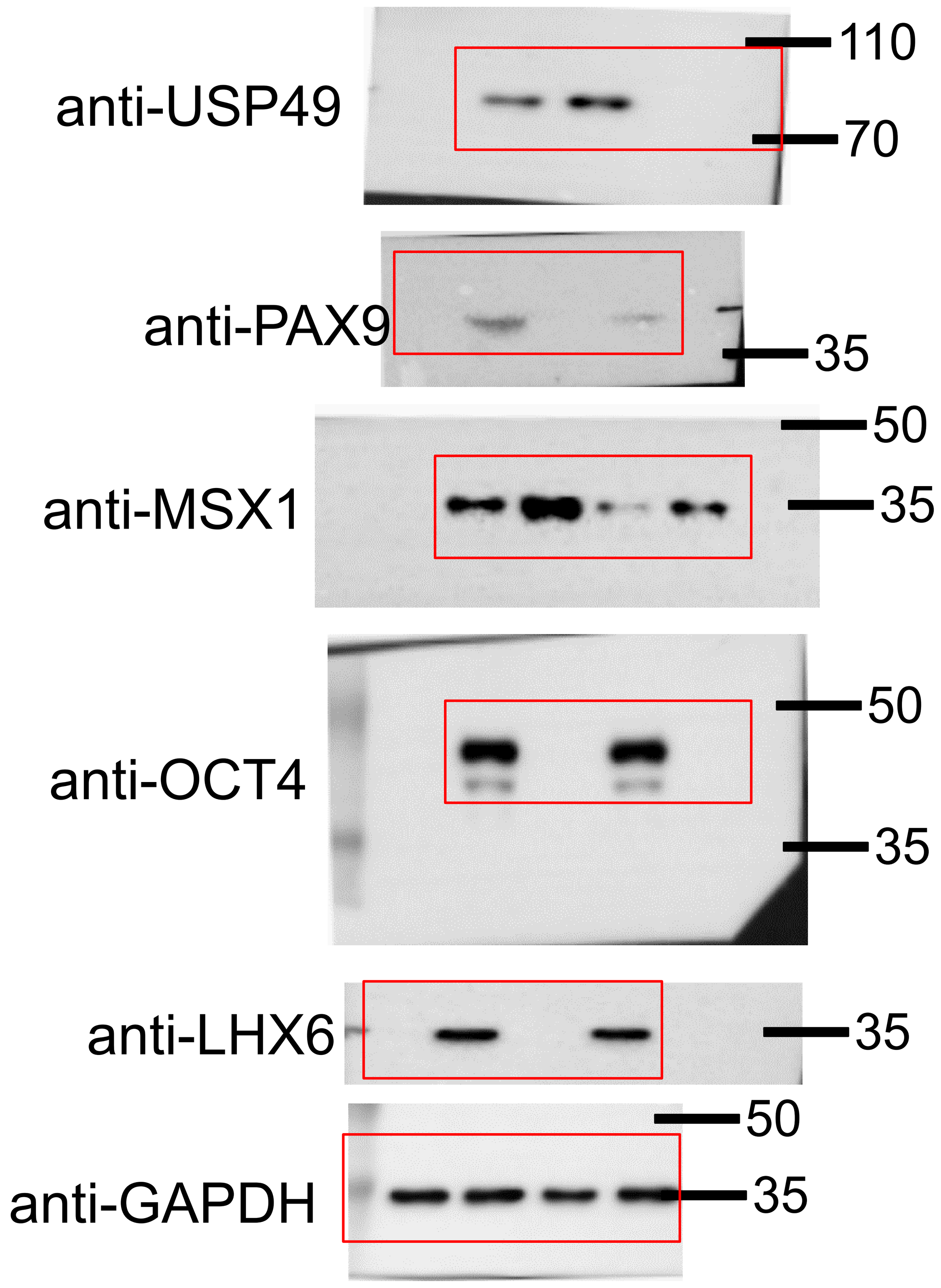

Uncropped blots of Supplementary Fig. S2

A

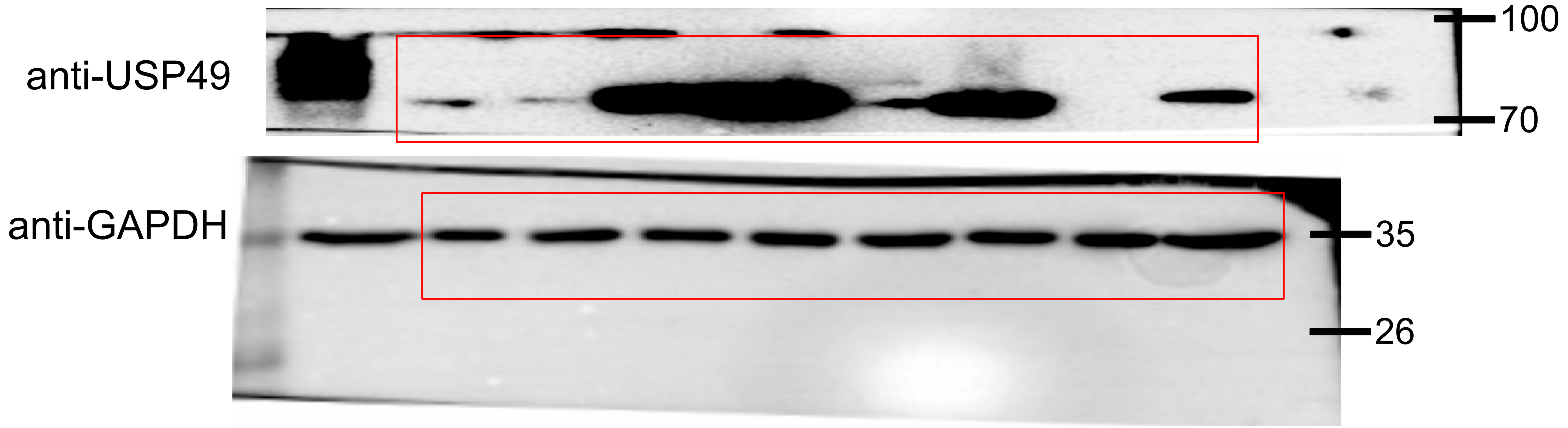

B

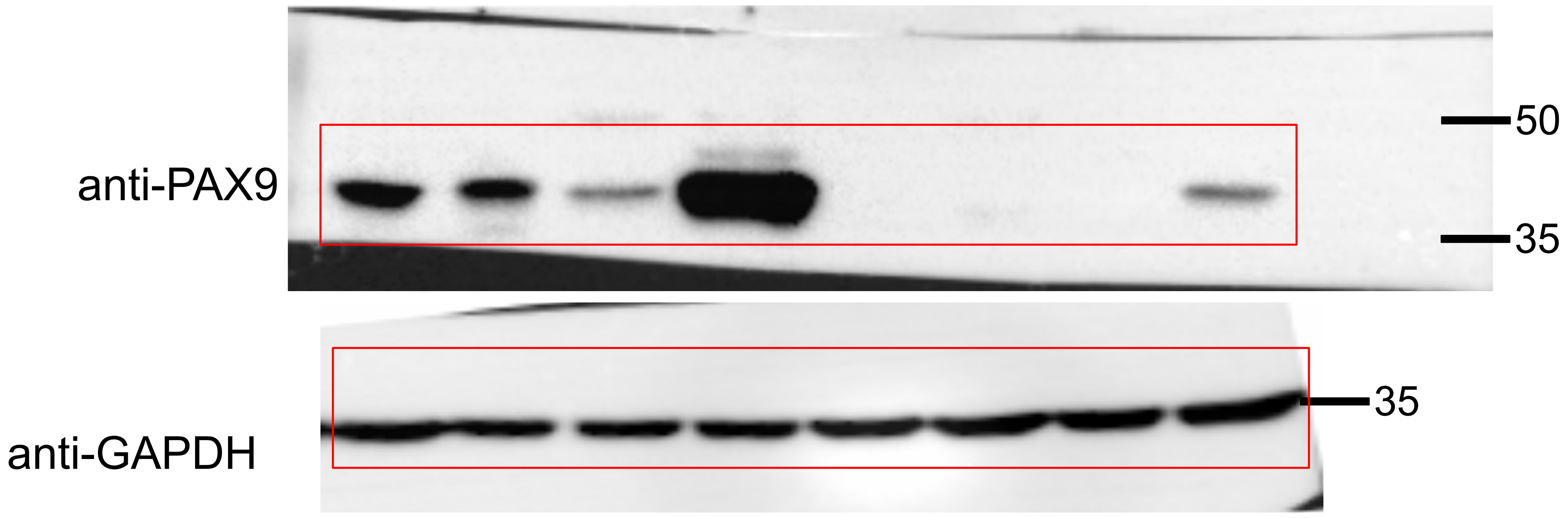

C

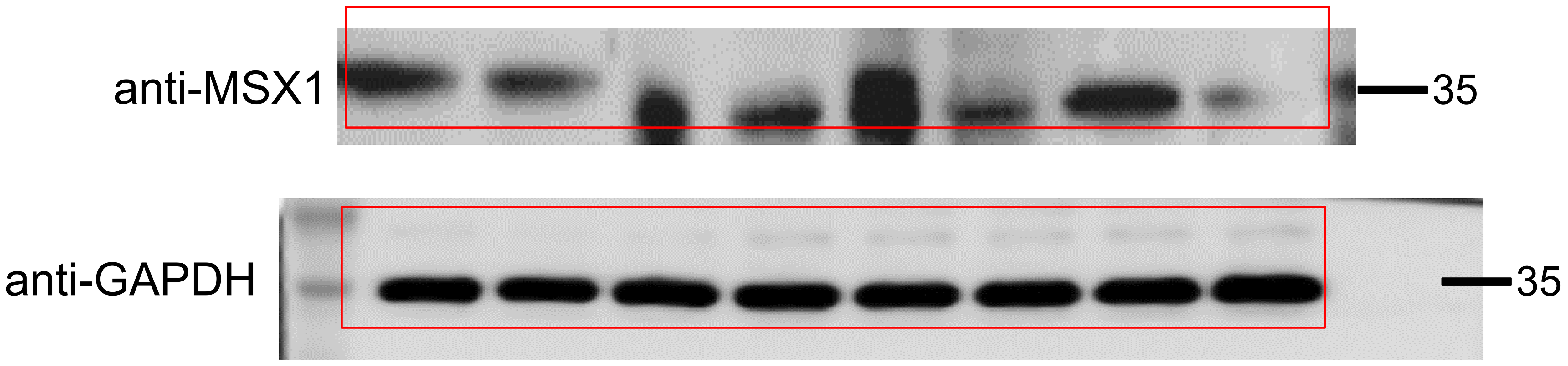

Uncropped blots of Supplementary Fig. S3

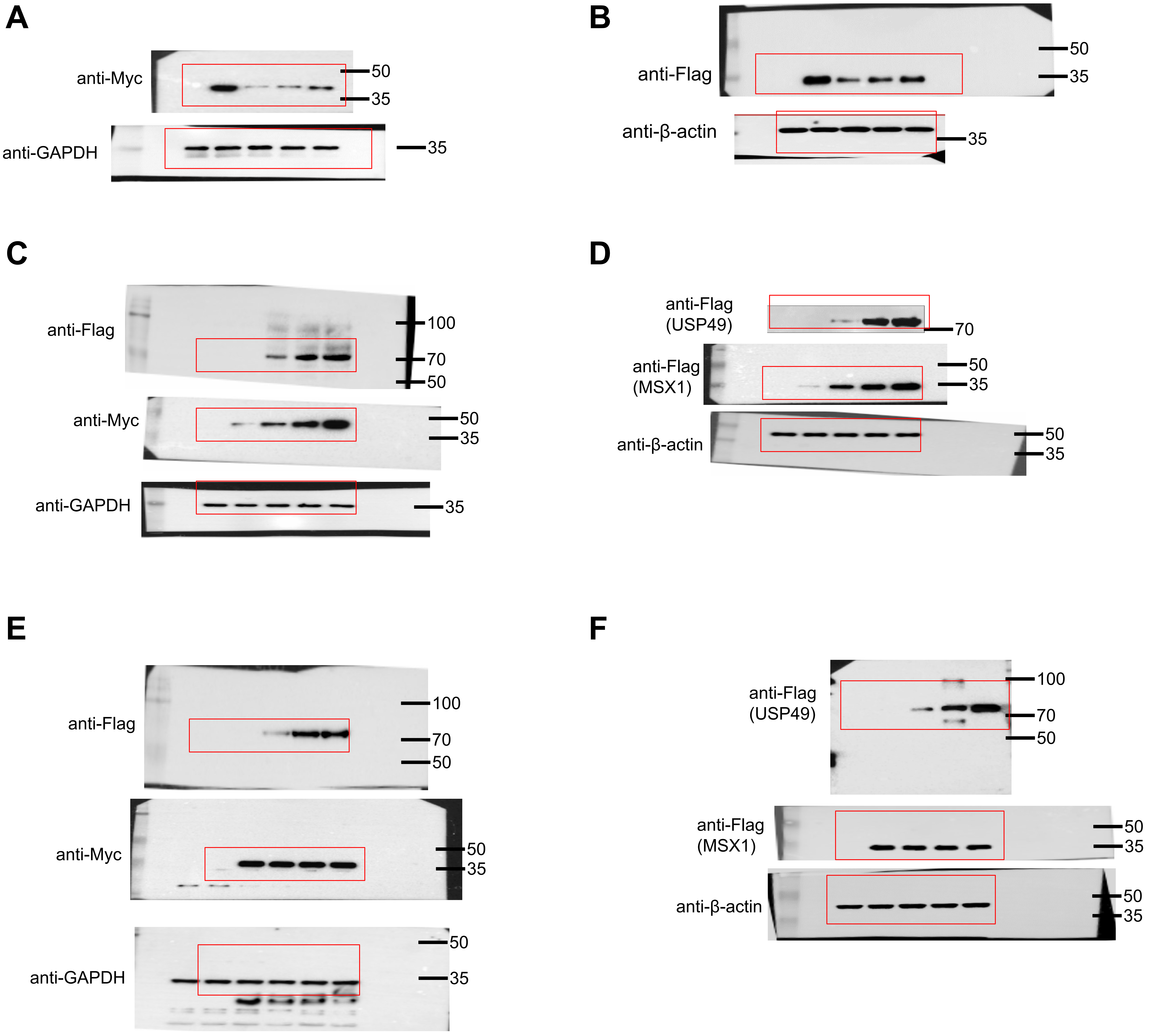

Uncropped blots of Supplementary Fig. S4

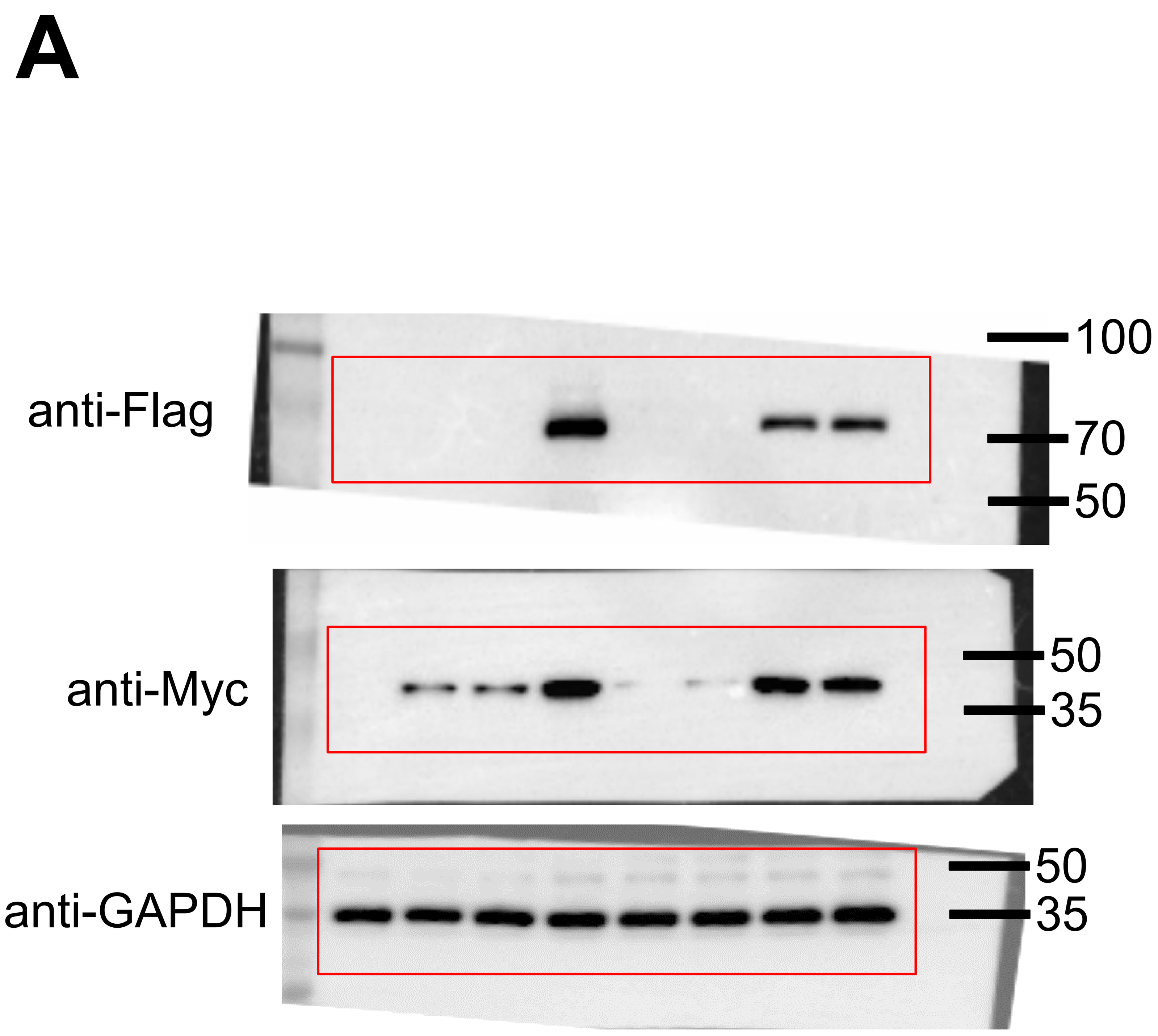

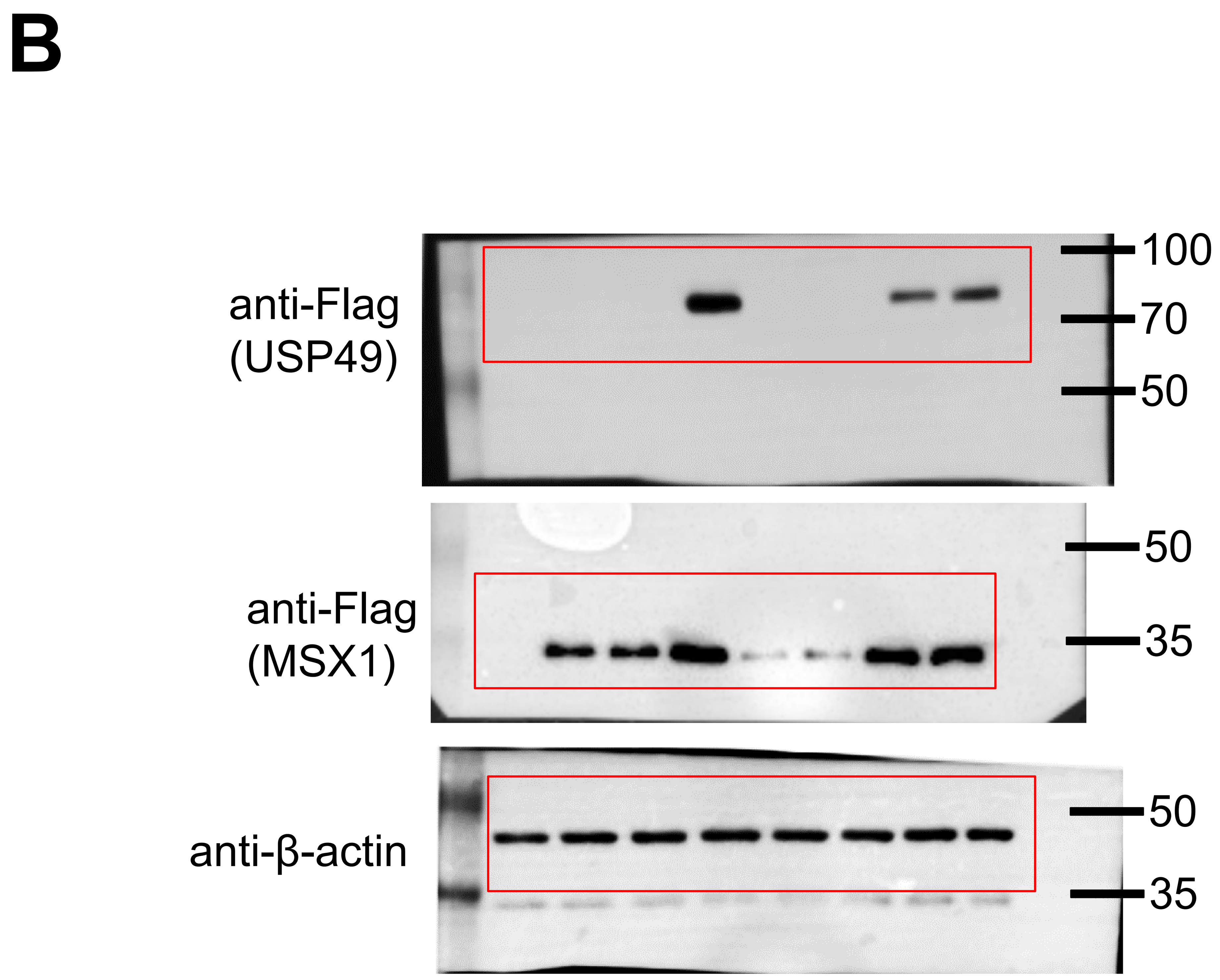

Uncropped blots of Supplementary Fig. S5

A

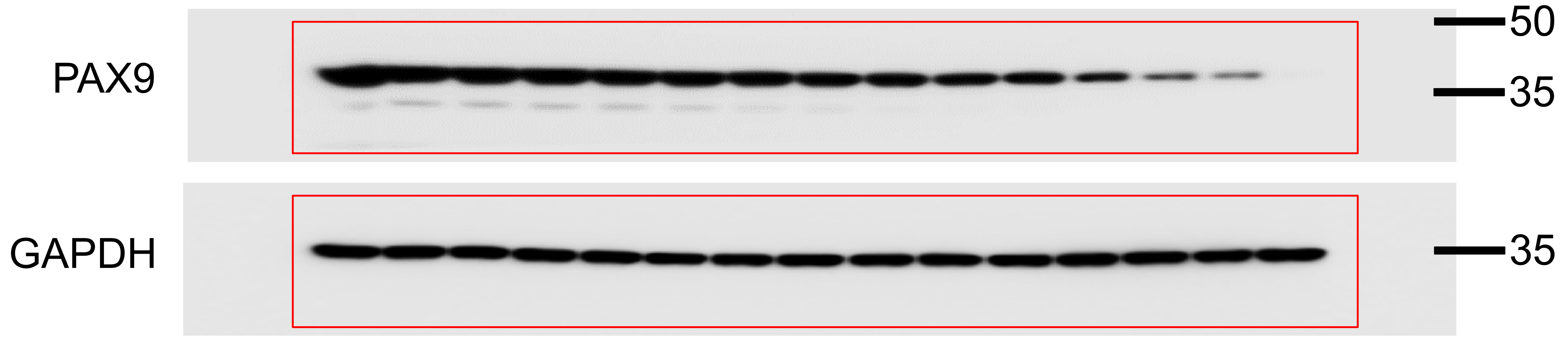

B

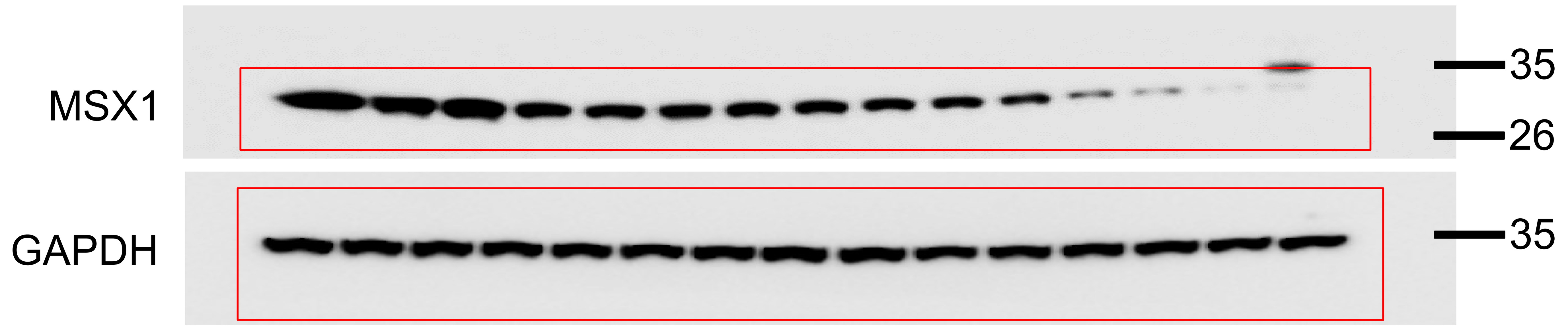

C

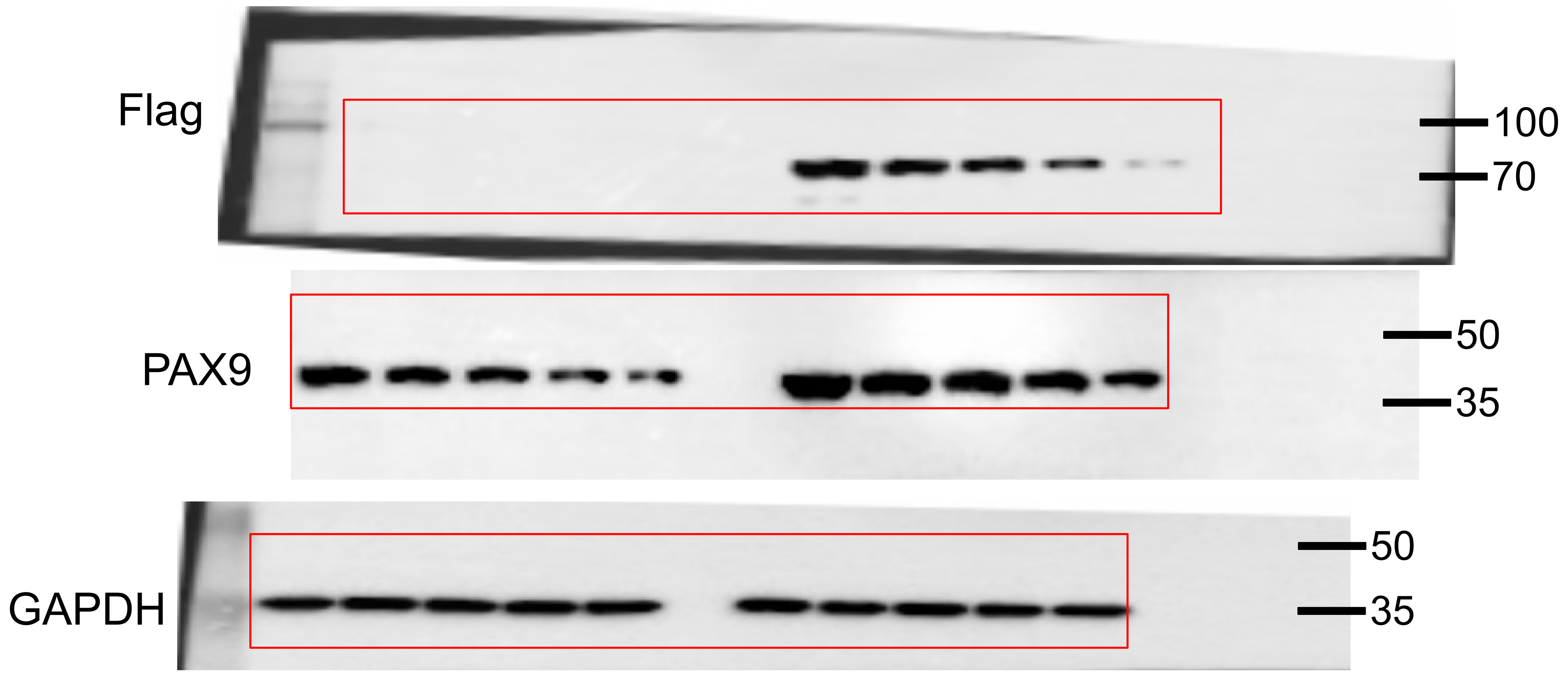

D

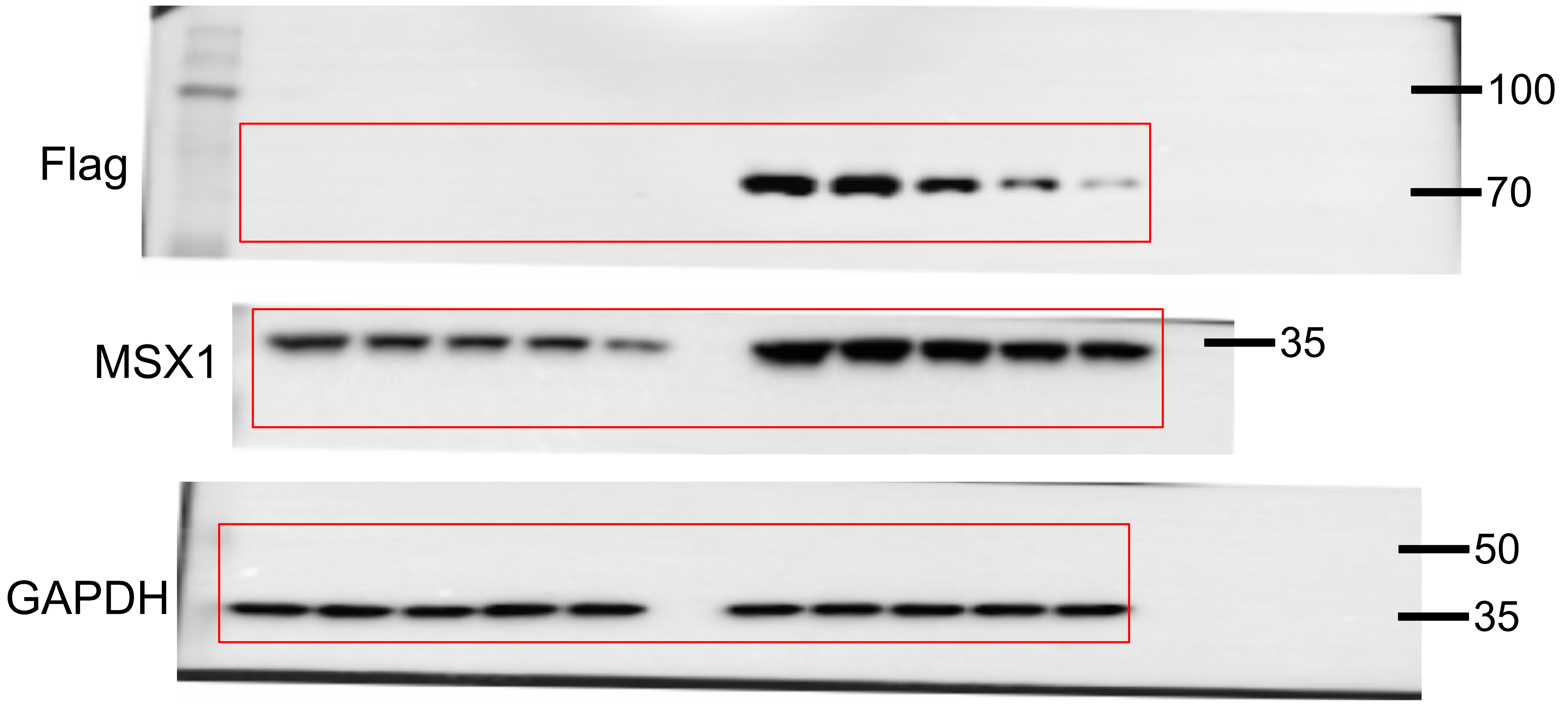

Uncropped blots of Supplementary Fig. S6

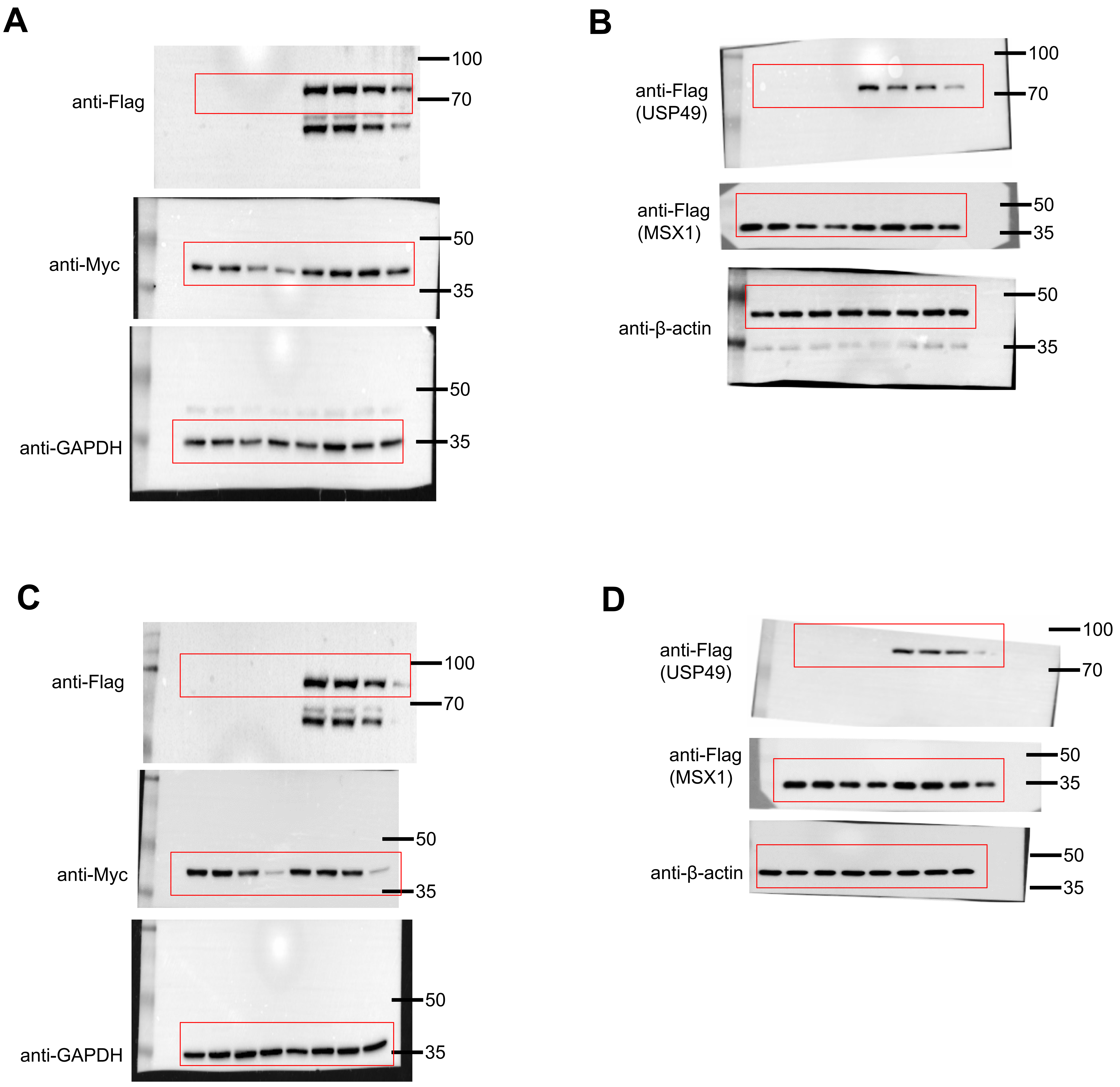

Uncropped blots of Supplementary Fig. S8

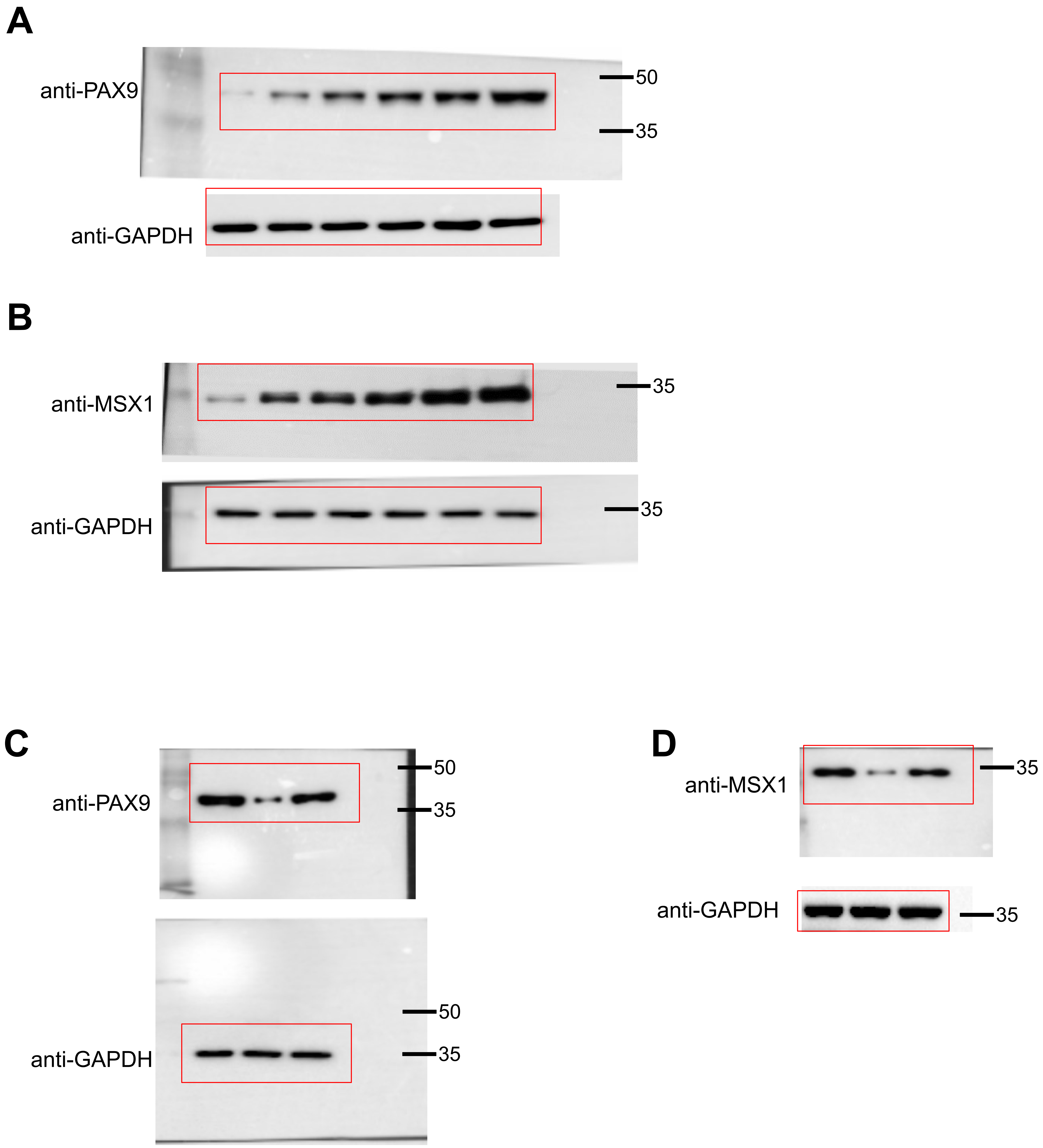

Uncropped blots of Supplementary Fig. S8

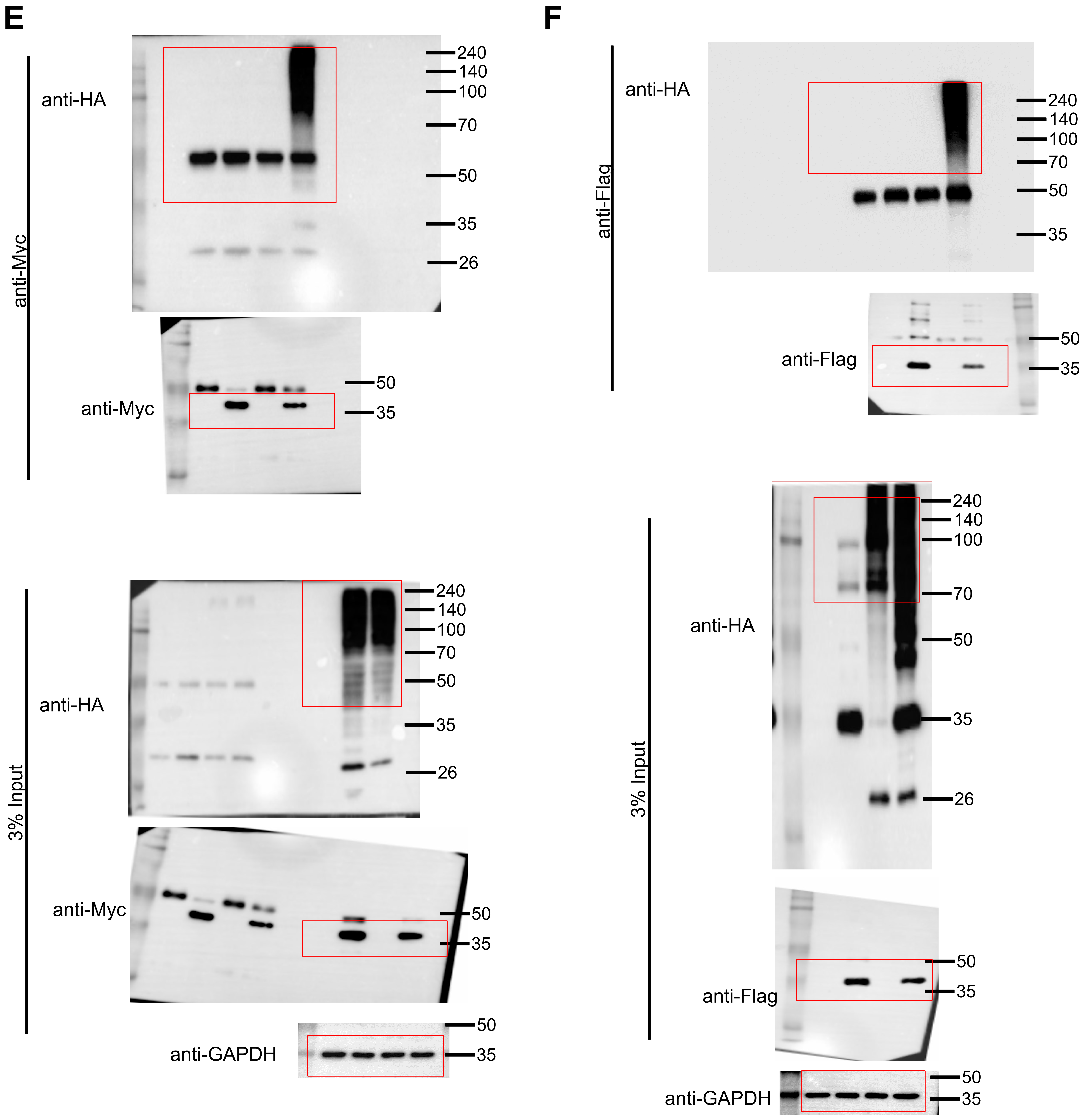

Uncropped blots of Supplementary Fig. S9

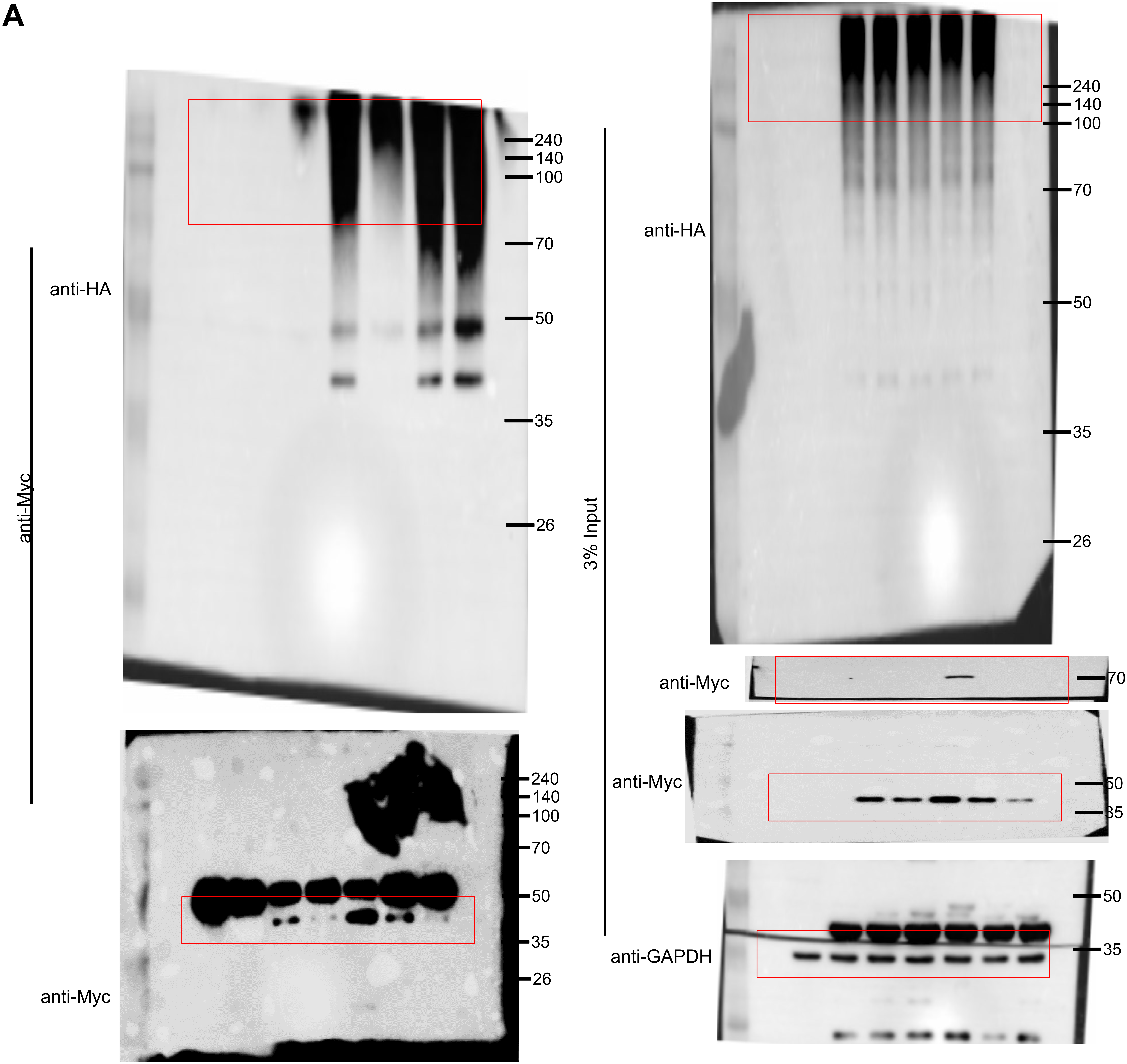

Uncropped blots of Supplementary Fig. S9

B

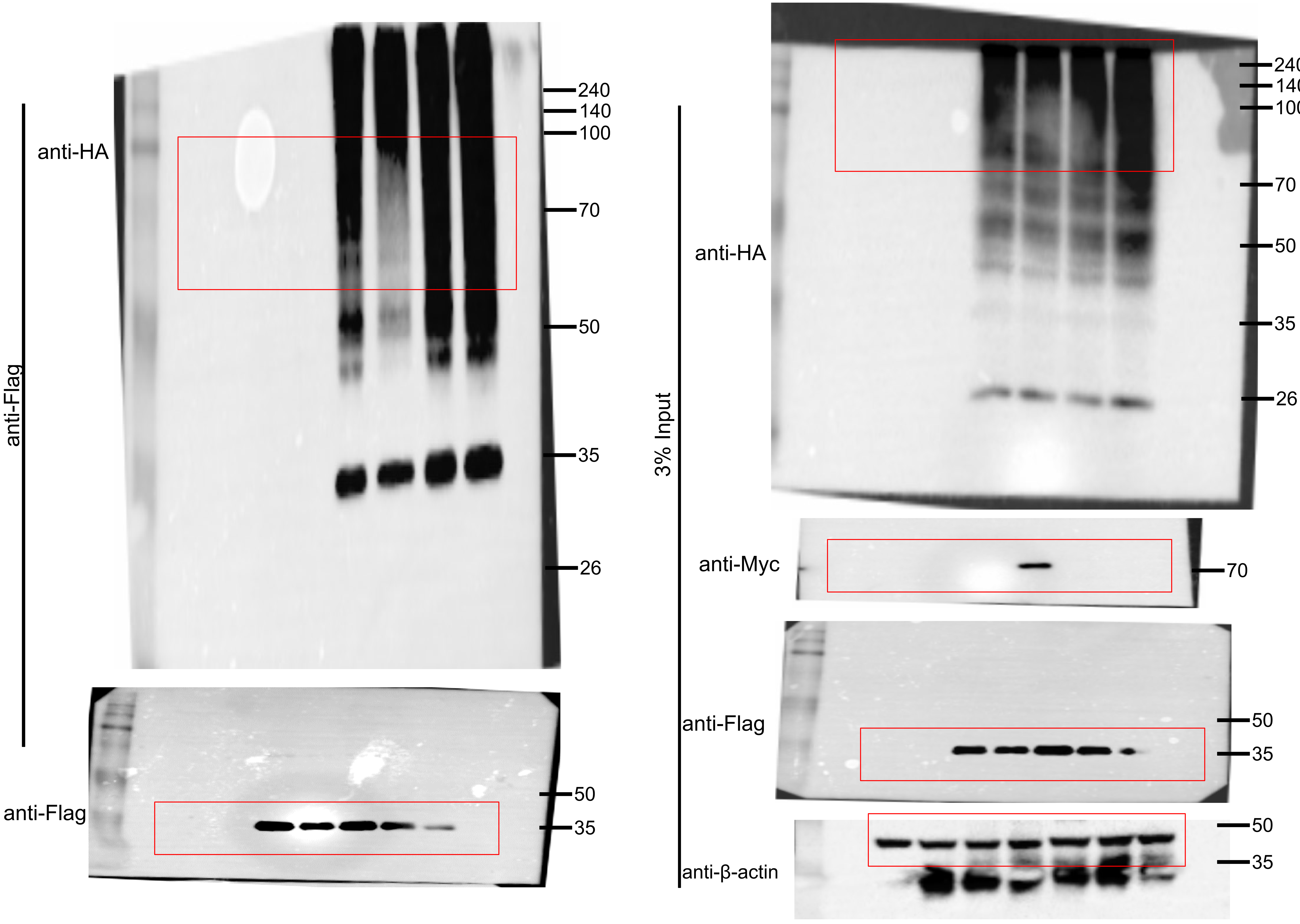

Uncropped blots of Supplementary Fig. S10

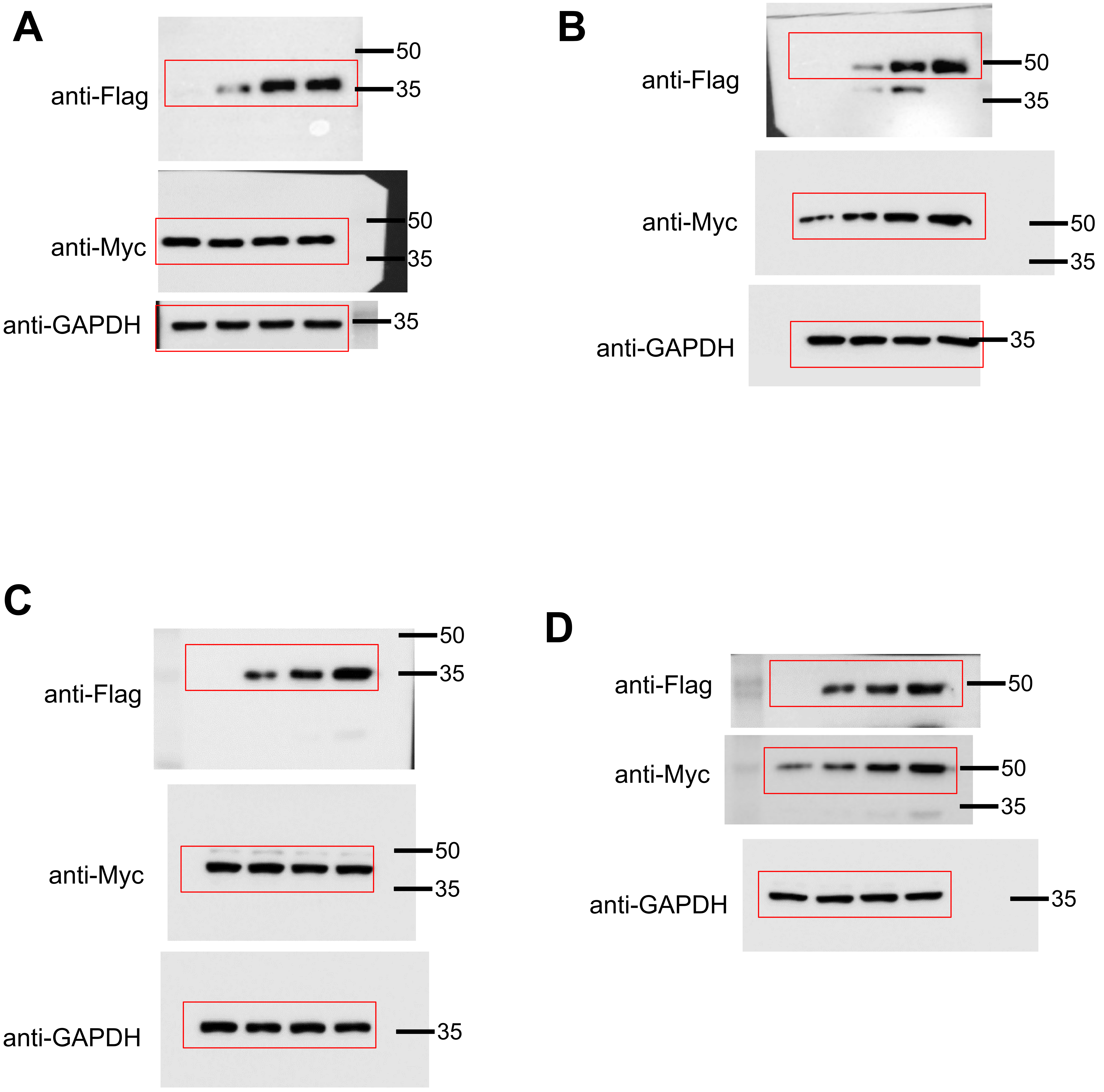

Uncropped blots of Supplementary Fig. S10

E

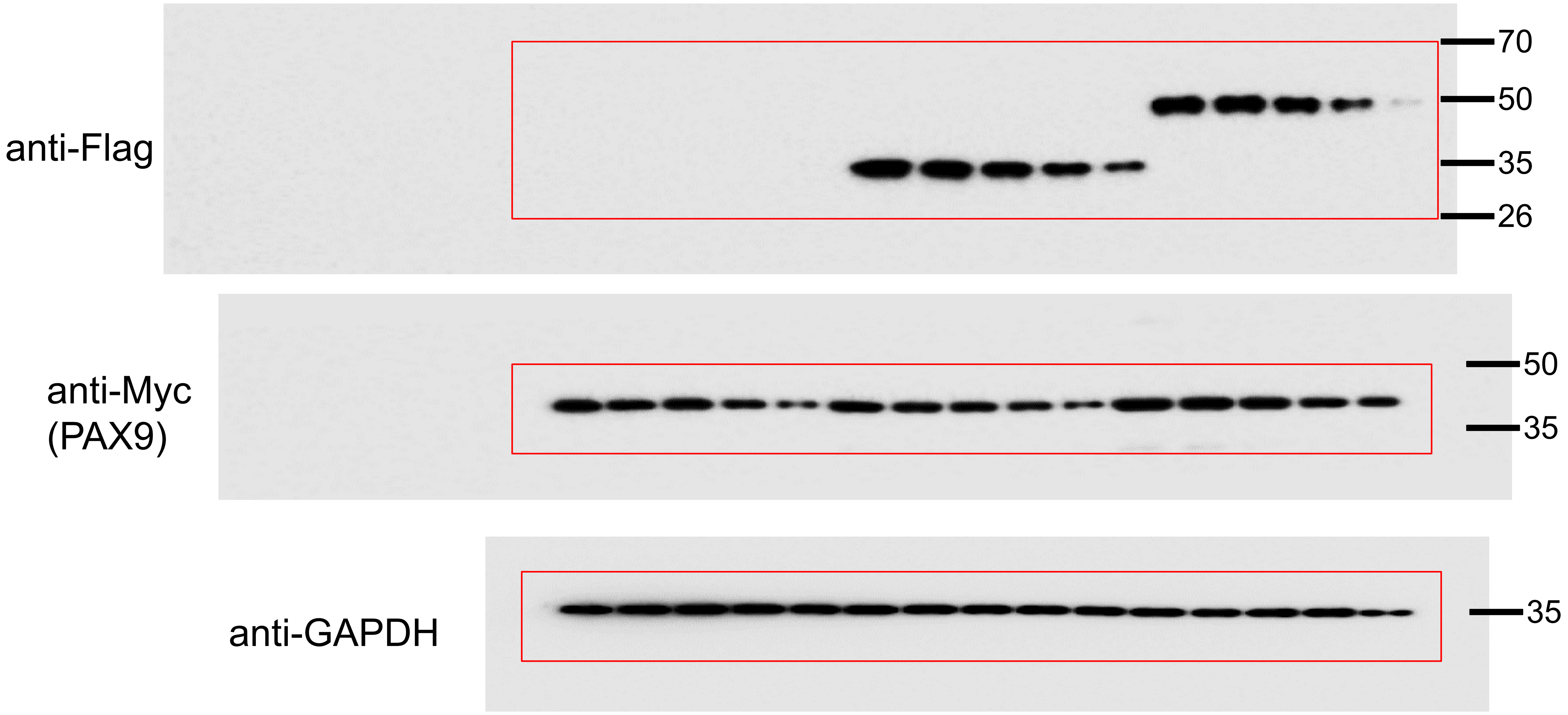

F

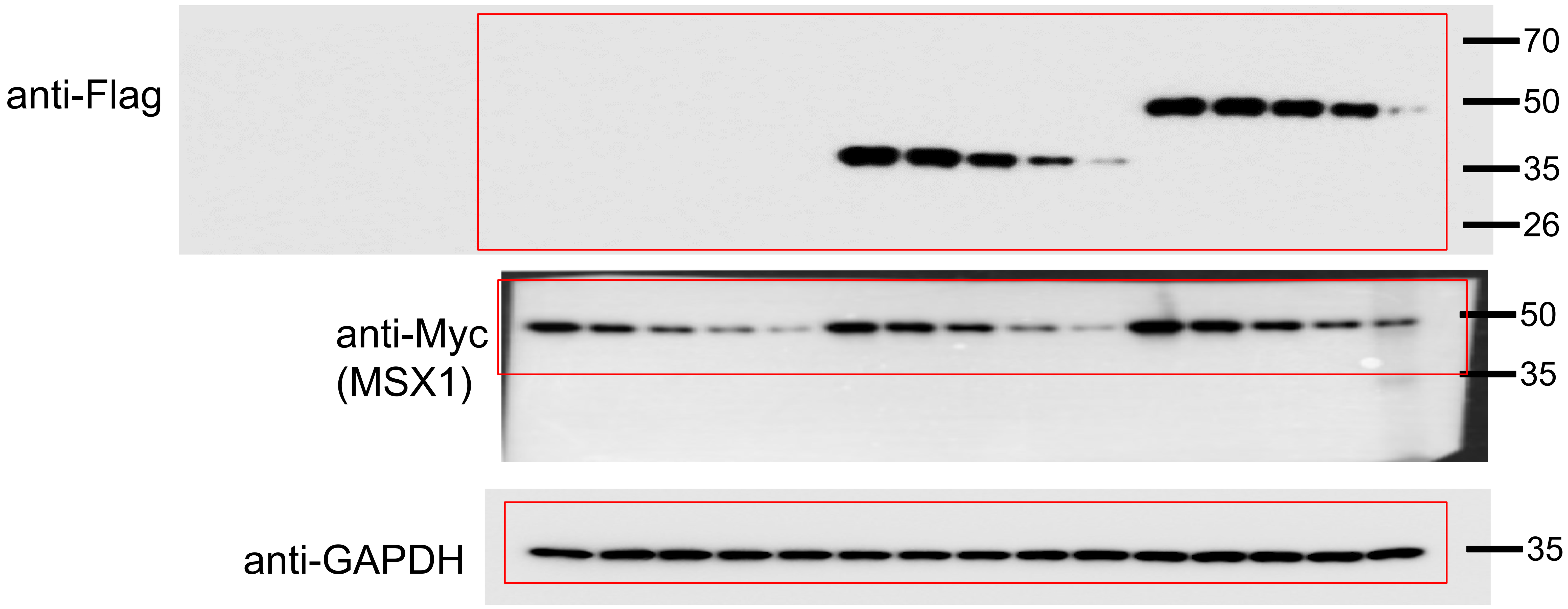

Uncropped blots of Supplementary Fig. S12

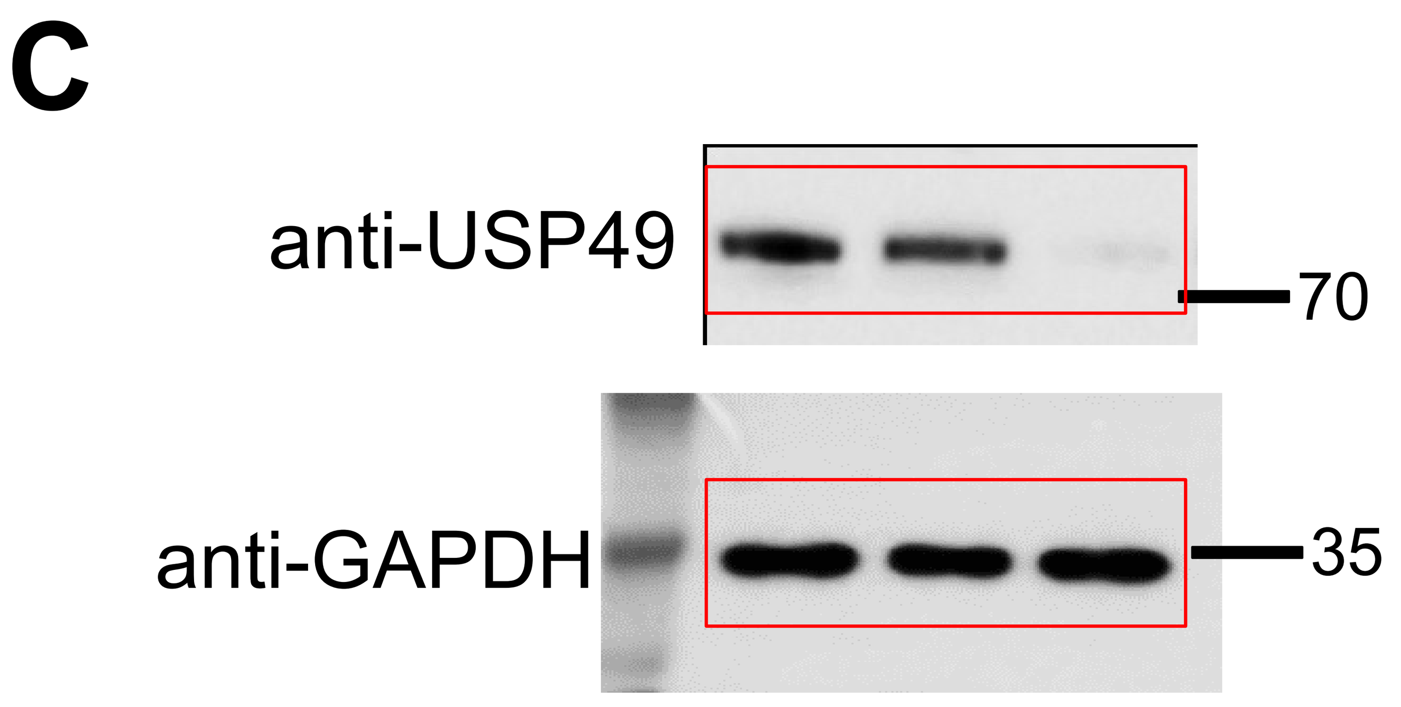

Uncropped blots of Supplementary Fig. S14

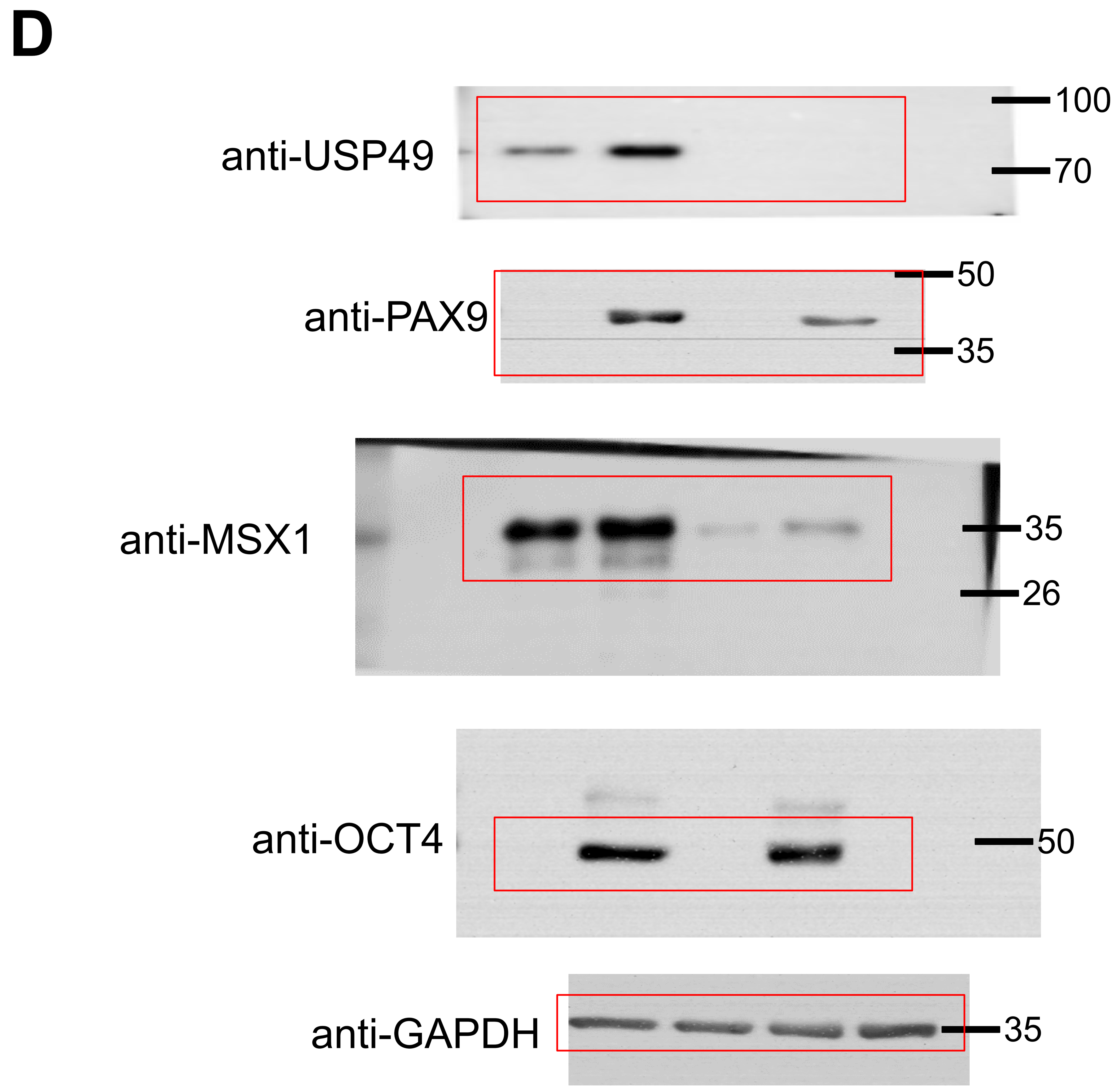

Supplement: Supplementary file 4 — Original Data File [file 41418_2022_956_MOESM4_ESM.pdf]
